# Pharmacogenomics of Cognitive Dysfunction and Neuropsychiatric Disorders in Dementia

**DOI:** 10.3390/ijms21093059

**Published:** 2020-04-26

**Authors:** Ramon Cacabelos

**Affiliations:** EuroEspes Biomedical Research Center, International Center of Neuroscience and Genomic Medicine, 15165 Bergondo, Corunna, Spain; rcacabelos@euroespes.com

**Keywords:** alzheimer’s disease, anxiety, behavioral disorders, depression, epilepsy, neuropsychiatric disorders, personalized medicine, pharmacogenomics, psychosis, sleep disorders

## Abstract

Symptomatic interventions for patients with dementia involve anti-dementia drugs to improve cognition, psychotropic drugs for the treatment of behavioral disorders (BDs), and different categories of drugs for concomitant disorders. Demented patients may take >6–10 drugs/day with the consequent risk for drug–drug interactions and adverse drug reactions (ADRs >80%) which accelerate cognitive decline. The pharmacoepigenetic machinery is integrated by pathogenic, mechanistic, metabolic, transporter, and pleiotropic genes redundantly and promiscuously regulated by epigenetic mechanisms. CYP2D6, CYP2C9, CYP2C19, and CYP3A4/5 geno-phenotypes are involved in the metabolism of over 90% of drugs currently used in patients with dementia, and only 20% of the population is an extensive metabolizer for this tetragenic cluster. ADRs associated with anti-dementia drugs, antipsychotics, antidepressants, anxiolytics, hypnotics, sedatives, and antiepileptic drugs can be minimized by means of pharmacogenetic screening prior to treatment. These drugs are substrates, inhibitors, or inducers of 58, 37, and 42 enzyme/protein gene products, respectively, and are transported by 40 different protein transporters. *APOE* is the reference gene in most pharmacogenetic studies. *APOE-3* carriers are the best responders and *APOE-4* carriers are the worst responders; likewise, CYP2D6-normal metabolizers are the best responders and CYP2D6-poor metabolizers are the worst responders. The incorporation of pharmacogenomic strategies for a personalized treatment in dementia is an effective option to optimize limited therapeutic resources and to reduce unwanted side-effects.

## 1. Introduction

Alzheimer’s disease (AD) is the most prevalent form of dementia (>50%), followed by vascular (VD), mixed dementia (MXD) (30–40%), and other modalities of neurodegenerative disorders (NDDs) (Lewy body dementia (LBD), frontotemporal dementia (FTD), prion dementia, Pick’s dementia, Parkinson–dementia complex (PDC); and comorbid FTD-amyotrophic lateral sclerosis) (5–10%). MXD shows the highest prevalence (>50%) in patients over 70–75 years of age. Genomic defects (Table 1), epigenetic aberrations, cerebrovascular dysfunction, and multiple environmental factors are the major risk factors that precipitate pathogenic cascades leading to the clinical phenotype of dementia which is characterized by progressive cognitive deterioration, behavioral changes, functional decline, and classical neuropathological hallmarks (extracellular Aβ deposition in senile plaques, intracellular neurofibrillary tangles with hyperphosphorylated tau, dendritic desarborization, and neuronal loss) [1,2,3,4,5,6]. The main focus of pharmacological research over the past 50 years has been the identification of cognitive enhancers; however, no US Food and Drug Administration (FDA)-approved drugs for AD have been reported for the past two decades [7]. Behavioral disorders (BDs) (psychotic, depressive, anxiety, sleep disorders, and inappropriate sexual behaviors) are common (10–90%) in patients with dementia and tend to increase in parallel with the cognitive deterioration [8,9,10,11,12,13,14]. BDs increase the risk of institutionalization, impair daily functioning, reduce quality of life, and accelerate cognitive deterioration [15,16]. BDs also increase the costs of dementia (e.g., LBD and VD) [17].

There is not a prototypical pattern of BDs in different dementia types; however, BDs tend to be more prevalent in FTD, in cases where the compromise of frontotemporal regions is more relevant [18,19,20], and in cases with mild traumatic brain injury (TBI) [21] where DNA damage-induced cellular senescence pathways have been identified [22]. Apathy, depression, dysphoria, agitation, aggression, hallucinations, and delusions are frequent distressing symptoms in dementia [14]. A current behavioral phenotype is the hyperactivity–impulsivity–irritiability–disinhibition–aggression–agitation complex, with a difficult set of symptoms to manage, causing an important psychological burden for caregivers and hospital staff [23]. Some neuropsychiatric disorders may increase the risk for late-onset dementia, and dementia may increase the risk for delayed-onset BDs in specific cases [24].

The primary causes of BDs in dementia are unclear. *APP*, *MAPT*, *APOE*, and other variants in pathogenic genes (Table 1) as well as the presence of schizophrenia- and/or depression-related SNPs [25,26,27], together with additional metabolic disorders [28], cerebrovascular risk or consolidated vascular damage [4,29,30,31], premorbid personality [32], and inappropriate management may contribute to BDs in AD. BDs partially correlate with conventional biomarkers of dementia [33,34]; however, agitation/aggression correlates with AD cerebrospinal fluid (CSF) biomarkers, and depression is inversely associated with core AD CSF pathology (low Aβ42, high Tau, and high pTau) [35,36]. Over 50% of AD patients show comorbidities (TDP-43 and Lewy bodies) which associate with frontotemporal lobar degeneration and LBD. Some of these comorbidities might explain BDs in dementia. TDP-43 is associated with aberrant psychomotor activity, and Lewy bodies are associated with anxiety, irritability, sleep disorders, and appetite anomalies [37]. In FTD, *C9orf72* hexanucleotide repeat expansion with more than 80 G4C2 repeats has been associated with high frequency of psychotic symptoms [38]. Limbic-predominant age-related TDP-43 encephalopathy with high pTau burden might also predispose to more severe cognitive deterioration and BDs [39].

Most BDs in dementia are susceptible to pharmacological intervention, and though some studies suggest that psychotropic medication does not accelerate cognitive decline [40], most studies indicate that inappropriate treatments and consequent adverse drug reactions (ADRs) are frequent and deleterious [41,42,43]. Current ADRs in the elderly population are associated with benzodiazepines, neuroleptics, antidepressants, and antihypertensives. These drugs may cause falls; delirium and excess mortality increase with polypharmacy; over-infections are frequent in patients with inappropriate use of broad-spectrum antibiotics; increased risk of stroke is observed in patients with dementia treated with antipsychotics; nonsteroidal anti-inflammatory drugs may cause hypertensive crises, bleeding, and cerebrovascular problems; and other ADRs have been extensively reported worldwide [43,44,45,46].

To palliate preventable ADRs, drug information resources have been developed. Some of them are designed for analyzing drug interactions, and others are useful to help physicians for an appropriate drug prescription [47,48,49,50,51]. However, few resources incorporate pharmacogenomics (PGx) as a practical tool for clinical use [45,52,53,54,55,56].

About 80% variability in drug pharmacokinetics and pharmacodynamics is attributed to PGx factors [56,57]. Rare variants contribute to approximately 30–40% of functional variability in 146 pharmagenes with clinical relevance. Over 240 pharmagenes are potentially associated with ADRs, and over 400 genes and their products influence drug efficacy and safety [53,54]. Furthermore, the pharmacological outcome is highly influenced by components of the PGx machinery, the chemical properties of each drug, and other diverse factors (e.g., compliance, nutrition, metabolic conditions, and concomitant drugs) [58,59].

The present review explores available information for personalized treatment of dementia in the areas of cognition and BDs based on PGx principles.

## 2. The Pharmacogenomic Machinery

The pharmacogenomic machinery is composed by a network of gene clusters coding for proteins and enzymes responsible for drug targeting and processing as well as critical components of the epigenetic machinery that regulate gene expression [60,61]. The pharmagenes involved in the pharmacogenomic response to drugs can be classified into five major categories: (i) Pathogenic genes (Table 1) which are associated with disease pathogenesis [62]; (ii) mechanistic genes coding for components of enzymes, receptor subunits, transmitters, and messengers associated with the mechanism of action of drugs; (iii) metabolic genes of different categories that encode phase I–II reaction enzymes responsible for drug metabolism. Phase-I reaction enzymes include (in alphabetical order) alcohol dehydrogenases, aldehyde dehydrogenases, aldo-keto reductases, amine oxidases, carbonyl reductases, cytidine deaminases, cytochrome P450 family (CYPs) of mono-oxygenases, cytochrome b5 reductase, dihydropyrimidine dehydrogenase, esterases, epoxidases, flavin-containing monooxygenases, glutathione reductase/peroxidases, peptidases, prostaglandin endoperoxide synthases, short-chain dehydrogenases, reductases, superoxide dismutases, and xanthine dehydrogenase. The most relevant Phase-II reaction enzymes include the following: amino acid transferases, dehydrogenases, esterases, glucuronosyl transferases, glutathione transferases, methyl transferases, N-acetyl transferases, thioltransferase, and sulfotransferases; (iv) transporter genes coding for drug transporters. The most relevant categories of transporters include the following: ATPase (P-type subfamily), V-type (vacuolar H^+^-ATPase subunit), and ATPase (F-type subfamily); ATP-binding cassette transporters (subfamily A) (ABC1), subfamily B (MDR/TAP), subfamily C (CFTR/MRP), subfamily D (ALD), subfamily E (OABP), subfamily F (GCN20), and subfamily G (WHITE); and solute carriers (high-affinity glutamate and neutral amino acid transporter family) (SLC); and (v) pleiotropic genes which encode proteins and enzymes involved in a great variety of metabolic cascades and metabolomic networks [6,43,56,61,62,63].

The expression or repression of all these genes and their products are regulated in a redundant and promiscuous fashion by the epigenetic machinery (DNA methylation/demethylation, histone/chromatin remodeling, and miRNA regulation), configuring the pharmacoepigenetic apparatus. The same enzyme/protein/transporter can process a multitude of drugs, and the same drug can be processed by a vast array of gene products in an orchestrated manner to operate as a security system against xenobiotic intruders [61,62,63,64,65,66,67]. 

A vast array of polymorphic variants in over 600 defective human genes are potentially involved in AD pathogenesis and drug response. The presence of the ε4 allele in the *APOE* gene is the most important risk factor among top pathogenic genes (Table 1) [1]. However, many other SNPs in diverse genes may contribute to AD-related neurodegeneration and premature neuronal death, including genes encoding components of the pharmacogenetic machinery. Polymorphic variants in ABC and SLC transporters may affect AD pathogenesis and response to drugs [3,63,68,69,70,71,72,73]. SNPs in genes encoding transporter proteins may affect brain penetrance and accessibility to neuronal/glial targets, drug metabolism, and drug resistance [70,74,75]. 

Mutations in ABC transporters affect pathogenesis and therapeutics in AD. The ABCB1 transporter protein (P-gp1) and other transporters of this category are located on endothelial cells lining brain vasculature. They play important roles in limiting the movement of substances into and enhancing their efflux from the brain. ABCB1 is a very active drug transporter in the brain. It is estimated that over 1270 drugs are directly or indirectly processed via the ABCB1 transporter protein P-gp. Approximately, 490 drugs are substrates, 618 are inhibitors, and 182 are inducers [55]. In Caucasians and African-Americans, 116 and 127 polymorphic sites, respectively, have been identified with a minor allele frequency greater than 5%. *ABCB1 C1236T* in exon 12, *G2677T/A* in exon 21, and *C3435T* in exon 26 are common variants. The ABCB1*13 haplotype involves 3 intronic SNPs (in intron 9, 13, and 14) and the 1236, 2677, and 3435 (*TTT*) SNPs. The *ABCB1 C1236T*, *G2677T/A*, and *C3435T* variants participate in the P-gp1 function at the blood–brain barrier (BBB). AD patients carrying *T* in *C1236T*, *G2677T*, and *C3435T* have exhibited higher binding potential values than *T* noncarriers. *ABCB1* variants might be potential biomarkers and might contribute to the progression of Aβ deposition in AD brains [76,77]. ABCB1 transports Aβ from the brain into the blood stream, and the cholesterol transporter *ABCA1* neutralizes Aβ aggregation in an *APOE*-dependent manner, facilitating Aβ elimination from the brain [78]. Other ABCs have shown potential association with AD [79]. The *ABCA7* (*G* allele) rs115550680 SNP has been associated with AD in Europeans, with a comparable effect to that of the *APOE-ε4* SNP rs429358 [80]. The *ABCA7* SNP rs200538373, with altered *ABCA7* exon 41 splicing, also shows association with AD risk [81]. *ABCA7* methylation might be a biomarker of AD [82]. In AD, *ABCA7* mRNA expression is higher than in controls, correlating with disease progression and cognitive decline. Alterations in lipid metabolism associated with *APOE-4* and several SNPs in *ABCA7* (rs3764650, rs3752246, and rs4147929) and loss-of-function mutations are pathogenic and PGx-dysfunctional in AD [83,84,85]. An intronic variable number tandem repeat (VNTR) in the *ABCA7* locus shows strong association with AD [86]. *ABCA7* variants cause accumulation of amyloid peptides and BBB dysfunction. *ABCA7* defects decrease APOE secretion and cholesterol exchange across the BBB [87]. Cholesterol-related genes such as *APOA5* (rs662799), *APOC1* (rs11568822), *APOD* (rs1568565), *CH25H* (rs13500), *LDLR* (rs5930), and *SORL1* (rs2282649), which affect lipid metabolism and membrane trafficking, may also be pathogenic and PGx-disruptive [63,88]. Soluble low-density lipoprotein receptor-related protein-1 (sLRP1), soluble receptor of advanced glycation end products (sRAGE), and transport proteins participate in the clearance of plasma Aβ in an *APOE*-dependent manner [89].

The ATP-binding cassette transporter *ABCA2* is an endolysosomal membrane protein with pleiotropic activities and a critical role in mediating sphingolipids and cholesterol trafficking [90]. *ABCA1* (rs2230805 and rs2230806) and *ABCA2* variants are associated with AD [91,92]. Upregulation of *ABCA2* mRNA expression has been observed in AD. Methylation of specific CpG islands in the *ABCA2* gene negatively associates with AD risk. *ABCA2* mRNA expression might also be used to differentially diagnose mild cognitive impairment (MCI) from other forms of dementia (i.e., Huntington’s disease) but not AD from MCI [93].

*ABCG2* is a transporter of large, hydrophobic, charged molecules and different toxic compounds. Dysfunctional *ABCG2* variants may affect absorption, distribution, accumulation, effectiveness, and toxicity of xenobiotic compounds and drugs [94]. *ABCG2* is upregulated in AD brains and is involved in Aβ transport. The *ABCG2-C421A* variant (rs2231142) (*ABCG2 C/C* genotype) is associated with AD. Interaction of the *ABCG2 C/C* genotype with the *APOE ε4* allele may increase AD risk [95].

Aβ alters BBB ABC efflux transporters and BBB permeability; specifically, *ABCB1*, *ABCC5*, and *ABCG2*; pregnane X receptor (*PXR*); and constitutive androstane receptor (*CAR*) transcription factors are inhibited by Aβ in brain endothelial cells [96].

Transporters encoded by genes of the solute carrier superfamily (SLC) and solute carrier organic (SLCO) transporter family are also important for AD pharmacogenomics. The human solute carrier (SLC) superfamily of transporters includes several hundred membrane-bound proteins with roles in physiological, pathological, and PGx processes. Over 200,000 exonic single-nucleotide variants (SNVs) have been identified, 99.8% of which are present in <1% of analyzed alleles. In the individual genome, there are about 29.7 variants with putative functional effects, and in specific populations, interethnic variability shows over 80% deleterious SLC variants [97]. 

In addition to *APP*, *PSEN1*, and *PSEN2* mutations, SNPs in membrane proteins that alter the transmembrane trafficking of products also influence pathogenesis and PGx. One example of this may be SORL1. *SORL1 (LR11)* gene variants are associated with AD. *SORL1* encodes a type I transmembrane 250-kDa protein (sorLA) that belongs to both the low-density lipoprotein receptor (LDLR) family and the vacuolar protein sorting 10 (VPS10) domain receptor family, acting as a sorting receptor for APP. SorLA, which interacts with ApoE and Tau, is a central regulator of trafficking and processing of APP and of Aβ destruction [98]. Another example might be the sarco/endoplasmic reticulum (SR/ER) calcium (Ca^2+^)-ATPase (SERCA) pump, an integral endoplasmic reticulum protein which has been associated with neuropsychiatric disorders (NPDs) and NDDs [99]. Translocation of substances across the mitochondrial membranes is required for cellular survival and efficient functioning. Major components of this translocation machinery are the translocase of the outer (TOMM) and inner mitochondrial membrane (TIMM) complexes. Mutations in the *TIMM8A (DDP)* and *DNAJC19 (TIMM14)* genes are pathogenic for Mohr–Tranebjærg syndrome and dilated cardiomyopathy syndrome, and polymorphisms in the *TOMM40* gene are associated with AD and other NDDs [100].

Transient receptor potential melastatin 2 (TRPM2) is a Ca^2+^-permeable nonselective cation channel of the TRP ion channel family. TRPM2 dysfunction linked to aberrant intracellular Ca^2+^ accumulation and neuronal death has been implicated in AD. TRPM2 is involved in the induction of N-methyl-D-aspartate (NMDA) receptor-dependent long-term depression, a form of synaptic plasticity at glutamate synapses [101].

Drug transporter expression is altered at the BBB and peripheral tissues in AD. Intestinal expression of multidrug resistance-associated protein 2 (Mrp2), monocarboxylate transporter 1 (Mct1), and UDP-glucuronosyltransferase (Ugt) and liver expression of Cyp51a1 and Cyp2c29 have been found altered in AD transgenic models [102].

## 3. CNS Drugs

**Figure 1:**
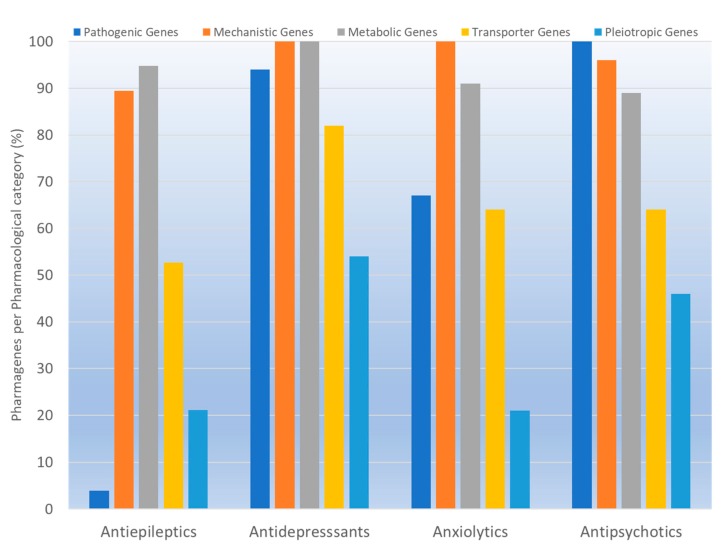
Pharmacogenetic machinery-related pathogenic, mechanistic, metabolic, transporter, and pleiotropic genes of antiepileptics, antidepressants, anxiolytics, and antipsychotics.

According to data available in the *World Guide for Drug Use and Pharmacogenomics* [55] and the *EuroPharmaGenics* (EPG) database [56] concerning central nervous system (CNS) drugs (Figure 1), the best-known genes of the pharmacogenetic machinery involved in the processing of antiepileptic, antidepressant, anxiolytic, hypnotic, sedative, antiparkinsonian, and antipsychotic drugs are mechanistic and metabolic genes, and poorly investigated genes are those involved in pathogenic mechanisms, transporters, and pleiotropic genes (Figure 1). Globally, 74% pathogenic, 97% mechanistic, 94% metabolic, 68% transporter, and 40% pleiotropic genes have so far been associated with CNS drug efficacy and safety [103]. 

**Figure 2:**
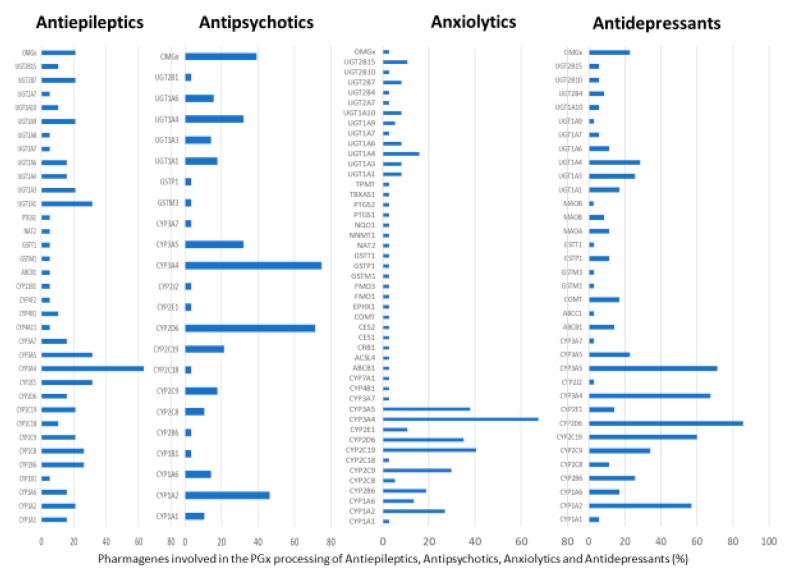
Substrates of antiepileptics, antipsychotics, anxiolytics, and antidepressants.

**Figure 3:**
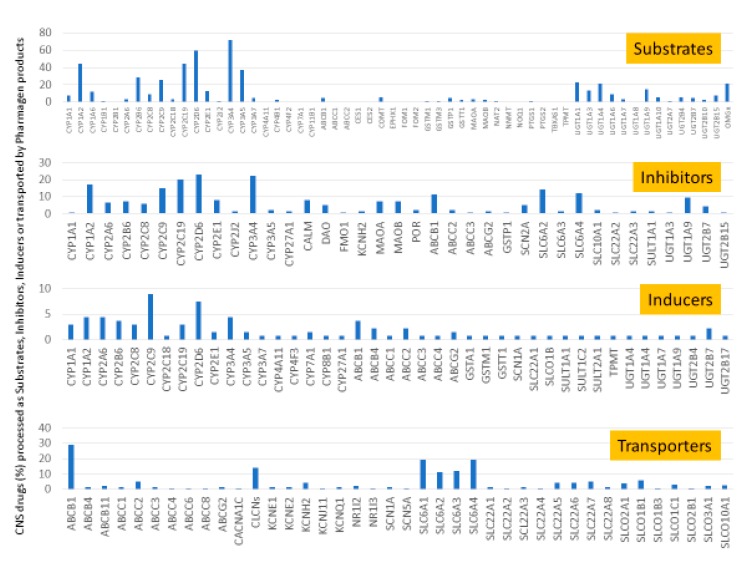
Substrates, inhibitors, inducers, and transporters of 307 CNS drugs (antidepressants, anxiolitics, hipnotics, sedatives, antipsychotics, antiepileptics, antiparkinsonian, and anti-dementia drugs).

CNS drugs can act as substrates, inhibitors, or inducers of enzymes encoded by metabolic genes (Figure 2). Among the 307 most frequently used CNS drugs, antiepileptics represent 14.66%, antiparkinsonians represent 10.42%, antipsychotics represent 21.82%, anxiolytics represent 11.40%, hypnotics and sedatives represent 21.17%, antidepressants represent 20.53%, and anti-dementia drugs represent 1%–2% (Figure 1 and Table 2). About 90% of these drugs use CYP enzymes as major metabolic pathways. CNS drugs are substrates, inhibitors, or inducers of 58, 37, and 42 enzyme/protein gene products, respectively, and are transported by 40 different protein transporters (Figure 3). CNS drugs are major substrates of CYP3A4 (71%), CYP3A5 (37%), CYP2D6 (60%), CYP2C19 (45%), and CYP1A2 enzymes (44%); inhibitors of CYP3A4 (22%), CYP2D6 (23%), CYP2C19 (20%), CYP1A2 (17%), and CYP2C9 (15%); and inducers of CYP2C9 (9%), CYP2D6 (7%), CYP3A4 (5%), CYP1A2 (4.5%), CYP2A6 (4.5%), and CYP2B6 (3.7%). Major transporters of CNS drugs are ABCB1 (29%), SLCA1 (20%), SLC6A4 (20%), CLCNs (15%), SLC6A3 (12%), and SLC6A2 (11%) (Figure 3) [103]. 

Approximately 80% of patients are deficient metabolizers for the tetrategic cluster integrated by *CYP2D6, 2C19, 2C9*, and *3A4/4* variants which encode enzymes responsible for the metabolism of 60–80% of drugs of current use, showing ontogenic-, age-, sex-, circadian- and ethnic-related differences. CYP geno-phenotypes differentiate extensive (EM; normal, NM), intermediate (IM), poor (PM), or ultra-rapid metabolizers (UM) with great geographic and ethnic variability worldwide [4,43,63].

The integration of *CYP2D6, CYP2C9, CYP2C19*, and *CYP3A4/5* variants into tetragenic haplotypes yields 156 geno-phenotypes. *H3 (1/1-1/1-1/1-3/3)* (20.87%) is the most frequent haplotype, representing full extensive metabolizers. Only 17 haplotypes exhibit a frequency higher than 1% in the Caucasian population. According to this, it is very likely that about 80% of individuals are deficient for the biotransformation of current drugs metabolized via CYP2D6-2C9-2C19-3A4 enzymes [4,43,63].

## 4. Pharmacogenomics of Cognition

Four acetylcholinesterase inhibitors have been approved for the treatment of AD. Tacrine was introduced in 1993 and discontinued years later due to hepatoxicity; Donepezil was introduced in 1996; Galantamine was introduced in 2001; and Rivastigmine was introduced in 2002. Memantine, an NMDA partial antagonist, was approved by the FDA in 2003 [104,105]. Over the past decade, the most prevalent pharmacological categories currently investigated as candidate strategies for the treatment of AD included neurotransmitter enhancers (11.38%), anti-Amyloid agents (13.30%), multi-target drugs (2.45%), anti-Tau agents (2.03%), and diverse natural products (25.58%). Some novel drugs (8.13%), novel targets (5.66%), revised old drugs (11.77%), anti-inflammatory drugs (1.20%), neuroprotective peptides (1.25%), stem cell therapy (1.85%), nanocarriers/nanotherapeutics (1.52%), and others (combination treatments, cognitive enhancers/nootropics, neurotrophic factors, polyunsaturated fatty acids, hormone therapy, epigenetic drugs, RNAi/gene silencing, miRNAs, and gene therapy) (<1% each) have also been investigated in exploratory studies for the treatment of AD [6]. However, no new drugs have been FDA-approved for the past 20 years. Consequently, most PGx studies concentrate on acetylcholinesterase inhibitors (AChEIs) (Donepezil, Galantamine, and Rivastigmine), Memantine, and combination treatments [2,3,106,107,108]. 

*APOE* gene variation is associated with major pathogenic events in AD [109,110], and *APOE* has been used as a reference gene in many clinical trials as a PGx marker, followed by metabolic genes (*CYP* geno-phenotypes) [1,2,4,56,106,107,111,112,113,114,115,116,117,118]. *APOE-4* carriers exhibit differential phenotypic patterns of acetylcholinesterase and butyrylcholinesterase activities as well as CYP enzyme activities with strong influence in PGx outcomes [119]. SNP variation in *CYP2D6*, acetylcholinesterase, butyrylcholinesterase, choline acetyltransferase, and paraoxonase is associated with better clinical response to AChEIs [120]. 

### 4.1. Donepezil

Donepezil is the most prescribed AChEI for the treatment of AD worldwide [107,121]. Donepezil is a major substrate of CYP2D6, CYP3A4, ACHE, and UGTs; inhibits ACHE and BCHE; and is transported by ABCB1 [120,122] (Table 2). Several *CYP2D6* variants may modify donepezil efficacy and safety in AD [55], and *APOE* and *CYP2D6* variants are determinant in the effects of donepezil. *APOE-4* carriers tend to be the worst responders, and *APOE-3* carriers are the best responders to donepezil in either monotherapy or drug combination regimes; CYP2D6-EMs are the best responders, and CYP2D6-PMs are the worst responders [1,2,4,56,106,111,112,113,114,115,116,117,118]. *CYP2D6* geno-phenotypes influence donepezil clearance. CYP2D6-PMs show a 32% slower elimination, and CYP2D6-UMs show a 67% faster elimination [123]. AD carriers of the common variant rs1080985 of *CYP2D6* show poor response to donepezil [122,124]. In Chinese patients, CYP2D6-EMs and PMs show a similar response to donepezil; however, EMs are better responders than UMs. Patients harboring the rs1080985 G allele are poor responders to donepezil, and the worst responders accumulate in carriers of the bigenic *APOE-4/rs1080985-G* genotype [125]. The mutated *CYP2D6* allele *2A is more frequent in responder than in nonresponder patients (75.38% vs. 43.48%). In Italian patients, 67% of the cases were responders, in whom abnormal enzymes accumulate, and 33% were nonresponders [126]. In Chinese and Thai AD cases, carriers of the mutant CYP2D6*10 allele responded better (58% responders) than carriers of the wild-type CYP2D6*1 allele [127]. The CYP2D6*10 variant strongly affects steady-state plasma concentration of donepezil and therapeutic outcome in Asian populations [128]. A recent study in China showed that CYP2D6*10 carriers treated with donepezil/galantamine have less side effects and that CYP2D6*10 carriers respond better to ChEIs [129].

Lower plasma donepezil concentration-to-dose ratios and better clinical response to donepezil have been reported in patients homozygous for the *T/T/T* genotype in the *ABCB1* haplotypes *1236C/2677G/3435C* (46%) and *1236T/2677T/3435T* (41%) [130]. There is also a better response to donepezil in *ABCA1 rs2230806 GG* carriers than in *AA* or *AG* carriers [131].

APOE-ε4/BCHE-K* carriers show an earlier age of onset, an accelerated cognitive decline, and a differential response to donepezil therapy [132]. Donepezil is not recommended in *BChE-K* and *APOE-4* carriers [133].

Donepezil is also used for the treatment of BDs in AD, LBD, and other dementia types [14,107,134]. Some reports indicate that AChEIs may also be beneficial in vascular dementia and cardiovascular disorders [135]. Donepezil might also ameliorate oxaliplatin-induced peripheral neuropathy [136] and confer protection against induced seizures in a mouse model (*Scn1a+/-*) of Dravet syndrome, an encephalopathy caused by de novo loss-of-function mutations in the SCN1A gene [137].

### 4.2. Galantamine

Galantamine is a major substrate of CYP2D6, CYP3A4, ABCB1, and UGT1A1 and an inhibitor of ACHE and BCHE. *APOE, APP, ACHE, BCHE, CHRNA4, CHRNA7*, and *CHRNB2* variants may also affect galantamine efficacy and safety [120,138,139,140,141]. Galantamine is mainly metabolized by CYP2D6 and CYP3A4 enzymes. Major metabolic pathways are glucuronidation, O-demethylation, N-demethylation, N-oxidation, and epimerization [142]. Galantamine is a substrate of ABCB1. *CYP2D6* variants are major determinants of galantamine pharmacokinetics, with CYP2D6-PMs presenting 45% and 61% higher dose-adjusted galantamine plasma concentrations than heterozygous and homozygous CYP2D6-EMs [120,143]; however, these pharmacokinetic changes might not substantially affect pharmacodynamics [144]. The coadministration of galantamine with CYP2D6 and CYP3A4 strong inhibitors increases its bioavailability [48,145]. Galantamine bioavailability and its therapeutic effects may be modified by interaction with foods and nutritional components [146].

Some recent studies show promising results with galantamine in cases of drug abuse (opioids, cocaine, and cannabis) [147,148] and TBI [149]. In combination with CDP-Choline, memantine, and antipsychotics, galantamine might be useful in schizophrenia [150,151].

### 4.3. Rivastigmine

Rivastigmine is a dual inhibitor of acetylcholinesterase (AChE; EC 3.1.1.7) and butyrylcholinesterase (BuChE; EC 3.1.1.8) in AD [152,153]. *APOE, APP, CHAT, ACHE, BCHE, CHRNA4, CHRNB2*, and *MAPT* variants may affect rivastigmine pharmacokinetics and pharmacodynamics. CYP enzymes are not involved in the metabolism of rivastigmine [120,138,145,154]. UGT2B7-PMs show higher rivastigmine levels with a poor response to treatment [155]. In combination treatments with memantine, carriers of CYP2D6*3, UGT2B7, and UGT1A9*5 variants show differential responses to treatment. Two SNPs on the intronic region of *CHAT* (rs2177370 and rs3793790) [156] and *CHRNA7* variants may influence the response to AChEIs [157]. Two SNPs, one intronic marker in *PRKCE-rs6720975* and an intergenic *NBEA-rs17798800* marker, might also contribute to differential therapeutic response to AChEIs [158]. Females with the *BChE-wt/wt* show a better benefit with rivastigmine than males, and BChE-K* male carriers show a faster cognitive decline than females [159]. AD patients harboring the *BChE K*-variant (rs1803274), causing a reduced enzyme activity, show low clinical response to rivastigmine. The K-variant (p. A539T) and other SNPs located outside the coding sequence in 5’UTR (rs1126680) and/or intron 2 (rs55781031) of the *BCHE* gene are responsible for reduced enzyme activity and poor response to rivastigmine [160].

Rivastigmine may be useful in VD and Parkinson’s disease [161,162] and in combination with low-dose quetiapine can improve psychotic symptoms in LBD [163].

### 4.4. Memantine

Memantine is an N-Methyl-D-Aspartate (NMDA) receptor antagonist which binds preferentially to NMDA receptor-operated cation channels [108]. Memantine inhibits the actions of glutamate via NMDA receptors and antagonizes GRIN2A, GRIN2B, GRIN3A, HTR3A, and CHRFAM7A. *APOE, PSEN1*, and *MAPT* are pathogenic genes which might influence the effects of memantine in AD, and variants in some mechanistic genes (*GRIN2A, GRIN2B, GRIN3A, HTR3A, CHRFAM7A, c-Fos, Homer1b*, and *PSD-95*) may also modify its therapeutic effects. CYP2B6 and CYP2D6 are strongly inhibited by memantine. In contrast, CYP1A2, CYP2A6, CYP2C9, CYP2C19, CYP2E1, and CYP3A4 are weakly inhibited [55,120,164,165]. Studies in human liver microsomes show that memantine inhibits CYP2B6 and CYP2D6; decreases CYP2A6 and CYP2C19; and has no effect on CYP1A2, CYP2E1, CYP2C9, or CYP3A4 [166]. The coadministration of CYP2B6 substrates decreases memantine metabolism by 65%. *NR1I2* rs1523130 is the only genetic covariate for memantine clearance in clinical studies. *NR1I2 rs1523130 CT/TT* carriers show a slower memantine elimination than carriers of the *CC* genotype [165]. NMDA receptors are glutamate receptors with Mg^2+^-mediated voltage-dependence effects in synaptic plasticity. Mutations in NMDA receptor subunits are present in NDDs. Patients with severe epileptic encephalopathy harbor the missense variant *GluN2AN615K (GRIN2A C1845A)*, which affects NMDA receptor channel blockers, including memantine [167].

Memantine can be used alone or in combination with AChEIs in AD [168,169]. Proteomic studies in the hippocampus and the cerebral cortex of AD-related transgenic mice (3× Tg-AD) treated with memantine revealed alterations in the expression of 233 and 342 proteins, respectively [170]. In *APP23* transgenic mice with cerebral amyloid angiopathy (CAA), memantine reduces cerebrovascular Aβ and hemosiderin deposits by enhancing Aβ-cleaving IDE expression [171].

Memantine is useful for the treatment of both cognitive deterioration and BDs in AD and other forms of dementia [172,173]. AD patients treated with memantine plus citalopram show improvement in BDs [174]; in combination with antipsychotics, it may improve verbal memory, learning, verbal letter fluency, and working memory with no effect on psychotic symptoms in patients with chronic schizophrenia [175,176]; at a dose of 20 mg/day, it may be effective in patients with obsessive-compulsive disorder [177]; at 10 mg/day, it may be helpful in migraine [178]; and, in combination with naltrexone, it enhances the efficacy of naltrexone in reducing alcohol drinking and craving [179]. Memantine might also be useful in the treatment of punctate and dynamic allodynia by the blockade of the microglia Kir2.1 channel to suppress microglia activation [180], and in combination with dextromethorphan, it may be beneficial in neuropathic pain [181].

### 4.5. Multifactorial Treatments

**Figure 4:**
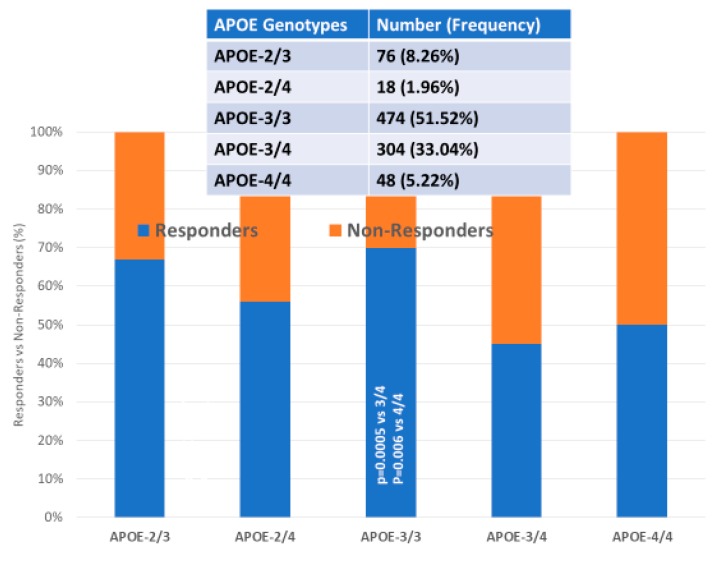
APOE-related responder/nonresponder rate in patients with Alzhimer’s disease treated with a multifactorial/combination treatment for one year. Responders: Final Mini-Mental State Examination (MMSE) score > basal MMSE score. NonResponders: Final MMSE score < basal MMSE score. Data Source: Cacabelos et al., 2014.

**Figure 5:**
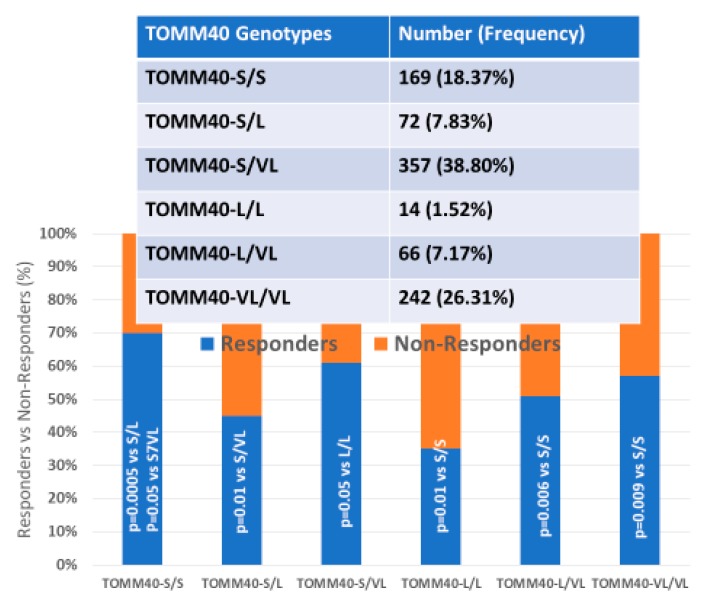
TOMM40-related responder/nonresponder rate in patients with Alzhimer’s disease treated with a multifactorial/combination treatment for one year. Responders: Final MMSE score > basal MMSE score. Nonresponders: Final MMSE score < basal MMSE score. Data Source: Cacabelos et al., 2014.

**Figure 6:**
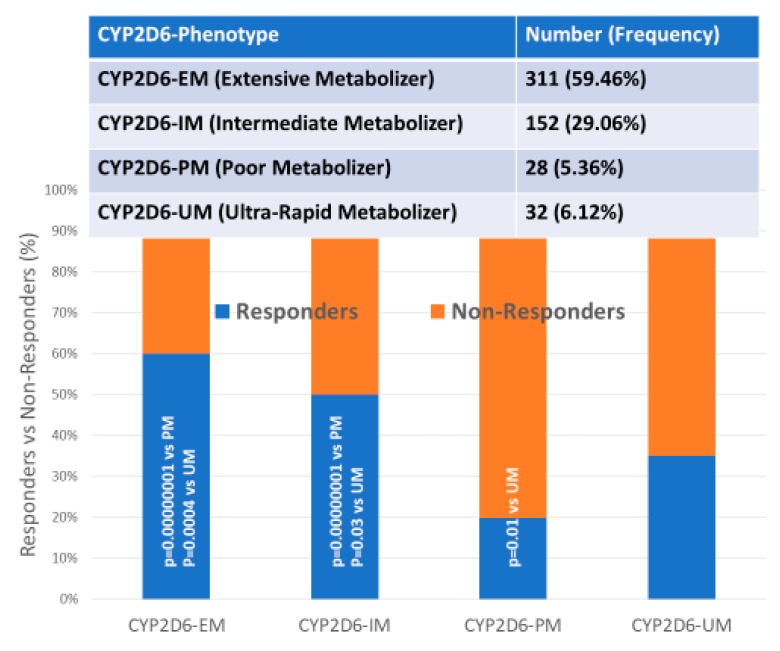
CYP2D6-related responder/nonresponder rate in patients with Alzhimer’s disease treated with a multifactorial/combination treatment for one year. Responders: Final MMSE score > basal MMSE score. Nonresponders: Final MMSE score < basal MMSE score. Data Source: Cacabelos et al., 2019.

Most studies in which AD patients are treated with multifactorial combinations reveal that *APOE-3/3* carriers are the best responders and that *APOE-4/4* carriers are the worst responders. Concerning CYP-related PGx outcomes, CYP2D6-EMs are the best responders, CYP2D6-PMs are the worst responders, and CYP2D6-IMs and UMs show an intermediate response [1,2,3,56,71,106,112,115,116,118] (Figure 4, Figure 5 and Figure 6).

In linkage disequilibrium with and adjacent to the *APOE* locus (19q13.2) is the *TOMM40* gene, which encodes an outer mitochondrial membrane translocase involved in the transport of Aβ and other proteins into mitochondria. A poly T repeat in an intronic polymorphism (rs10524523) (intron 6) in the *TOMM40* gene has been implicated in AD pathogenesis and PGx. The number of “T”-residues in the rs10524523 (“523”) locus differentiates 3 allele groups: “short” (S, T ≤ 19), “long” (L, 20 ≤ T ≤ 29), and “very long” (VL, T ≥ 30). *rs10524523 L* variants are associated with a higher risk for late-onset AD (LOAD). *APOE-TOMM40* interactions affect the risk of AD and the response to drugs. The *S/VL* and *VL/VL TOMM40 poly T* genotypes interact with all *APOE* genotypes; however, the *APOE-4/4-TOMM40-L/L* association is unique, accounting for 30% of *APOE-4/4* carriers. *TOMM40 poly T-S/S* carriers are the best responders, *VL/VL* and *S/VL* carriers are intermediate responders, and *L/L* carriers are the worst responders to treatment. *TOMM40-L/L* and *S/L* carriers in haplotypes with *APOE-4* are the worst responders to treatment. *TOMM40-S/S* carriers and, to a lesser extent, *TOMM40-S/VL* and *TOMM40-VL/VL* carriers in haplotypes with *APOE-3* are the best responders to treatment. The *TOMM40-L/L* genotype is exclusively associated with the *APOE-4/4* genotype in 100% of the cases, and this haplotype (*4/4-L/L*) might be responsible for premature neurodegeneration and consequent early onset of the disease, a faster cognitive deterioration, and a limited response to conventional treatments [4,116] (Figure 5). 

Epigenetic factors are important in AD pathogenesis and response to treatment [5,26,57,61,65,71]. Sirtuin variants may alter the epigenetic machinery, contributing to AD pathogenesis. The *SIRT2-C/T* genotype (rs10410544) (50.92%) has been associated with AD susceptibility in the *APOEε4*-negative population (*SIRT2-C/C*, 34.72%; *SIRT2-T/T* 14.36%). *SIRT2-APOE* bigenic clusters yield 18 haplotypes that influence the PGx outcome. *APOE-3/4* and *APOE-4/4* genotypes accumulate in *SIRT2-T/T* > *SIRT2-C/T* > *SIRT2-C/C* carriers, and *SIRT2-T/T* and *SIRT2-C/T* genotypes accumulate in *APOE-4/4* carriers. *SIRT2-C/T* carriers tend to be the best responders, *SIRT2-T/T* carriers are intermediate responders, and *SIRT2-C/C* carriers are the worst responders to a multifactorial treatment. PGx outcomes related to *APOE-SIRT2* bigenic clusters show that *33CC* carriers respond better than *33TT* and *34CT* carriers, whereas *24CC* and *44CC* carriers are the worst responders. SIRT2-C/T-CYP2D6-EMs are the best responders [5].

## 5. Pharmacogenomics of Neuropsychiatric Disorders

### 5.1. Psychotic Disorders

Psychotic symptoms (hallucinations and delusions) are strongly related to AD-associated cognitive dysfunction, gradually progressing in parallel with disease severity [182]. In addition, symptoms of personality changes, paranoia, hallucinations, cravings, agitation, and changes in appetite may represent a prodromal noncognitive phenotype of risk for dementia [183]. 

Over 500 genes are potentially associated with psychosis and schizophrenia (SCZ) [25]. Several BDs in dementia incorporate SCZ genes. Major biological pathways and mechanisms associated with SCZ genes include glutaminergic receptors (GRIA1, GRIN2, GRIK4, and GRM5), serotonergic receptors (HTR2A and HTR2C), GABAergic receptors (GABRA1 and GABRB2), dopaminergic receptors (DRD1 and DRD2), calcium-related channels (CACNA1H and CACNA1B), and solute carrier transporters (SLC1A1 and SLC6A2) [184]. Aberrant motor behavior is strongly associated with the *APOE-4/4* genotype and the presence of both Lewy bodies and cerebral amyloid angiopathy [185]. 

Antipsychotics are drugs of current use (>50%) to treat BDs in dementia with limited efficacy in aggressive behaviors and psychotic symptoms. These drugs increase mortality and risk of psychomotor disorders and cerebrovascular events. Neuroleptics are prescribed for long periods of time in combination with antidepressants and anti-dementia drugs [186,187].

Antipsychotics (Table 3) are associated with the PGx activity of less than 100 pharmagenes. The different pharmacological categories of antipsychotics (phenothiazines with aliphatic side-chain, piperazine-related phenothiazines, piperidine phenothiazines, butyrophenones, indole derivatives, thioxanthenes, diphenylbutylpiperidines, diazepines, oxazepines, thiazepines, oxepines, benzamides, and other neuroleptics) are substrates, inhibitors, or inducers of 32, 16, and 3 enzyme/protein gene products, respectively, and are transported by at least 14 different protein transporters. CYP enzymes participate in the metabolism of 90% of antipsychotics. These drugs are major substrates of CYP3A4 (75%), CYP2D6 (72%), CYP1A2 (46%), CYP2C19 (22%), and UGT1A4 (33%); inhibitors of CYP2D6 (50%), CYP3A4 (42%), ABCB1 (29%), CYP1A2 (25%), CYP2C19 (21%), and CYP2C9 (15%); and inducers of GSTM1, MAOB, and SLCO3A1 with low affinity (<5%). About 50% of antipsychotics are transported by ABCB1. Other transporters responsible for the influx-efflux of neuroleptics are KCNH2 (22%); KCNE1 and KCNE2 (18%); KCNQ1 (18%); and SLC6A2, SLC6A4, and SCN5A (11%). Haloperidol is associated with 31 pharmagenes, Olanzapine is associated with 28, and Thioridazine is associated with 27 [55,103].

The dopamine transporter (*SLC6A3*) gene has been the focus of attention for years in the PGx of antipsychotic drugs. The study of 6 SNPs (rs2652511 (*T-844C*) and rs2975226 (*T-71A*) in the 5’-regulatory region, rs2963238 (*A1491C*) in intron 1, a *30-bp VNTR* in intron 8, rs27072 and the *40-bp VNTR* in the 3’-region) showed association of allele and genotype frequencies with response to clozapine [188].

Major ADRs with antipsychotics are extrapyramidal symptoms, tardive dyskinesia, antipsychotic-induced weight gain, and clozapine-induced agranulocytosis. Antipsychotic-induced extrapyramidal symptoms are associated with *DRD2, SLC18A2, HTR2A, GRIK3*, and *SLC6A3 VNTR* and *COMT Val158Met* polymorphisms [189,190]. SNPs in *ADORA1, ADORA2A*, and *ADORA3* have been associated with psychopathological symptoms, extrapyramidal symptoms, akathisia, and tardive dyskinesia [191]. RGS2*T/*T(rs2746073), RGS2*C/*C (rs4606), and RGS2*A/*A (rs2746071) are associated with high risk for extrapyramidal symptoms in Russian patients treated with haloperidol [192]. Several SNPs in genes of the mTOR pathway (*AKT1, rs1130214; FCHSD1, rs456998; Raptor, rs7211818;* and *DDIT4, rs1053639*) have also been associated with extrapyramidal disorders in Spanish patients under antipsychotic treatment [193].

Tardive dyskinesia (TD) is an involuntary movement disorder that occurs in over 20% of patients after chronic treatment with antipsychotics. Disrupted in schizophrenia 1 (*DISC1*) gene variants, SNP variation in several CYPs, especially *CYP2D6*, and in *HSPG2, CNR1, DPP6, LC18A2, MTNR1A, PIP5K2A, DRD2, DRD3, VMAT2, HSPG2, HTR2A, HTR2C*, and *SOD2* variants influence TD [194,195,196]. An intron-1 SNP (rs6977820) of the *DPP6* (dipeptidyl peptidase-like protein-6) gene has been associated with TD in Japanese patients. DPP6 is an auxiliary subunit of Kv4 that regulates the activity of dopaminergic neurons. Decreased expression of *DPP6* in the brain can be reversed by haloperidol treatment [197]. Another SNP (rs2445142) in the *HSPG2* (heparan sulfate proteoglycan 2) gene has been associated with TD in both Japanese and Caucasian populations [198]. Carriers of the *CNR1-rs806374 (T>C) CC* genotype are more likely to develop TD and severe motor dysfunction than *TT* or *TC* among Caucasians. Cannabinoid receptor 1 (CNR1) activators inhibit movement, and this effect is prevented by rimonabant and selective CNR1 antagonists [199]. A number of SNPs in the *SLC18A2* gene that encodes VMAT2 (vesicular monoamine transporter 2) may affect TD, including the rs2015586 marker (with deleterious effects) and the rs363224 marker (with protective effects in carriers of the low-expression *AA* genotype) [200]. Two VMAT2 inhibitors, Valbenazine and Deutetrabenazine, have been approved for treating TD [201,202,203].

Hyperprolactinemia is a common ADR in users of neuroleptic drugs. Risperidone-induced prolactin response is associated with 3 *UGT1A1* SNPs (UGT1A1*80c.-364C>T, UGT1A1*93 c.-3156G>A, and *UGT1A1 c.-2950A>G*) [204].

Antipsychotic-induced weight gain occurs in over 30–40% of patients with SCZ. All antipsychotics are associated with weight gain in antipsychotic-naïve and first-episode patients; however, weight gain is greatest in patients treated with the second-generation antipsychotics clozapine and olanzapine. The proportion of patients with body weight gain (>7% baseline) is >20% for ziprasidone, >30% for quetiapine, and 45% for aripiprazole [205]. This ADR is caused by different factors: pharmacological properties of the drug, pharmacogenetic factors, environmental factors, and ethnicity [206].

Over 100 permutated pathways may be involved in weight gain regulation in response to neuroleptics, and Genome-wide association studies (GWAS) analysis revealed that Peroxisome proliferator activated receptor gamma (PPARG) and Proprotein convertase, subtilisin/hexin-type 1 (PCSK1) are involved in antipsychotic-induced weight gain [207]. Genetic associations between pathogenic genes and genes involved in lipid metabolism cannot be ruled out as feasible links in body weight changes in response to psychotropic treatments. In fact, lipoprotein lipase (LPL), a key enzyme in triglyceride hydrolysis, is expressed in brain regions which are structural substrates of higher activities of the CNS (learning, memory, behavior, cognition), and SNPs in the *LPL* gene locus (8p22) (*rs253 C* allele) have been associated with SCZ [208].

Stimulation of the serotonin (5-HT)1A receptor (HTR1A) contributes to the mechanism of action of clozapine and lurasidone. rs358532 and rs6449693, tag SNPs for rs6295, may predict response to lurasidone [209]. The CYP1A2*1F/*1F genotype has been associated with clozapine-induced generalized tonic-clonic seizures in Brazilian patients [210]. The study of 6 SNPs in the tryptophan hydroxylase (*TPH*) gene (rs4570625, rs11178997, rs11178998, rs7954758, rs7305115, and rs4290270) revealed association of the rs10784941 and rs4565946 markers and the rs4570625-rs4565946 haplotype with SCZ. *TPH2* variants and the *rs4570625-rs4565946 G-C* haplotype do not affect response to antipsychotic drugs [211].

Antipsychotic-related ADRs can be substantially reduced with the incorporation of PGx procedures into clinical practice [27,43,103].

### 5.2. Depressive Disorders

Depression is the second most common psychiatric symptom in AD, after apathy. The prevalence of depression in AD varies from 5% to >40% in different studies. Patients with severe AD show a higher prevalence of depression [212,213] and depressive symptoms are associated with a faster rate of memory decline [214]. There is evidence that early life depression can be a risk factor for later life dementia and that later life depression may represent a prodrome to dementia [215], with sex-specific differences [216], especially in cases with cerebral amyloidopathy (>50%) [217]. Furthermore, depressed patients with mild cognitive impairment (MCI) have worse cognitive performance and greater loss of gray-matter volume in the cerebellum and parahippocampal gyrus [218]. Low psychomotor speed has also been associated with an increased risk of developing dementia and depressive symptoms [219]. Cardiovascular risk factors may contribute to depression in patients with MCI [220]. Late-life depression (LLD) affects 15% of the elderly population. *CYP2D6*, *SLC6A4 5-HTTLPR*, and brain-derived neurotrophic factor (*BDNF*) variants influence the PGx of this condition, as recently reported by the Clinical Pharmacogenetics Implementation Consortium [221].

Some studies indicate that the *APOE* genotype may influence the incidence of BDs and treatment in dementia [111,117], while others did not find association of *APOE-4* with depression, anxiety, apathy, agitation, irritability, or sleep disturbances in cognitively impaired subjects [222]. Genome-wide association studies (GWAS) identified 31 genes located in 19 risk loci for major depressive disorder (MDD). Common and rare variants of *L3MBTL2* are associated with AD. mRNA expression levels of *SORCS3* and *OAT* are differentially expressed in AD brain tissues, and 13 MDD risk genes may interact with core AD genes such as *HACE1, NEGR1*, and *SLC6A15* [223].

The treatment of depression in dementia can be pharmacological, with antidepressants, especially selective serotonin reuptake inhibitors as a first choice, or nonpharmacological (emotion-oriented therapies, behavioral, and cognitive-behavioral modification programs; structured activity programs; sensory-stimulation therapies; multisensory approaches; music therapy; and mindfulness-based interventions) [224]. Meta-analyses of double-blind randomized controlled trials comparing antidepressants versus placebo for depression in AD revealed inefficacy in most cases with different drugs (sertraline, mirtazapine, imipramine, fluoxetine, and clomipramine) [225].

The mechanisms underlying depression in dementia still remain unclarified [226]. About 60% of depressive patients receive an inappropriate medication according to their pharmacogenetic background [66,227,228], and community psychiatrists and pharmacists are more accurate in their psychotropic prescriptions when they know the CYP profile of their patients [228,229,230].

Antidepressants are associated with the PGx activity of over 600 genes. The different pharmacological categories of antidepressants (nonselective monoamine reuptake inhibitors, selective serotonin reuptake inhibitors, nonselective monoamine oxidase (MAO) inhibitors, and other chemical modalities) are substrates, inhibitors, or inducers of 40, 22, and 9 enzyme/protein gene products, respectively, and are transported by 13 different protein transporters (Figure 1, Figure 2 and Figure 3 and Table 4). Enzymes of the Cytochrome P450 (CYP) family are involved in 100% of drugs approved for the treatment of depression. Antidepressants are major substrates of CYP2D6 (86%), CYP3A4 (72%), CYP2C19 (60%), CYP1A2 (57%), CYP2C9 (34%), UGT1A4 (29%), and UGT1A3 (25%); inhibitors of CYP2D6 (69%), CYP3A4 (55%), CYP1A2 (45%), CYP2C19 (45%), CYP2C9 (34%), SLC6A4 (32%), MAOA (29%), MAOB (29%), and ABCB1 (25%); and inducers of CYP3A4 (5%), CYP1A2 (5%), CYP2B6 (5%), CYP2C9 (3%), CYP2C19 (3%), CYP2D6 (3%), and ABCB1 (3%). Major transporters of antidepressants are SLC6A4 (62%), ABCB1 (55%), and SLC6A2 (40%) (Table 4). Sertraline is associated with 31 pharmagenes, Milnacipran is associated with 31, Mirtazapine is associated with 30, Fluoxetine is associated with 28, and Imipramine is associated with 28 [55,103]. The PGx of antidepressants has become quite well known over the past 20 years, with no relevant breakthroughs in the past decade [55,66].

*CYP2C19* and *CYP2D6* variants affect the occurrence of ADRs in patients treated with selective serotonin reuptake inhibitors (SSRIs) (citalopram, escitalopram, sertraline, fluvoxamine, fluoxetine, and paroxetine), including anxiety, nightmares, and panic attacks associated with CYP2D6 and electrocardiogram (ECG)-prolonged QT associated with CYP2C19 [231]. Prolonged QTc interval is prevalent in patients with moderate-severe dementia, with behavioral symptoms, with global and temporal atrophy, and with leukoaraiosis [232]. A pharmacogenetic risk score has been proposed with top-scoring SNPs (rs12248560, rs878567, and rs17710780). The *HTR1A rs878567* and *CYP2C19 rs12248560* variants showed association with depression severity [233]. *ACE* variants influence mood in AD [111]. The coadministration of ACE inhibitors and statins with antidepressants may affect therapeutic outcomes [234].

The influence of PGx factors on the pharmacogenetics and pharmacodynamics of many antidepressants has been reasonably well documented [55,66,235,236,237]; however, important aspects such as body weight gain, suicidality, cyclothymic reactions, cardiovascular risks, and potential neurotoxicity still remain obscure [55].

Lithium is the gold standard for the treatment of bipolar disorder (BPD) and BPD-like symptoms in a reduced number of cases with dementia. The response to lithium is very variable and depends on the PGx background of each patient [238]. Lithium PGx is very complex. Caution and personalized dose adjustment is highly recommended in patients with the following genotypes: *BCR* (Asn796Ser), *BDNF* (C/G (rs988748) and G/A (Val66Met)), *CACNG2* (rs2284017, rs2284018, and rs5750285), *GSK3B* (T-50C and GSK3-beta*C), *INPP1* (C973A and rs1882891), mtDNA (carriers of the 0398A polymorphism show a better response to lithium), *NTRK2* (rs1387923 and rs1565445), and variants in other genes (*ABCG2, CCND1, CREB1, DRD1, ESR1, FMR1, GRIK2, IMPA1, IMPA2, NR3C1, PTGES, SLC6A4,* and *VEGFA*) [55,56,238].

### 5.3. Anxiety Disorders

Anxiety-like disorders are present in 30–40% of cases with dementia during the disease process. Anxiety is a risk factor for dementia [239,240], and anxiety-like behaviors are persistent in patients with dementia. Early-onset AD patients exhibit greater prevalence of all BDs, especially anxiety, irritability, and sleep disorders [241].

The neurobiology of anxiety in dementia is unknown. Subjective cognitive decline (SCD) (self-reported cognitive deficits), without measurable cognitive impairment, has been associated with brain structural alterations and *APOE-4*. SCD cases show decreased total cortical volumes and cortical surface area, which are especially prominent in *APOE-4* carriers. Anxiety symptoms are negatively associated with the right cortical surface area in *APOE-4* noncarriers with SCD [242]. Depression, anxiety, and cerebrovascular risk contribute to SCD [243].

Benzodiazepines are currently prescribed for ameliorating this symptomatology; however, benzodiazepines contribute to cognitive and psychomotor dysfunction [244].

### 5.4. Sleep Disorders

Chronic sleep disorders might represent a risk factor for dementia, and alterations in circadian rhythms and consequent sleep disorders are common in AD and age-related disorders [245,246,247,248]. The neurobiology of circadian rhythms in AD is not well documented. Different neurotransmitters influence circadian changes and sleep disorders (insomnia, parasomnias, circadian rhythm sleep-wake disorders, hypersomnolence, sleep-related movement disorders, and sleep-related breathing disorders), including melatonin, histamine, GABA, hypocretin, dopamine, noradrenaline, serotonin, and adenosine. Genomic alterations in these systems (e.g., pathogenic genes) may affect the efficacy and safety of anxiolytics and hypnotics used in the treatment of some of these disorders [55].

Over 100 genes have been associated with sleep disorders in AD [249]. In the triple transgenic AD mouse model (3× Tg-AD), there is an abnormal expression of *Per* genes in the suprachiasmatic nucleus (SCN) [250]. The glymphatic system might be an effective mechanism of brain Aβ-amyloid clearance particularly effective during sleep. Aquaporin-4 may play a role in glymphatic function, since ablation of Aquaporin-4 results in impairment of Aβ clearance mechanism and increased brain Aβ-amyloid deposition. The *AQP4* variant rs72878776 is associated with poorer overall sleep quality, and other SNPs might moderate the effect of sleep latency (rs491148, rs9951307, rs7135406, rs3875089, and rs151246) and duration (rs72878776, rs491148, and rs2339214) on brain Aβ-amyloid burden [251]. Homer1a and mGluR1/5 are implicated in sleep function to weaken synapses during sleep and to restore synapse homeostasis [252]. 

Evening secretion of melatonin is delayed and mildly impaired in patients with AD [253]. Rapid eye movement (REM) sleep influences memory consolidation. Noradrenaline participates in the regulation of REM sleep to maintain neuronal integrity and brain house-keeping functions [254].

Sleep dysfunction and Aβ deposition show synergistic effects to impair brain function. Brain Aβ deposition is associated with subjective measures of sleep quality and cognition. Nocturnal awakenings are associated with Aβ deposition in the precuneus and poor cognitive performance [255]. Extracellular levels of Aβ and tau show a fluctuating pattern during the normal sleep-wake cycle. Increased wakefulness and disturbed sleep lead to increased Aβ production and decreased Aβ clearance; additionally, chronic wakefulness increases Aβ aggregation and deposition, and Aβ accumulation results in disturbed sleep. Sleep deprivation increases brain and CSF tau levels and the spread of tau protein aggregates in neural tissues, correlating with decreased nonrapid eye movement (NREM) sleep slow wave activity [256,257].

Sleep disorders may precede cognitive impairment. Sleep disturbances alter periodic sleep architecture and electroencephalogram (EEG) patterns in prodromal stages [258]. Age-related cognitive impairment is associated with reduced delta, theta, and sigma power as well as spindle maximal amplitude during NREM sleep. Early sleep biomarkers of potential cognitive decline are poor sleep consolidation, lower amplitude, and faster frequency of spindles [259]. Decreased nonrapid eye movement (NREM) sleep slow wave activity associates with Aβ deposition and tauopathy. Aβ decreases nonrapid eye movement sleep and increases wakefulness. Aβ upregulates the expression levels of tau, pTau, orexin A, and adenosine A1 receptor [260]. Orexin receptor antagonists (e.g., Suvorexant) have been proposed as potential candidates for the treatment of sleep disorders and BDs in AD [261]. 

Anxiolytics, hypnotics, and sedatives are associated with the PGx activity of 445 genes. Different categories of anxiolytics (benzodiazepines, diphenylmethane derivatives, carbamates, dibenzo-bicyclo-octadiene derivatives, and azaspirodecanodiones), hypnotics, and sedatives (barbiturates, aldehydes, benzodiazepines, piperidinediones, melatonin receptor agonists, and other chemicals, alone or in combination) are substrates, inhibitors, or inducers of 47, 18, and 30 enzyme/protein gene products, respectively, and are transported by at least 30 protein transporters (Figure 2 and Figure 3 and Table 5). CYP enzymes participate in the metabolism of over 92% of drugs of these pharmacological categories. About 70% of drugs currently used for the treatment of anxiety, panic attacks, sleep disorders, agitation, and behavioral anomalies are major substrates of CYP3A4, followed by CYP2C19 (41%); CYP3A5 (38%); CYP2D6 (36%); CYP2C9 (30%); CYP1A2 (27%); CYP2B6 (19%); UGT1A4 (14%); UGT2B15 (11%); and UGT1A1, UGT1A3, UGT1A6, UGT1A10, and UGT2B7 (8%); only 10% are inhibitors of CYP3A4 and CYP2C9; 8% are inducers of CYP3A4, and about 5% are inducers of CYP1A2, CYP2A6, CYP7A1, and ABCC2. Over 50% of these drugs are transported by proteins of the CLCN family, 16% are transported by ABCB1, 9% are transported by NQ1I2, and 5% are transported by ABCC2, KCNE1, KCNH2, and SLCO1B1. Phenobarbital is associated with 80 pharmagenes, Midazolam is associated with 24, Temazepam is associated with 20, Diazepam is associated with 23, and Alprazolam is associated with 14 [55,103]. 

### 5.5. Epilepsy

Epilepsy is a prevalent disorder in dementia with prevalence and incidence rates 2–6-fold higher than in age-matched healthy subjects. Subclinical epileptiform activity can lead to accelerated cognitive decline. Aβ deposition may influence the propagation of synchronized abnormal discharges via excitatory pathways [262].

There is a complex epileptogenesis-associated dysregulation of proteins involved in amyloid β processing and regulation in the hippocampus (HC) and parahippocampal cortex during epileptogenesis, in which there is also involvement of tau and proteins of the mitochondrial complexes I, III, IV, and V [263]. Seizures are more prevalent in early-onset AD with rapid progression [264], correlating with high CSF total tau protein levels [265]. A disintegrin and metalloproteinase domain-containing protein 10 (ADAM10), which is an α-secretase in APP processing, has been associated with epilepsy as a stage-dependent modulator of epileptogenesis [266].

Potential pathogenic genes for epilepsy include ion channel genes (e.g., *SCN1A, KCNQ2, SCN2A,* and *SCN8A*), which account for nearly half of epilepsy genes, together with a number of additional genes, such as *CDKL5, STXBP1, PCDH19, PRRT2, LGI1, ALDH7A1, MECP2, EPM2A, ARX*, and *SLC2A1* [267]. There is also an association of *MTHFR rs1801133* and *ABCC2 rs717620* with susceptibility to generalized tonic-clonic epilepsy, while *ABCB1 rs717620* is associated with poor response to antiepileptics [268]. The influence of some of these genes in PGx has been investigated under different therapeutic paradigms [55]. 

Treatment of seizures in AD with low-dose antiepileptic drugs (AEDs) is usually well tolerated and efficacious, and selected AEDs might also help in slowing-down disease progression [269,270]. Anticonvulsants may suppress seizures in up to two-thirds of all patients, with no apparent effects on long-term prognosis [271]. 

Antiepileptics are associated with the PGx activity of approximately 150 genes (Table 6). The *GABRG2* gene encodes one of the subunits of the GABA-A receptor, the most abundant receptor of fast synaptic inhibition in the brain. *GABRAG2* variants (rs211037, rs210987, rs440218, rs2422106, rs211014, and rs401750) and several *PRRT2* mutations (c.649delC (p.R217Efs*12), c.649_650insC (p.R217Pfs*8), c.412C>G (p.Pro138Ala), c.439G>C (p.Asp147His), and *c.623C>A (p.Ser208Tyr*)) are associated with febrile seizures [272]. There is a clear association of rs211037 with epilepsy in Asian patients and of rs211037-rs210987 and rs2422106-rs211014-rs401750 haplotypes with susceptibility to symptomatic epilepsy in Chinese [273].

Different categories of anticonvulsants (barbiturates, hydantoin derivatives, oxazolidines, succinimides, benzodiazepines, carboxamides, fatty acid derivatives, and miscellaneous antiepileptics) are substrates, inhibitors, or inducers of 40, 15, and 24 enzyme/protein gene products, respectively, and are transported by at least 16 protein transporters (Table 6). CYP enzymes participate in the metabolism of over 69% of drugs of these pharmacological categories. Over 65% of drugs currently used for the treatment of epilepsy and related disorders are major substrates of CYP3A4, followed by CYP3A5, CYP2E1, and UGT1A1 (32%); CYP2C8 and CYP2B6 (26%); CYP1A2, CYP2C9, CYP2C19, UGT1A3, UGT1A9, and UGT2B7 (22%); CYP1A1, CYP1A6, CYP2D6, CYP3A7, UGT1A4, and UGT1A6 (16%); and CYP2C18, CYP4B1, UGT1A10, and UGT2B15 (11%). These drugs tend to be strong inhibitors of CYP2C9 (>25%) and, to a lesser extent, of CYP1A6 and SULT1A1 (10%), and they are also potent inducers of CYP3A4 (37%). ABCB1 is the major transporter of 42% of antiepileptics, followed by ABCC2 (16%), SLC6A1 (11%), and SLCO2A1 and SLCO1B1 (10%). Phenobarbital, Valproic acid, and Carbamazepine are the three best-characterized drugs for their pharmacogenomic profile, with over 100 genes involved in their biotransformation and metabolism [55,103,274,275].

*UGT2B7 G211T* and *C161T* polymorphisms affect the pharmacokinetics and pharmacodynamics of valproic acid (VPA) [276]. Carriers of the variant *UGT1A6 19T>G, 541A>G*, and *552A>C* allele require higher VPA dosages than noncarriers, and carriers of the variant *GRIN2B -200T>G* allele are more likely to require lower VPA dosages than noncarriers [277]. VPA-related liver damage has been associated with the formation of a hepatotoxic 4-ene metabolite mediated by CYP2C9 and CYP2A6 enzymes [278]. *ABAT rs1731017, SCN2A rs2304016*, and *ALDH5A1 rs1054899* are associated with VPA response in Chinese patients [279]. Female CYP2C19-PMs are more susceptible to VPA-induced weight gain in the Japanese population [280], and SNPs in the leptin receptor (*LEPR*) (rs1137101) and ankyrin repeat kinase domain containing 1 (*ANKK1*) (rs1800497) show associations with VPA-induced weight gain in the Chinese population [281]. Oral clearance (CL/F) of VPA in patients with the *LEPR-A668G* and *G668G (rs1137101)* variants is lower than in patients with the *LEPR-A668A* genotype [282]. Meropenem decreases VPA plasma levels when coadministered together. This interaction is triggered by inhibition of acylpeptide hydrolase (APEH) activity with meropenem. The study of VPA-d6 β-D-glucuronide (VPA-G) concentration in *APEH rs3816877* and *rs1131095* carriers revealed that patients with the *APEH rs3816877 C/C* genotype show higher levels than *C/T* carriers in the Chinese population [283]. Valproate-related neuroprotection in experimentally triggered epileptic seizures has been associated with PKC-dependent GABAAR γ2 phosphorylation at serine 327 residue [284]. *SOD2 Val16Ala* polymorphism may affect γ-glutamyltransferase (GGT) elevation in epileptic patients treated with VPA [285].

High initial serum concentrations of lamotrigine increase the risk of cutaneous ADRs. Genetic variants of uridine diphosphate glucuronosyltransferase (UGT) 1A4 influence lamotrigine elimination. UGT1A4*2 (P24T) and *3 (L48V) variants are associated with skin reactions but probably not in Caucasians [286]. *UGT2B7-161C>T* variants influence lamotrigine pharmacokinetics; specifically, the *UGT2B7-161TT* genotype changes lamotrigine clearance and may be useful in titrating the optimal lamotrigine dose [287]. *OCT1 rs628031* and *ABCG2 rs2231142* affect lamotrigine metabolism, and SNPs in *ABCG2 rs2231142, rs3114020, HNF4α rs2071197*, and *ABCB1 rs1128503* are associated with lamotrigine CDR (concentration/dose normalized by body weight) in Chinese patients [288,289].

Treatment with carbamazepine, oxcarbazepine, or phenytoin is associated with delayed-hypersensitivity reactions (e.g., eosinophilia, Stevens–Johnson syndrome, and toxic epidermal necrolysis) [290]. The FDA-approved label for oxcarbazepine indicates a pharmacogenomic association with hypersensitivity reactions and the HLA antigen allele HLA-B*15:02. Oxcarbazepine has structural similarities with carbamazepine, and HLA-B*15:02 is a risk factor for both carbamazepine- and oxcarbazepine-induced severe cutaneous ADRs, especially in the Asian population [290,291]. HLA-B*15:02 is highly associated with carbamazepine-related Stevens–Johnson syndrome/toxic epidermal necrolysis cases as well as phenytoin-related cutaneous ADRs and, to a lesser extent, lamotrigine ADRs; in contrast, HLA-B*40:01 and HLA-B*58:01 carriers show a lower frequency of carbamazepine-related skin complications [292]. HLA-A*31:01 has also been reported to be a genetic marker for carbamazepine-induced ADRs in both Japanese and European populations [293,294]. *SCN1A rs3812718 A/G* and *rs2290732 A/G* polymorphisms influence carbamazepine tolerability, and *rs2298771 A/G* is associated with carbamazepine efficacy [295]. *ABCB1 rs1045642* and *UGT2B7 rs7439366* affect oxcarbazepine pharmacokinetics and pharmacodynamics in Han Chinese epileptic patients [296]. PXR*1B, HNF4a rs2071197, CYP1A2*1F, ABCC2 1249G>A, and *PRRT2 c.649dupC* influence the pharmacokinetics and pharmacodynamics of carbamazepine [297]. ABCB1 c.3435C>T, CYP3A4*1G, CYP3A5*3, POR*28, and *EPHX1 c.416A>G* and *c.128G>C* variants influence carbamazepine metabolism in Chinese patients [298]. rs776746 and rs15524 in *CYP3A5* affect carbamazepine metabolism, and rs2032582 and rs10234411 in *ABCB1* contribute to interindividual variation in carbamazepine and in carbamazepine-10,11-epoxide transport in epileptic patients treated with carbamazepine in combination with phenytoin or phenobarbital [298]. *SCN1A IVS5-91G>A, UGT2B7 c.802T>C, ABCC2 c.1249G>A*, and *EPHX1 c.337T>C* carriers require higher maintenance doses of oxcarbamazepine [299,300]. Carriers of the variant *SCN1A IVS5-91G>A* and *EPHX1 c.337T>C* allele require higher carbamazepine dosages than noncarriers, and genetic variants in the *SCN1A, EPHX1*, and *UGT2B7* genes interactively affect the concentration–dose ratio of carbamazepine [301].

*SCN1A, CYP2C9, CYP2C19*, and *ABCB1* variants affect phenytoin metabolism. *CYP2C9* and *CYP2C19* polymorphisms are associated with lower phenytoin maintenance dosage in Asian patients. CYP2C19*2/*2, CYP2C19*3/*3, CYP2C19*2/*3, and heterozygous CYP2C9*3 variants require lower phenytoin maintenance dosage [301,302]. Phenytoin may cause cutaneous ADRs with variable severity (maculopapular exanthema, eosinophilia, Stevens–Johnson syndrome, and toxic epidermal necrolysis). At least 16 SNPs in *CYP2C* genes at 10q23.33 may contribute to this adverse effect. CYP2C9*3 carriers show a clear propensity to phenytoin-related severe cutaneous ADRs [303]. There is an association between a rare variant in the complement factor H-related 4 (*CFHR4*) gene and phenytoin-induced maculopapular exanthema in Europeans [304].

*CACNA1G, CACNA1H, CACNA1I*, and *ABCB1* variants are associated with differential short-term seizure outcome in childhood absence epilepsy. In patients treated with ethosuximide, *CACNA1H rs61734410/P640L* and *CACNA1I rs3747178* are more prevalent among not-seizure-free patients, and in patients treated with lamotrigine, *ABCB1 rs2032582/S893A* is more frequent in not-seizure-free patients, whereas *CACNA1H rs2753326* and *rs2753325* are more common in seizure-free patients [305].

Resistant epilepsy is an important problem in over 20% of patients treated with antiepileptics [306]. Pharmaco-resistance is directly linked to dysfunctions in the pharmacoepigenetic machinery [61]. The *C3435T* variant of the *ABCB1* gene has been proposed as a crucial factor for drug resistance in epilepsy. *ABCB1-C3435C* carriers show a risk of pharmacoresistance in some studies, but this association has been questioned after further analyses [307]. The *ABCB1 G2677T T* (rs1128503) and *C3435T T* (rs1045642) alleles and the *TT, CTT*, and *TTT* haplotypes are associated with drug-resistant epilepsy in specific populations [308]. *ABCC2 rs717620 -24 CT+TT* genotypes and *ABCC2 rs3740066 (3972C>T) CT+TT* genotypes are overrepresented in epileptic patients resistant to antiepileptic drugs in the Chinese population, whereas *ABCC2 rs2273697 (1249G>A)* and *ABCB1 rs1045642 (3435C>T)* polymorphisms were not found to be associated with drug-resistant epilepsy in this population. The frequency of the haplotype *TGT (ABCC2 -24C>T/ABCC2 1249G>A/ABCC2 3972C>T)* in resistant patients is double that of responsive patients [309]. The *TAGAA* haplotype in *CACNA1A* accumulates in drug-resistant patients in the Chinese population [310]. Glucose type-1 transporter (GLUT1) deficiency syndrome, caused by mutations in the *SLC2A1* gene, exhibits pharmacoresistance to antiepileptics. Screening of *SLC2A1* pathogenic variants can predict drug response and optimization of antiepileptic drugs for the treatment of this health condition [311]. The *SCN1A IVS5-91G>A AA* and *ABCC2 c.1249G>A GA* genotypes have been shown to be associated with carbamazepine/oxcarbamazepine-resistant epilepsy in the Chinese Han population. The frequency of *SCN1A-AA* and *ABCC2-AC* haplotypes is higher in drug-resistant patients than in responsive patients [312]. Association of *ABCC2 rs2273697* and *rs3740066* polymorphisms and drug-resistant epilepsy has been reported in Asia Pacific epilepsy cohorts [313]. *PCDH19* mutations may cause pharmacoresistant epilepsy and intellectual disability in Dravet-like syndromes. A retrospective study of antiepileptic therapy in females with *PCDH19* mutations showed that the most effective drugs in these cases are clobazam and bromide, with responder rates of 68% and 67%, respectively [314]. Clobazam binds to stereospecific receptors on the postsynaptic GABA neuron at several sites within the CNS (limbic system and reticular formation) and enhances the inhibitory effect of GABA on neuronal excitability by increasing neuronal membrane permeability to chloride ions, which results in hyperpolarization and stabilization. Clobazam is extensively metabolized by CYP enzymes. Its major metabolite is N-desmethylclobazam (norclobazam), which also display antiepileptic activity. Clobazam is a major substrate of CYP2C19 and CYP3A4 and a minor substrate of CYP2B6 and CYP2C18. CYP2C19-PMs show higher plasma levels of clobazam and increased risk of ADRs. Caution and personalized dose adjustment is recommended in patients with the following genotypes: CYP2C19 (CYP2C19*2, CYP2C19*3, CYP2C19*4, CYP2C19*5, CYP2C19*6, CYP2C19*7, CYP2C19*8, CYP2C19*17), CYP3A4 (CYP3A4*1, CYP3A4*1B, CYP3A4*2, CYP3A4*3, CYP3A4*4, CYP3A4*5, CYP3A4*6, CYP3A4*8, CYP3A4*11, CYP3A4*12, CYP3A4*13, CYP3A4*15, CYP3A4*17, CYP3A4*18, CYP3A4*19), and CYP3A5 (CYP3A5*3) [55,56] (Table 6).

In Japanese patients with Dravet syndrome, it seems that *CYP2C19* variants may influence a positive response to the antiepileptic effects of stiripentol [315]. Genotype combinations of *GABRA1 rs6883877, GABRA2 rs511310*, and *GABRA3 rs4828696* may affect responses to antiepileptic drugs [316]. ɣ-aminobutyric-acid (GABA) is the principal inhibitory neurotransmitter in the CNS. Imbalances in GABAergic neurotransmission are involved in the pathophysiology of epilepsy and AD. GABA transporters (GATs) regulate the influx-efflux of GABA with sodium and chloride at the synaptic cleft. GATs belong to the solute carrier 6 (SLC6) transporter family: GAT1-3 (SLC6A1, SLC6A13, and SLC6A11) and betaine/GABA transporter 1 (BGT1 and SLC6A12). BGT1 is a potential target for the treatment of epilepsy. The GAT1/BGT1 selective inhibitor EF1502 and the BGT1 selective inhibitor RPC-425 display anticonvulsant effects [317].

## 6. Conclusions

Symptomatic interventions for patients with dementia involve anti-dementia drugs to improve cognition, psychotropic drugs for the treatment of behavioral disorders (BDs), and different categories of drugs for concomitant disorders. Demented patients may take >6–10 drugs/day with the consequent risk for drug–drug interactions (DDIs) and adverse drug reactions (ADRs > 80%) which accelerate cognitive decline. PGx intervention may prevent ADRs and DDIs. The pharmacoepigenetic machinery is integrated by pathogenic, mechanistic, metabolic, transporter, and pleiotropic genes redundantly and promiscuously regulated by epigenetic mechanisms (DNA methylation, chromatin remodeling/histone changes, and miRNAs). *CYP2D6, CYP2C9, CYP2C19*, and *CYP3A4/5* geno-phenotypes are involved in the metabolism of over 90% of drugs currently used in patients with dementia, and only 20% of the population is an extensive metabolizer for this tetragenic cluster. ADRs associated with anti-dementia drugs, antipsychotics, antidepressants, anxiolytics, hypnotics, sedatives, and antiepileptic drugs can be minimized by means of pharmacogenetic screening prior to treatment. These drugs are substrates, inhibitors, or inducers of 58, 37, and 42 enzyme/protein gene products, respectively, and are transported by 40 different protein transporters. APOE is the reference gene in most pharmacogenetic studies. In multifactorial treatments, APOE-3 carriers are the best responders and APOE-4 carriers are the worst responders; likewise, CYP2D6-EMs are the best responders and CYP2D6-PMs are the worst responders. ACHE-BCHE variants also affect the pharmacogenetic outcome as well as some genes encoding components of the epigenetic machinery. 

Antipsychotics show limited efficacy in aggressive behaviors and psychotic symptoms and may increase mortality and risk of psychomotor disorders and cerebrovascular events. Major ADRs with antipsychotics are extrapyramidal symptoms, tardive dyskinesia, weight gain, hyperprolactinemia, and agranulocytosis. Antipsychotics are associated with the activity of ≈100 pharmagenes; antidepressants are associated with 600; and anxiolytics, hypnotics, and sedatives are associated with 445. CYP enzymes are involved in 100% of drugs approved for the treatment of depression, and about 60% of depressive patients are receiving an inappropriate medication according to their pharmacogenetic background. Carbamazepine, oxcarbazepine, or phenytoin may cause delayed-hypersensitivity reactions associated with the HLA antigen allele HLA-B*15:02. 

The incorporation of pharmacogenomic strategies for a personalized treatment in dementia is an effective option to optimize limited therapeutic resources and to reduce unwanted side-effects.

## 7. Further Considerations

Drug efficacy and safety are fundamental issues in dementia due to the complexity of the disorder and comorbidities which require polypharmacy [43]. Over 50% of patients over 60 years of age suffering chronic CNS disorders currently take 6–12 drugs/day with a high risk of drug toxicity, ADRs, and DDIs [43,56]. Over 20% of patients with depression develop dementia in a period of approximately 10 years. The chronic use of anticholinergic antidepressants might contribute to this pathogenic transformation [318]. It is also well-known that over 60% of patients chronically treated with antipsychotics develop extrapyramidal symptoms which may induce severe motor disability [319]. Over 80% of nursing home residents are daily consumers of psychotropic drugs [320,321] which are prescribed in excessive doses, for excessive duration, and without adequate monitoring and/or indications for their use [322]. Prescribing errors (≈50%) are common in patients treated with anti-dementia drugs [42], and potentially inappropriate prescribing (PIP) occurs in almost 80% of patients with dementia [323]. 

With appropriate PGx intervention, the frequency and intensity of PIP and ADRs may be reduced in approximately 50% of the cases. AD patients show diverse age-related comorbidities which require polypharmacy. The implementation of PGx procedures may help to minimize drug–drug interactions. For instance, *CYP2C19* variants influence the effects of proton-pump inhibitors and the onset of infections [324]. *ACE* variants affect the pharmacokinetics and pharmacodynamics of ACE inhibitors which may interact with psychotropic drugs, contributing to cerebrovascular dysfunction [229]. Another important issue is the use of anticoagulants in patients with dementia [103]. Different antithrombotic drugs (Acenocoumarol, Acetylsalicylic acid, Argatroban, Bivalirudin, Cilostazol, Clopidogrel, Dabigatran, Rivaroxaban, Dipyridamole, Lepirudin, Prasugel, Ticagrelor, Ticlopidine, and Warfarin) can be used in patients with atrial fibrillation, thrombophlebitis, and thromboembolic or ischemic stroke. Warfarin is one of the most common anticoagulants due to its low cost; however, its narrow therapeutic window makes it a candidate to a few ADRs in patients with dementia. Warfarin prolongs prothrombin time (PT) and activated partial thromboplastin time (APTT), and phytonadione (vitamin K1) reverses its anticoagulant effect. Warfarin is a substrate of CALU, CYP1A2, CYP2C8, CYP2C9, CYP2C18, CYP2C19, CYP3A4/5, CYP4F2, EPHX1, and GGCX; an inhibitor of CYP2C9, CYP2C19, and VKORC1; and an inducer of CYP2C9; and is transported by ABCB1. *CYP2C9* and *VKORC1* variants are determinant in warfarin efficacy and safety [55]. *VKORC1* variants and CYP2C9*3 are clearly associated with warfarin maintenance dosages. *CYP2C9* and *VKORC1* genotypes help to identify normal responders (60%), sensitive responders (35%), and highly sensitive responders (3%) to warfarin and characterized patients who can benefit from edoxaban compared with warfarin [325]. *VKORC1-AG* and *GG* carriers need higher doses than patients with *AA* genotypes, and CYP2C9*1/*3 carriers need doses lower than patients with the CYP2C9*1/*1 wild genotype [326]. In the Chinese region of Xinjiang, patients with atrial fibrillation carrying the *CT* and *TT* genotypes in the *GGCX* gene rs259251 loci need higher warfarin doses than *GGCX-CC* carriers [327].

*ADRB1 Ser49Gly* and *Arg389Gly* variants are associated with cardiovascular and β-blocker response outcomes. In patients with previous history of stroke, the *ADRB1 Gly49* polymorphism is associated with cardiovascular and cerebrovascular ADRs among β-blocker users [328].

In patients with minor ischemic stroke, platelet receptor gene (*P2Y12* and *P2Y1*) and glycoprotein gene (*GPIIIa*) polymorphisms influence antiplatelet drug responsiveness and clinical outcomes [329], and there are genetic differences (rs12143842) in the response of stroke patients to antihypertensive drugs (chlorthalidone, amlodipine, or lisinopril), especially regarding *HNRNPA1P4* and *NOS1AP* variants in African Americans and *PRICKLE1* and *NINJ2* variants in non-Hispanic Whites [330].

Rivaroxaban is currently used in thromboprophylaxis. *ABCB1 rs1045642*, *ABCB1 rs4148738*, *CYP3A4 rs35599367*, and *CYP3A5 rs776746* variants may influence rivaroxaban pharmacokinetics and prothrombin time dynamics [331].

The platelet-aggregation inhibitor Clopidogrel may also cause frequent complications. This antithrombotic agent is a substrate of CYP1A2, CYP2B6, CYP2C9, CYP2C19, and CYP3A4/5; and an inhibitor of CYP2B6, CYP2C9, and CYP2C19; and is transported by ABCB1. CYP2C19*2 (G681A and rs4244285), CYP2C19*3 (G363A and rs4986893), CYP2C19*17 (C806T and *rs12248560)*, and *ABCB1 (C3435T* and *rs1045642*) carriers with ischemic stroke are particularly sensitive to clopidogrel ADRs [55]. Clopidogrel resistance is frequent in patients with chronic kidney disease. Kidney dysfunction alters the association between *CYP2C19* variants and clopidogrel effects in patients with stroke or transient ischemic attack. Carriers of *CYP2C19* loss-of-function alleles (CYP2C19*2 and *3) have higher odds of new stroke than noncarriers [332,333] and increased risk of thromboembolic complications following neurointerventional procedures [334]. The AKR1D1*36 (rs1872930) allele is associated with the risk of major adverse cardiovascular and cerebrovascular events in clopidogrel-treated patients [335]. About 16–50% of patients treated with clopidogrel show platelet reactivity and an increased risk of ischemic events. Some methylated CpG sites have been associated with increased stroke recurrence. Lower cg03548645 (*TRAF3*) DNA methylation correlates with increased platelet aggregation in patients with stroke [336].

Studies on the PGx of anticoagulants for stroke prevention in patients with atrial fibrillation are not conclusive and deserve further investigation to optimize this currently used pharmacological intervention with still unclear results [337].

Orthopedic surgery is relatively frequent in patients with NDDs who experience perioperative neurocognitive disorders (delirium and postoperative cognitive dysfunction) which increase mortality [338]. PGx assessment is highly recommended for selection of appropriate anesthetics and postsurgical treatments in order to reduce postoperatory BDs and accelerated cognitive deterioration [55]. 

Opioid use disorder (OUD) is infrequent in AD; however, in AD cases treated with opioids, it is important to take into account that patients with at least one copy of the CYP3A4*1B allele exhibit an accelerated rate of metabolism compared to the wild-type allele CYP3A4*1 [339] and that CYP2D6-UMs show a better response to opiates than EMs, IMs, and PMs [55].

Several essential elements and metals (zinc, aluminum, copper, and cadmium), environmental toxicants, air pollutants (e.g., nanoparticles, particulate matter, ozone, and traffic-related air pollution), inappropriate medications, and drugs of abuse (amphetamines, cannabis, cocaine, and heroin) may contribute to neurotoxicity and may interfere with conventional treatments in dementia. Over 30% of patients with prodromal dementia are current users of anticholinergic drugs [340]. Chronic exposure to these products alters behavior and deteriorates cognition and psychomotor function in a dose-dependent fashion [341,342,343]. The pharmacogenetic genotyping of detoxification systems may help to predict risks in susceptible subjects [55].

It is also most important to elucidate the mechanisms underlying the drug-resistance phenomenon, which occurs in over 40% of cases with NPDs and in over 70% of cases with neoplastic processes [61,71]. Globally, over 20% of patients are resistant to conventional drugs [344,345] and it is estimated that pharmacogenetic and pharmacoepigenetic factors are important contributors to drug resistance [61,346,347,348].

Important issues to take into account in the coming years for the appropriate management of dementia are the identification of presymptomatic biomarkers and the discovery of effective drugs. Predictive markers associated with pathogenic genes for the presymptomatic diagnosis of dementia should incorporate both genomic and epigenetic signatures. Concerning the development of novel anti-pathogenic drugs, actual facts show that most CNS drugs are repressive rather than neuroprotective based on the regulation of a restrictive number of neurotransmitters; however, brain function depends upon the interplay of thousands of neuronal-glial factors pending full characterization. The discovery of novel anti-pathogenic drugs with neuroprotective properties is urgently needed to efficiently treat the different forms of brain dysfunction in dementia. The development of new drugs and clinically validated methods of identifying patients for a specific treatment should rely on PGx strategies.

## Figures and Tables

**Table 1:** Prevalent Alzheimer’s disease-related pathogenic genes.

Gene Symbol	Gene Name	Gene ID	OMIM #	Locus	dbSNP ID	Risk Allele	MAF
*ABCA7*	ATP binding cassette subfamily A member	10347	605414	19p13.3	rs3764650	G	0.20 (G)
*APOE*	Apolipoprotein E	348	107741	19q13.32	rs429358; rs7412	*4	0.15 (C); 0.08 (T)
*APP*	Amyloid beta precursor protein	351	104760	21q21.3	52 SNPs		<0.01
*BIN1*	Bridging integrator 1	274	601248	2q14.3	rs744373	C	0.36 (C)
*BUB3*	BUB3, mitotic checkpoint protein	9184	603719	10q26.13	rs4980270	T	0.10 (T)
*C9ORF72*	Chromosome 9 open reading frame 72	203228	614260	9p21.2	rs3849942	T	0.22 (T)
*CD2AP*	CD2 associated protein	23607	604241	6p12.3	rs9349407	C	0.25 (C)
*CD33*	CD33 molecule	945	159590	19q13.41	rs3865444	T	0.01 (T)
*CLU*	Clusterin	1191	185430	8p21.1	rs11136000	T	0.38 (T)
*CPZ*	Carboxypeptidase Z	8532	603105	4p16.1	rs7436874	C	0.36 (C)
*CR1*	Complement C3b/C4b receptor 1	185430	120620	1q32.2	rs3818361	T	0.25 (T)
*DISC1*	Disrupted in schizophrenia 1	27185	605210	1q42.2	rs16856202	G	0.03 (G)
*ENPP1*	Ectonucleotide pyrophosphatase/phosphodiesterase 1	5167	173335	6q23.2	rs7767170	T	0.02 (T)
*EXO1*	Exonuclease 1	9156	606063	1q43	rs1776148	A	0.27 (A)
*LAMA3*	Laminin subunit alpha 3	64231	606548	11q12.2	rs11082762	A	0.47 (A)
*LHFP*	Lipoma HMGIC fusion partner	10186	606710	13q13.3-q14.11	rs7995844	G	0.35 (G)
*MAPT*	Microtubule associated protein tau	4137	157140	17q21.31	15 SNPs		<0.01
*MS4A4E*	Membrane spanning 4-domains A4E	643680	608401	8p21.1	rs670139	A	0.38 (A)
*MS4A6A*	Membrane spanning 4-domains A6A	64231	606548	11q12.2	rs610932	A	0.45 (A)
*NLRP4*	NLR family pyrin domain containing 4	147945	609645	19q13.43	rs12462372	A	0.08 (A)
*NTNG1*	Netrin G1	3909	600805	18q11.2	rs11803905	T	0.32 (T)
*PICALM*	Phosphatidylinositol binding clathrin assembly protein	8301	603025	11q14.2	rs3851179	A	0.31 (A)
*PIWIL2*	Piwi like RNA-mediated gene silencing 2	55124	610312	8p21.3	rs4266653	G	0.47 (G)
*PSEN1*	Presenilin 1	5663	104311	14q24.2	241 SNPs		< 0.01
*PSEN2*	Presenilin 2	5664	600759	1q42.13	43 SNPs		< 0.01
*STK36*	Serine/threonine kinase 36	27148	607652	2q35	rs2303565	C	0.33 (C)
*STX17*	Syntaxin 17	55014	604204	9q31.1	rs1997368	G	0.32 (G)
*SUN3*	Sad1 and UNC84 domain containing 3	256979	607723	7p12.3	rs2708909	G	0.39 (G)
*TBC1D5*	TBC1 domain family member 5	9779	615740	3p24.3	rs10510480	C	0.11 (C)
*USP6NL*	USP6 N-terminal like	9712	605405	10p14	rs3847437	T	0.04 (T)
*ZSWIM7*	Zinc finger SWIM-type containing 7	125150	614535	17p12	rs10491104	T	0.41 (T)

**Table 2:** Pharmacogenetics of conventional anti-dementia drugs.

Drug	Properties	Pharmacogenetics
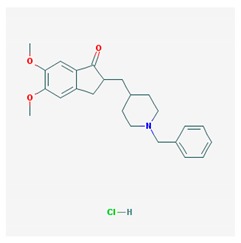	**Name: Donepezil hydrochloride,** Aricept, 120011-70-3, Donepezil HCl, BNAG, E-2020, E2020	**Pathogenic genes:***APOE*, *CHAT* **Mechanistic genes:***CHAT*, *ACHE*, *BCHE* **Metabolic genes:** ** Substrate:***CYP2D6* (major), *CYP3A4* (major), *UGTs*, *ACHE* ** Inhibitor:** *ACHE*, *BCHE* **Transporter genes:** *ABCB1*
	**IUPAC Name:** 2-[(1-benzylpiperidin-4-yl)methyl]-5,6-dimethoxy-2,3-dihydroinden-1-one;hydrochloride	
	**Molecular Formula:** C_24_H_30_ClNO_3_	
	**Molecular Weight:** 415.9529 g/mol	
	**Category:** Cholinesterase inhibitor	
	**Mechanism:** Centrally active, reversible acetylcholinesterase inhibitor; increases the acetylcholine available for synaptic transmission in the CNS	
	**Effect:** Nootropic agent, cholinesterase inhibitor, parasympathomimetic effect	
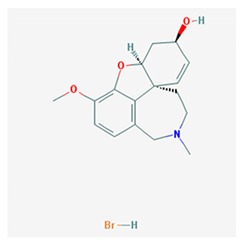	**Name: Galantamine hydrobromide**	**Pathogenic genes:***APOE, APP* **Mechanistic genes:***ACHE, BCHE, CHRNA4, CHRNA7, CHRNB2* **Metabollic genes:** ** Substrate:***CYP2D6* (major), *CYP3A4* (major), *UGT1A1* ** Inhibitor:** *ACHE, BCHE*
	**IUPAC Name:** (1S,12S,14R)-9-methoxy-4-methyl-11-oxa-4-azatetracyclo[8.6.1.0^{1,12}.0^{6,17}]heptadeca-6,8,10(17),15-tetraen-14-ol	
	**Molecular Formula:** C_17_H_22_BrNO_3_	
	**Molecular Weight:** 368.26548 g/mol	
	**Category:** Cholinesterase inhibitor	
	**Mechanism:** Reversible and competitive acetylcholinesterase inhibition leading to an increased concentration of acetylcholine at cholinergic synapses; modulates nicotinic acetylcholine receptor; may increase glutamate and serotonin levels	
	**Effect:** Nootropic agent, cholinesterase inhibitor, parasympathomimetic effect	
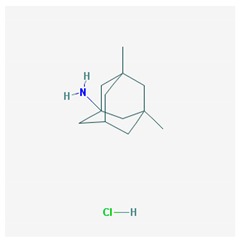	**Name: Memantine Hydrochloride**, 41100-52-1, Namenda, Memantine HCL, Axura, 3,5-Dimethyl-1-adamantanamine hydrochloride, 3,5-dimethyladamantan-1-amine hydrochloride	**Pathogenic genes:***APOE, MAPT, PSEN1* **Mechanistic genes:***CHRFAM7A, DLGAP1, FOS, GRIN2A, GRIN2B, GRIN3A, HOMER1, HTR3A* **Metabolic genes:** ** Inhibitor***:CYP1A2* (weak), *CYP2A6* (weak), *CYP2B6* (strong), *CYP2C9* (weak), *CYP2C19* (weak), *CYP2D6* (strong), *CYP2E1* (weak), *CYP3A4* (weak), *NR1I2* **Transporter genes:** *NR1I2*; **Pleiotropic genes:** *APOE, MAPT, MT-TK, PSEN1*
	**IUPAC Name:** 3,5-dimethyladamantan-1-amine;hydrochloride	
	**Molecular Formula:** C_12_H_22_ClN	
	**Molecular Weight:** 215.76278 g/mol	
	**Category:** N-Methyl-D-Aspartate receptor antagonist	
	**Mechanism:** Binds preferentially to NMDA receptor-operated cation channels; may act by blocking actions of glutamate, mediated in part by NMDA receptors	
	**Effect:** Dopamine agent, antiparkinson agent, excitatory amino acid antagonist, antidyskinetic	
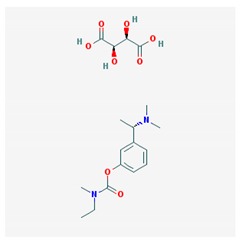	**Name: Rivastigmine tartrate**, 129101-54-8, SDZ-ENA 713, Rivastigmine hydrogentartrate, Rivastigmine Hydrogen Tartrate, ENA 713, ENA-713	**Pathogenic genes:** *APOE, APP, CHAT* **Mechanistic genes:** *ACHE, BCHE, CHAT, CHRNA4, CHRNB2* **Metabolic genes:** ** Inhibitor:** *ACHE, BCHE* **Pleiotropic genes:** *APOE, MAPT*
	**IUPAC Name:** (2R,3R)-2,3-dihydroxybutanedioic acid;[3-[(1S)-1-(dimethylamino)ethyl]phenyl] N-ethyl-N-methylcarbamate	
	**Molecular Formula:** C_18_H_28_N_2_O_8_	
	**Molecular Weight:** 400.42352 g/mol	
	**Category:** Cholinesterase inhibitor	
	**Mechanism:** Increases acetylcholine in CNS through reversible inhibition of its hydrolysis by cholinesterase	
	**Effect:** Neuroprotective agent, cholinesterase inhibitor, cholinergic agent	
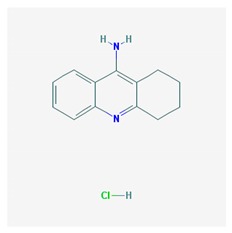	**Name: Tacrine Hydrochloride**, Tacrine HCl, 1684-40-8, Hydroaminacrine, tacrine.HCl, 9-AMINO-1,2,3,4-TETRAHYDROACRIDINE HYDROCHLORIDE, Tenakrin	**Pathogenic genes:** *APOE* **Mechanistic genes:** *ACHE, BCHE, CHRNA4, CHRNB2* **Metabolic genes:** ** Substrate:** *CYP1A2 (major), CYP2D6 (minor), CYP3A4 (major)* ** Inhibitor:** *ACHE, BCHE, CYP1A2 (weak)* **Transporter genes:** *SCN1A* **Pleiotropic genes:** *APOE, CES1, GSTM1, GSTT1, LEPR, MTHFR*
	**IUPAC Name:** 1,2,3,4-tetrahydroacridin-9-amine;hydrochloride	
	**Molecular Formula:** C_13_H_15_ClN_2_	
	**Molecular Weight:** 234.7246 g/mol	
	**Category:** Cholinesterase inhibitor	
	**Mechanism:** Elevates acetylcholine in cerebral cortex by slowing degradation of acetylcholine	
	**Effect:** Nootropic agent, cholinesterase inhibitor, Parasympathomimetic effect	

**AADAC**: arylacetamide deacetylase; **AANAT**: aralkylamine N-acetyltransferase; **ABAT:** 4-aminobutyrate aminotransferase; **ABCB1**: ATP-binding cassette, sub-family B (MDR/TAP), member 1; **ABCB11:** ATP-binding cassette, sub-family B (MDR/TAP), member 11; **ABCC1**: ATP-binding cassette, sub-family C (CFTR/MRP), member 1; **ABCC2:** ATP-binding cassette, sub-family C (CFTR/MRP), member 2; **ABCC3:** ATP-binding cassette, sub-family C (CFTR/MRP), member 3; **ABCC4:** ATP-binding cassette, sub-family C (CFTR/MRP), member 4; **ABCC6:** ATP-binding cassette, sub-family C (CFTR/MRP), member 6; **ABCC8:** ATP-binding cassette, sub-family C (CFTR/MRP), member 8; **ABCG2:** ATP-binding cassette, sub-family G (WHITE), member 2 (Junior blood group); **ACACA**: acetyl-CoA carboxylase alpha; **ACADSB:** acyl-CoA dehydrogenase short/branched chain; **ACHE:** acetylcholinesterase (Yt blood group); **ACSL1**: acyl-CoA synthetase long-chain family member 1; **ACSL3**: acyl-CoA synthetase long-chain family member 3; **ACSL4**: acyl-CoA synthetase long-chain family member 4; **ACSM1**: acyl-CoA synthetase medium-chain family member 1; **ACSM2B**: acyl-CoA synthetase medium-chain family member 2B; **ACSM3**: acyl-CoA synthetase medium-chain family, member 3; **ADCY1:** adenylate cyclase 1 (brain); **ADH1A**: alcohol dehydrogenase 1A (class I), alpha polypeptide; **ADH1B**: alcohol dehydrogenase 1B (class I), beta polypeptide; **ADH1C**: alcohol dehydrogenase 1C (class I), gamma polypeptide; **ADH4**: alcohol dehydrogenase 4 (class II), pi polypeptide; **ADH5**: alcohol dehydrogenase 5 (class III), chi polypeptide; **ADH6**: alcohol dehydrogenase 6 (class V); **ADH7**: alcohol dehydrogenase 7 (class IV), mu or sigma polypeptide; **ADHs**: alcohol dehydrogenases; **ADHFE1**: alcohol dehydrogenase, iron containing, 1; **ADIPOQ:** adiponectin, C1Q and collagen domain containing; **ADRA1A**: adrenoceptor alpha 1A; **ADRA1B**: adrenoceptor alpha 1B; **ADRA1D**: adrenoceptor alpha 1D; **ADRA1s**: alpha 1-adrenergic receptor family; **ADRA2A**: adrenoceptor alpha 2A; **ADRA2B**: adrenoceptor alpha 2B; **ADRA2C**: adrenoceptor alpha 2C; **ADRA2s**: alpha 2-adrenergic receptor family; **ADRAs**: alpha-adrenergic receptor family; **ADRB1**: adrenoceptor beta 1; **ADRB2**: adrenoceptor beta 2, Surface; **ADRB3**: adrenoceptor beta 3; **ADRBs**: beta-adrenergic receptor family; **ADRs:** adrenoceptors; **AGXT**: alanine-glyoxylate aminotransferase; **AHR**: aryl hydrocarbon receptor; **AKR1A1**: aldo-keto reductase family 1, member A1 (aldehyde reductase); **AKR1B1**: aldo-keto reductase family 1, member B1 (aldose reductase); **AKR1C1**: aldo-keto reductase family 1, member C1; **AKR1D1**: aldo-keto reductase family 1, member D1; **ALB:** albumin; **ALDH1A1**: aldehyde dehydrogenase 1 family, member A1; **ALDH1A2**: aldehyde dehydrogenase family 1, subfamily A2; **ALDH1A3**: aldehyde dehydrogenase family 1, subfamily A3; **ALDH1B1**: aldehyde dehydrogenase 1 family, member B1; **ALDH2**: aldehyde dehydrogenase 2 family (mitochondrial); **ALDH3A1**: aldehyde dehydrogenase 3 family, member A1; **ALDH3A2**: aldehyde dehydrogenase 3 family, member A2; **ALDH3B1**: aldehyde dehydrogenase 3 family, member B1; **ALDH3B2**: aldehyde dehydrogenase 3 family, member B2; **ALDH4A1**: aldehyde dehydrogenase 4 family, member A1; **ALDH5A1**: aldehyde dehydrogenase 5 family, member A1; **ALDH6A1**: aldehyde dehydrogenase 6 family, member A1; **ALDH7A1**: aldehyde dehydrogenase 7 family, member A1; **ALDH8A1**: aldehyde dehydrogenase 8 family, member A1; **ALDH9A1**: aldehyde dehydrogenase 9 family, member A1; **ANKK1**: ankyrin repeat and kinase domain containing 1; **AOX1**: aldehyde oxidase 1; **APOA1:** apolipoprotein A-I; **APOA5**: apolipoprotein A-V; **APOC3**: apolipoprotein C-III; **APOD**: apolipoprotein D; **APOE:** apolipoprotein E; **APP:** amyloid beta (A4) precursor protein; **AQP1:** aquaporin-1; **AS3MT**: arsenic (+3 oxidation state) methyltransferase; **ASMT**: acetylserotonin O-methyltransferase; **BAAT**: bile acid CoA: amino acid N-acyltransferase (glycine N-choloyltransferase); **BCHE:** butyrylcholinesterase; **BCL2**: B-cell CLL/lymphoma 2; **BCL2L1**: BCL2-like 1; **BDNF**: brain-derived neurotrophic factor; **BLK:** BLK proto-oncogene, Src family tyrosine kinase; **CA1:** carbonic anhydrase 1; **CA2:** carbonic anhydrase 2; **CA3:** carbonic anhydrase 3; **CA4:** carbonic anhydrase 4; **CA7:** carbonic anhydrase 7; **CA12:** carbonic anhydrase 12; **CA14:** carbonic anhydrase 14; **CACNA1C:** calcium channel, voltage-dependent, L type, alpha 1C subunit; **CACNs**: calcium channel, voltage-dependent family; **CALM1**: calmodulin 1 (phosphorylase kinase, delta); **CALMs**: calmodulins; **CALY:** Calcyon neuron specific vesicular protein; **CASR:** calcium-sensing receptor; **CAT:** catalase; **CBR1**: carbonyl reductase 1; **CBR3**: carbonyl reductase 3; **CBR4**: carbonyl reductase 4; **CBS:** cystathionine-beta-synthase; **CCBL1**: cysteine conjugate-beta lyase, cytoplasmic; **CCND1:** cyclin D1; **CDA**: cytidine deaminase; **CEL**: carboxyl ester lipase; **CELF4**: CUGBP, Elav-like family member 4; **CERKL**: ceramide kinase-like; **CES1**: carboxylesterase 1; **CES1P1**: carboxylesterase 1 pseudogene 1; **CES2**: carboxylesterase 2; **CES3**: carboxylesterase 3; **CES5A**: carboxylesterase 5A; **CFTR**: cystic fibrosis transmembrane conductance regulator (ATP-binding cassette sub-family C, member 7); **CHRM1**: cholinergic receptor, muscarinic 1; **CHRM2**: cholinergic receptor, muscarinic 2; **CHRM3**: cholinergic receptor, muscarinic 3; **CHRM4**: cholinergic receptor, muscarinic 4; **CHRM5**: cholinergic receptor, muscarinic 5; **CHRMs**: muscarinic cholinergic receptor family; **CHRNA2:** Cholinergic receptor nicotinic alpha 2 subunit; **CHRNA3:** Cholinergic receptor nicotinic alpha 3 subunit; **CHRNA4:** cholinergic receptor, nicotinic, alpha 4 (neuronal); **CHRNA7:** cholinergic receptor, nicotinic, alpha 7 (neuronal); **CHRNAs:** nicotinic cholinergic receptors, alpha type; **CHRNB2:** cholinergic receptor, nicotinic, beta 2 (neuronal); **CHRNB4:** cholinergic receptor nicotinic beta 4 subunit; **CHRNs**: nicotinic cholinergic receptor family; **CHST1**: carbohydrate (keratan sulfate Gal-6) sulfotransferase 1; **CHST2**: carbohydrate (N-acetylglucosamine-6-O) sulfotransferase 2; **CHST3**: carbohydrate (chondroitin 6) sulfotransferase 3; **CHST4**: carbohydrate (N-acetylglucosamine 6-O) sulfotransferase 4; **CHST5**: carbohydrate (N-acetylglucosamine 6-O) sulfotransferase 5; **CHST6**: carbohydrate (N-acetylglucosamine 6-O) sulfotransferase 6; **CHST7**: carbohydrate (N-acetylglucosamine 6-O) sulfotransferase 7; **CHST8**: carbohydrate (N-acetylgalactosamine 4-0) sulfotransferase 8; **CHST9**: carbohydrate (N-acetylgalactosamine 4-0) sulfotransferase 9; **CHST10**: carbohydrate sulfotransferase 10; **CHST11**: carbohydrate (chondroitin 4) sulfotransferase 11; **CHST12**: carbohydrate (chondroitin 4) sulfotransferase 12; **CHST13**: carbohydrate (chondroitin 4) sulfotransferase 13; **CKS1B:** CDC28 protein kinase regulatory subunit 1B; **CLCNs:** voltage-sensitive chloride channel family; **CNR1**: cannabinoid receptor 1 (brain); **CNTF**: ciliary neurotrophic factor; **COMT**: Catechol-O-methyltransferase; **CREB1:** cAMP responsive element binding protein 1; **CRHR1:** corticotropin releasing hormone receptor 1; **CRHR2:** corticotropin releasing hormone receptor 2; **CXCR2:** chemokine (C-X-C motif) receptor 2; **CYB5R3**: cytochrome b5 reductase 3; **CYP1A1**: cytochrome P450, family 1, subfamily A, polypeptide 1; **CYP1A2**: cytochrome P450, family 1, subfamily A, polypeptide 2; **CYP1B1**: cytochrome P450, family 1, subfamily B, polypeptide 1; **CYP2A6**: cytochrome P450, family 2, subfamily A, polypeptide 6; **CYP2A7**: cytochrome P450, family 2, subfamily A, polypeptide 7; **CYP2A13**: cytochrome P450, family 2, subfamily A, polypeptide 13; **CYP2B6**: cytochrome P450, family 2, subfamily B, polypeptide 6; **CYP2C8**: cytochrome P450, family 2, subfamily C, polypeptide 8; **CYP2C9**: cytochrome P450, family 2, subfamily C, polypeptide 9; **CYP2C18**: cytochrome P450, family 2, subfamily C, polypeptide 18; **CYP2C19**: cytochrome P450, family 2, subfamily C, polypeptide 19; **CYP2D6**: cytochrome P450, family 2, subfamily D, polypeptide 6; **CYP2D7P1**: cytochrome P450, family 2, subfamily D, polypeptide 7 pseudogene 1; **CYP2E1**: cytochrome P450, family 2, subfamily E, polypeptide 1; **CYP2F1**: cytochrome P450, family 2, subfamily F, polypeptide 1; **CYP2J2**: cytochrome P450, family 2, subfamily J, polypeptide 2; **CYP2R1**: cytochrome P450, family 2, subfamily R, polypeptide 1; **CYP2S1**: cytochrome P450, family 2, subfamily S, polypeptide 1; **CYP2W1**: cytochrome P450, family 2, subfamily W, polypeptide 1; **CYP3A4**: cytochrome P450, family 3, subfamily A, polypeptide 4; **CYP3A4/5:** cytochrome P450, family 3, subfamily A, polypeptide 4/5; **CYP3A5**: cytochrome P450, family 3, subfamily A, polypeptide 5; **CYP3A7**: cytochrome P450, family 3, subfamily A, polypeptide 7; **CYP3A43**: cytochrome P450, family 3, subfamily A, polypeptide 43; **CYP3As**: cytochrome P450, family 3, subfamily A; **CYP4A11**: cytochrome P450, family 4, subfamily A, polypeptide 11; **CYP4A22**: cytochrome P450, family 4, subfamily A, polypeptide 22; **CYP4B1**: cytochrome P450, family 4, subfamily B, polypeptide 1; **CYP4F2**: cytochrome P450, family 4, subfamily F, polypeptide 2; **CYP4F3**: cytochrome P450, family 4, subfamily F, polypeptide 3; **CYP4F8**: cytochrome P450, family 4, subfamily F, polypeptide 8; **CYP4F11**: cytochrome P450, family 4, subfamily F, polypeptide 11; **CYP4F12**: cytochrome P450, family 4, subfamily F, polypeptide 12; **CYP4Z1**: cytochrome P450, family 4, subfamily Z, polypeptide 1; **CYP7A1**: cytochrome P450, family 7, subfamily A, polypeptide 1; **CYP7B1**: cytochrome P450, family 7, subfamily B, polypeptide 1; **CYP8B1**: cytochrome P450, family 8, subfamily B, polypeptide 1; **CYP11A1**: cytochrome P450, family 11, subfamily A, polypeptide 1; **CYP11B1**: cytochrome P450, family 11, subfamily B, polypeptide 1: **CYP11B2**: cytochrome P450, family 11, subfamily B, polypeptide 2; **CYP17A1**: cytochrome P450, family 17, subfamily A, polypeptide 1; **CYP19A1**: cytochrome P450, family 19, subfamily A, polypeptide 1; **CYP20A1**: cytochrome P450, family 20, subfamily A, polypeptide 1; **CYP21A2**: cytochrome P450, family 21, subfamily A, polypeptide 2; **CYP24A1**: cytochrome P450, family 24, subfamily A, polypeptide 1; **CYP26A1**: cytochrome P450, family 26, subfamily A, polypeptide 1; **CYP26B1**: cytochrome P450, family 26, subfamily B, polypeptide 1; **CYP26C1**: cytochrome P450, family 26, subfamily C, polypeptide 1; **CYP27A1**: cytochrome P450, family 27, subfamily A, polypeptide 1; **CYP27B1**: cytochrome P450, family 27, subfamily B, polypeptide 1; **CYP39A1**: cytochrome P450, family 39, subfamily A, polypeptide 1; **CYP46A1**: cytochrome P450, family 46, subfamily A, polypeptide 1; **CYP51A1**: cytochrome P450, family 51, subfamily A, polypeptide 1; **DAO**: D-amino-acid oxidase; **DDC:** dopa decarboxylase (aromatic L-amino acid decarboxylase); **DDOST**: dolichyl-diphosphooligosaccharide--protein glycosyltransferase subunit (non-catalytic); **DHRS1**: dehydrogenase/reductase (SDR family) member 1; **DHRS2**: dehydrogenase/reductase (SDR family) member 2; **DHRS3**: dehydrogenase/reductase (SDR family) member 3; **DHRS4**: dehydrogenase/reductase (SDR family) member 4; **DHRS7**: dehydrogenase/reductase (SDR family) member 7; **DHRS9**: dehydrogenase/reductase (SDR family) member 9; **DHRS12**: dehydrogenase/reductase (SDR family) member 12; **DHRS13**: dehydrogenase/reductase (SDR family) member 13; **DHRSX**: dehydrogenase/reductase (SDR family) X-linked; **DIO2:** deiodinase, iodothyronine, type II; **DLGAP1**: discs, large (Drosophila) homolog-associated protein 1; **DPEP1**: dipeptidase 1 (renal); **DPP4:** dipeptidyl-peptidase 4; **DPYD**: dihydropyrimidine dehydrogenase; **DRD1**: dopamine receptor D1; **DRD2**: dopamine receptor D2; **DRD3**: dopamine receptor D3; **DRD4**: dopamine receptor D4; **DRD5**: dopamine receptor D5; **DRDs:** dopamine receptors; **DTNBP1**: dystrobrevin binding protein 1; **EPHX1**: Epoxide hydrolase 1, microsomal (xenobiotic); **EPHX2**: epoxide hydrolase 2, microsomal (xenobiotic); **ESD**: esterase D; **FABP1**: fatty acid binding protein 1, liver; **FGB:** fibrinogen beta chain; **FKBP5:** binding protein 5; **FMO1**: flavin containing monooxygenase 1; **FMO2**: flavin containing monooxygenase 2; **FMO3**: flavin containing monooxygenase 3; **FMO4**: flavin containing monooxygenase 4; **FMO5**: flavin containing monooxygenase 5; **FMO6P**: flavin containing monooxygenase 6 pseudogene; **FMOs**: flavin containing monooxygenases; **FOS:** FBJ murine osteosarcoma viral oncogene homolog; **GABBRs:** gamma-aminobutyric acid (GABA) A receptors, beta; **GABRA1:** gamma-aminobutyric acid (GABA) A receptor, alpha 1; **GABRA2:** gamma-aminobutyric acid (GABA) A receptor, alpha 2; **GABRA3:** gamma-aminobutyric acid (GABA) A receptor, alpha 3; **GABRA4:** gamma-aminobutyric acid (GABA) A receptor, alpha 4; **GABRA5:** gamma-aminobutyric acid (GABA) A receptor, alpha 5; **GABRA6:** gamma-aminobutyric acid (GABA) A receptor, alpha 6; **GABRAs:** gamma-aminobutyric acid (GABA) A receptors; **GABRB1:** gamma-aminobutyric acid type A receptor beta1 subunit; **GABRB2:** gamma-aminobutyric acid type A receptor beta2 subunit; **GABRB3:** gamma-aminobutyric acid (GABA) A receptor, beta 3; **GABRBs:** gamma-aminobutyric acid (GABA) A receptors, beta subtype; **GABRD:** gamma-aminobutyric acid (GABA) A receptor, delta; **GABRE:** gamma-aminobutyric acid (GABA) A receptor, épsilon; **GABRG1:** gamma-aminobutyric acid type A receptor gamma1 subunit; **GABRG2:** gamma-aminobutyric acid (GABA) A receptor, gamma 2; **GABRG3:** gamma-aminobutyric acid type A receptor pi subunit; **GABRGs:** gamma-aminobutyric acid (GABA) A receptors, gamma subtype; **GABRP:** gamma-aminobutyric acid (GABA) A receptor, pi; **GABRQ:** gamma-aminobutyric acid (GABA) A receptor, theta; receptor rho1 subunit; **GABRR2:** gamma-aminobutyric acid type A receptor rho2 subunit; **GABRR3:** gamma-aminobutyric acid type A receptor rho3 subunit; **GABRs**: gamma-aminobutyric acid (GABA) receptors; **GAL3ST1**: galactose-3-O-sulfotransferase 1; **GAMT**: guanidinoacetate N-methyltransferase; **GFRA2**: GDNF family receptor alpha 2; **GH1:** growth hormone 1; **GLRs:** glycine receptors; **GLRX**: glutaredoxin (thioltransferase); **GLYAT**: glycine-N-acyltransferase; **GNAS:** GNAS complex locus; **GNB3**: guanine nucleotide binding protein (G protein), beta polypeptide 3; **GNMT**: glycine N-methyltransferase; **GPX1**: glutathione peroxidase 1; **GPR35:** G protein-coupled receptor 35; **GPX2**: glutathione peroxidase 2 (gastrointestinal); **GPX3**: glutathione peroxidase 3 (plasma); **GPX4**: glutathione peroxidase 4; **GPX5**: glutathione peroxidase 5; **GPX6**: glutathione peroxidase 6 (olfactory); **GPX7**: glutathione peroxidase 7; **GRIA1:** glutamate receptor, ionotropic, AMPA 1; **GRIA2:** glutamate receptor, ionotropic, AMPA 2; **GRIA3:** glutamate receptor, ionotropic, AMPA 3; **GRIA4**: glutamate receptor, ionotropic, AMPA 4; **GRIAs:** ionotropic glutamate receptors; **GRIK2:** glutamate receptor, ionotropic, kainate 2; **GRIK4:** glutamate receptor, ionotropic, kainate 4; **GRK5:** G protein-coupled receptor kinase 5; **GRIN1:** glutamate ionotropic receptor NMDA type subunit 1; **GRIN2A**: glutamate receptor, ionotropic, N-methyl D-aspartate 2A; **GRIN2B**: glutamate receptor, ionotropic, N-methyl D-aspartate 2B; **GRIN2C**: glutamate receptor, ionotropic, N-methyl D-aspartate 2C; **GRIN2D:** glutamate ionotropic receptor NMDA type subunit 2D; **GRIN3A:** glutamate ionotropic receptor NMDA type subunit 3A; **GRIN3B**: glutamate receptor, ionotropic, N-methyl-D-aspartate 3B; **GRM3**: glutamate receptor, metabotropic 3; **GSK3B**: glycogen synthase kinase 3 beta; **GSR**: glutathione reductase; **GSTA1**: glutathione S-transferase alpha 1; **GSTA2**: glutathione S-transferase alpha 2; **GSTA3**: glutathione S-transferase alpha 3; **GSTA4**: glutathione S-transferase alpha 4; **GSTA5**: Glutathione S-transferase alpha 5; **GSTCD**: glutathione S-transferase, C-terminal domain containing; **GSTK1**: glutathione S-transferase kappa 1; **GSTM1**: glutathione S-transferase mu 1; **GSTM2**: glutathione S-transferase mu 2 (muscle); **GSTM3**: glutathione S-transferase mu 3 (brain); **GSTM4**: glutathione S-transferase mu 4; **GSTM5**: glutathione S-transferase mu 5; **GSTO1**: glutathione S-transferase omega 1; **GSTO2**: glutathione S-transferase omega 2; **GSTP1**: glutathione S-transferase pi 1; **GSTs:** glutathione S-transferases; **GSTT1**: glutathione S-transferase theta 1; **GSTT2**: glutathione S-transferase theta 2; **GSTZ1**: glutathione S-transferase zeta 1; **GZMA**: granzyme A (granzyme 1, cytotoxic T-lymphocyte-associated serine esterase 3; **GZMB**: granzyme B (granzyme 2, cytotoxic T-lymphocyte-associated serine esterase 1); **HCRTR1:** hypocretin (orexin) receptor 1; **HCRTR2:** hypocretin (orexin) receptor 2; **HDAC2:** histone deacetylase 2; **HDAC9:** histone deacetylase 9; **HDC:** histidine decarboxylase; **HLA-B:** major histocompatibility complex, class I, B; **HNF4A:** hepatocyte nuclear factor 4, alpha; **HNMT**: histamine N-methyltransferase; **HOMER1:** homer homolog 1 (Drosophila); **HRH1**: histamine receptor H1; **HRH2**: histamine receptor H2; **HRH4:** histamine receptor H4; **HRHs**: histamine receptor family; **HSD11B1**: hydroxysteroid (11-beta) dehydrogenase 1; **HSD17B10**: hydroxysteroid (17-beta) dehydrogenase 10; **HSD17B11**: hydroxysteroid (17-beta) dehydrogenase 11; **HSD17B14**: hydroxysteroid (17-beta) dehydrogenase 14; **HTR1A**: 5-hydroxytryptamine (serotonin) receptor 1A, G protein-coupled; **HTR1B**; 5-hydroxytryptamine (serotonin) receptor 1B, G protein-coupled; **HTR1D**: 5-hydroxytryptamine (serotonin) receptor 1D, G protein-coupled; **HTR1E**: 5-hydroxytryptamine (serotonin) receptor 1E, G protein-coupled; **HTR1F**: 5-hydroxytryptamine (serotonin) receptor 1F, G protein-coupled; **HTR1s**: 5-hydroxytryptamine (serotonin) receptors, family 1; **HTR2A**: 5-hydroxytryptamine (serotonin) receptor 2A, G protein-coupled; **HTR2B**: 5-hydroxytryptamine (serotonin) receptor 2B, G protein-coupled; **HTR2C**: 5-hydroxytryptamine (serotonin) receptor 2C, G protein-coupled; **HTR2s**: 5-hydroxytryptamine (serotonin) receptors, family 2; **HTR3:** histone H3; **HTR3A**: 5-hydroxytryptamine (serotonin) receptor 3A, ionotropic**;** **HTR3B:** 5-hydroxytryptamine (serotonin) receptor 3B, ionotropic; **HTR3C**: 5-hydroxytryptamine (serotonin) receptor 3C, ionotropic; **HTR5A**: 5-hydroxytryptamine (serotonin) receptor 5A, G protein-coupled; **HTR6**: 5-hydroxytryptamine (serotonin) receptor 6, G protein-coupled; **HTR7**: 5-hydroxytryptamine (serotonin) receptor 7, adenylate cyclase-coupled; ); **HTRs:** 5-hydroxytryptamine (serotonin) receptors; **HTT**: huntingtin; **ICAM1:** intercellular adhesion molecule 1; **IDO1:** indoleamine 2,3-dioxygenase 1; **IFNA1:** interferon, alpha 1; **IGF1:** insulin-like growth factor 1 (somatomedin C); **IL1B:** interleukin 1, beta; **IL1RN**: interleukin 1 receptor antagonist; **IL6:** interleukin 6; **IL12B**: interleukin 12B; **INMT**: indolethylamine N-methyltransferase; **ITGB3:** integrin, beta 3 (platelet glycoprotein IIIa, antigen CD61); **KCNE1**: potassium channel, voltage gated subfamily E regulatory beta subunit 1; **KCNE2**: potassium channel, voltage gated subfamily E regulatory beta subunit 2; **KCNH2**: potassium channel, voltage gated eag related subfamily H, member 2; **KCNH6**: potassium channel, voltage gated eag related subfamily H, member 6; **KCNJ11**: potassium channel, inwardly rectifying subfamily J, member 11; **KCNKs**: potassium channel, subfamily K; **KCNQ1**: potassium channel, voltage gated KQT-like subfamily Q, member 1; **KRAS:** Kirsten rat sarcoma viral oncogene homolog; **LEP**: leptin; **LEPR**: leptin receptor; **LIPC:** lipase, hepatic; **LPL**: lipoprotein lipase; **MAO:** monoamine oxidase; **MAOA**: Monoamine oxidase A; **MAOB**: monoamine oxidase B; **MCHR1:** Melanin concentrating hormone receptor 1; **MET:** MET proto-oncogene, receptor tyrosine kinase; **METAP1**: Methionyl aminopeptidase 1; **MGST1**: Microsomal glutathione S-transferase 1; **MGST2**: Microsomal glutathione S-transferase 1; **MGST3**: Microsomal glutathione S-transferase 3; **MTNR1A:** melatonin receptor 1A; **MTNR1B:** melatonin receptor 1B; **NAA20**: N(alpha)-acetyltransferase 20, NatB catalytic subunit; **NAT1**: N-acetyltransferase 1 (arylamine N-acetyltransferase); **NAT2**: N-acetyltransferase 2 (arylamine N-acetyltransferase); **NDUFs:** NADH dehydrogenase (ubiquinone) family; **NNMT**: nicotinamide N-methyltransferase; **NPAS3**: neuronal PAS domain protein 3; **NPPA:** natriuretic peptide A; **NPY**: neuropeptide Y; **NQO1**: NAD(P)H dehydrogenase, quinone 1; **NQO2**: NAD(P)H dehydrogenase, quinone 2; **NR1I2**: nuclear receptor subfamily 1, group I, member 2; **NR1I3:** nuclear receptor subfamily 1, group I, member 3; **NR3C1:** nuclear receptor subfamily 3, group C, member 1 (glucocorticoid receptor); **NRG3**: neuregulin 3; **NRXN1**: neurexin 1; **NTRK1:** neurotrophic tyrosine kinase, receptor, type 1; **NTRK2:** neurotrophic tyrosine kinase, receptor, type 2; **NUBPL**: nucleotide binding protein-like; **NUDT9P1**: nudix (nucleoside diphosphate linked moiety X)-type motif 9 pseudogene 1; **OGDH:** Oxoglutarate dehydrogenase; **ORM1:** orosomucoid 1; **ORM2:** orosomucoid 2; **PALLD**: alladin, cytoskeletal associated protein; **PARK2**: parkin RBR E3 ubiquitin protein ligase; **PDE1C:** phosphodiesterase 1C, calmodulin-dependent 70kDa; **PDE5A:** phosphodiesterase 5A, cGMP-specific; **PNMT**: Phenylethanolamine N-methyltransferase; **POMC:** proopiomelanocortin; **PON1**: Paraoxonase 1; **PON2**: Paraoxonase 2; **PON3**: Paraoxonase 3; **POR**: P450 (cytochrome) oxidoreductase; **PPARA:** Peroxisome proliferator activated receptor alpha; **PPARD:** Peroxisome proliferator activated receptor delta; **PPARG:** Peroxisome proliferator activated receptor gamma; **PPARGC1A:** peroxisome proliferator-activated receptor gamma, coactivator 1 alpha; **PRKAB1:** protein kinase, AMP-activated, beta 1 non-catalytic subunit; **PRKCSH:** protein kinase C substrate 80K-H; **PRL:** prolactin; **PRLH**: prolactin releasing hormone; **PSEN1:** presenilin 1; **PSEN2:** presenilin 2; **PSMD9:** proteasome (prosome, macropain) 26S subunit, non-ATPase, 9; **PTGES**: Prostaglandin E synthase; **PTGS1**: Prostaglandin-endoperoxide synthase 1 (prostaglandin G/H synthase and cyclooxygenase); **PTGS2**: Prostaglandin-endoperoxide synthase 2 (prostaglandin G/H synthase and cyclooxygenase); **RB1:** retinoblastoma 1; **RGS2**: regulator of G-protein signaling 2; **RGS4**: regulator of G-protein signaling 4; **RGS7**: regulator of G-protein signaling 7; **RRAS2**: related RAS viral (r-ras) oncogene homolog 2; **RXRA:** retinoid X receptor, alpha; **SAT1**: spermidine/spermine N1-acetyltransferase 1; **SCL6A4:** solute carrier family 6 (neurotransmitter transporter), member 4; **SCN1A:** sodium channel, voltage gated, type I alpha subunit; **SCN3A:** Sodium voltage-gated channel alpha subunit 3; **SCN5A**: sodium channel, voltage gated, type V alpha subunit; **SCNA**: synuclein, alpha (non A4 component of amyloid precursor); **SCN1B:** sodium voltage-gated channel beta subunit 1; **SCNs:** sodium channels, voltage gated; **SIGMAR1:** sigma non-opioid intracellular receptor 1; **SLC6A2**: solute carrier family 6 (neurotransmitter transporter), member 2; **SLC6A3**: solute carrier family 6 (neurotransmitter transporter), member 3; **SLC6A4**: solute carrier family 6 (neurotransmitter transporter), member 4; **SLC10A1:** solute carrier family 10 (sodium/bile acid cotransporter), member 1; **SLC16A1:** solute carrier family 16 member 1; **SLC18A2:** solute carrier family 18 member 2; **SLC22A1:** solute carrier family 22 (organic cation transporter), member 1; **SLC22A2:** solute carrier family 22 (organic cation transporter), member 2; **SLC22A3:** solute carrier family 22 (organic cation transporter), member 3; **SLC22A4:** solute carrier family 22 member 4; **SLC22A5:** solute carrier family 22 member 5; **SLC22A6:** solute carrier family 22 member 6; **SLC22A7:** solute carrier family 22 member 7; **SLC22A8:** solute carrier family 22 member 8; **SLCO1B1:** solute carrier organic anion transporter family, member 1B1; **SLCO1B3:** solute carrier organic anion transporter family, member 1B3; **SLCO1C1:** Solute carrier organic anion transporter family member 1C1; **SLCO2A1:** Solute carrier organic anion transporter family member 2A1; **SLCO2B1:** solute carrier organic anion transporter family, member 2B1; **SLCO3A1**: solute carrier organic anion transporter family, member 3A1; **SMOX**: spermine oxidase; **SOD1**: Superoxide dismutase 1, soluble; **SOD2**: superoxide dismutase 2, mitochondrial; **SPG7:** spastic paraplegia 7 (pure and complicated autosomal recessive); **STAT3**: signal transducer and activator of transcription 3 (acute-phase response factor); **SULT1A1**: sulfotransferase family, cytosolic, 1A, phenol-preferring, member 1; **SULT1A2**: sulfotransferase family, cytosolic, 1A, phenol-preferring, member 2; **SULT1A3**: sulfotransferase family, cytosolic, 1A, phenol-preferring, member 3; **SULT1B1**: sulfotransferase family, cytosolic, 1B, member 1; **SULT1C1**: sulfotransferase family, cytosolic, 1C, member 1; **SULT1C2**: sulfotransferase family, cytosolic, 1C, member 2; **SULT1C3**: sulfotransferase family, cytosolic, 1C, member 3; **SULT1C4**: sulfotransferase family, cytosolic, 1C, member 4; **SULT1E1**: sulfotransferase family 1E, estrogen-preferring, member 1; **SULT2A1**: sulfotransferase family, cytosolic, 2A, dehydroepiandrosterone (DHEA)-preferring, member 1; **SULT2B1**: sulfotransferase family, cytosolic, 2B, member 1; **SULT4A1**: sulfotransferase family 4A, member 1; **SULT6B1**: sulfotransferase family, cytosolic, 6B, member 1; **TBX21:** T-box 21; **TBXAS1**: thromboxane A synthase 1 (platelet); **TDO2:** tryptophan 2,3-dioxygenase; **TGFB1:** transforming growth factor, beta 1; **TMEM163**: transmembrane protein 163; **TNF**: tumor necrosis factor; **TNFRSF1A:** tumor necrosis factor receptor superfamily, member 1A; **TNR**: tenascin R; **TPH1:** tryptophan hydroxylase 1; **TPH2:** tryptophan hydroxylase 2; **TPMT**: thiopurine S-methyltransferase; **TSPO:** translocator protein (18kDa); **TST**: thiopurine S-methyltransferase; **UCHL1**: ubiquitin carboxyl-terminal esterase L1 (ubiquitin thiolesterase); **UCHL3**: ubiquitin carboxyl-terminal esterase L3 (ubiquitin thiolesterase); **UGT1A1**: UDP glucuronosyltransferase 1 family, polypeptide A1; **UGT1A3**: UDP glucuronosyltransferase 1 family, polypeptide A3; **UGT1A4**: UDP glucuronosyltransferase 1 family, polypeptide A4; **UGT1A5**: UDP glucuronosyltransferase 1 family, polypeptide A5; **UGT1A6**: UDP glucuronosyltransferase 1 family, polypeptide A6; **UGT1A7**: UDP glucuronosyltransferase 1 family, polypeptide A7; **UGT1A8**: UDP glucuronosyltransferase 1 family, polypeptide A8; **UGT1A9**: UDP glucuronosyltransferase 1 family, polypeptide A9; **UGT1A10**: UDP glucuronosyltransferase 1 family, polypeptide A10; **UGT2A1**: UDP glucuronosyltransferase 2 family, polypeptide A1, complex locus; **UGT2A3**: UDP glucuronosyltransferase 2 family, polypeptide A3; **UGT2B4**: UDP glucuronosyltransferase 2 family, polypeptide B4; **UGT2B7**: UDP glucuronosyltransferase 2 family, polypeptide B7; **UGT2B10**: UDP glucuronosyltransferase 2 family, polypeptide B10; **UGT2B11**: UDP glucuronosyltransferase 2 family, polypeptide B11; **UGT2B15**: UDP glucuronosyltransferase 2 family, polypeptide B15; **UGT2B17**: UDP glucuronosyltransferase 2 family, polypeptide B17; **UGT2B28**: UDP glucuronosyltransferase 2 family, polypeptide B28; **UGT3A1**: UDP glycosyltransferase 3 family, polypeptide A1; **UGT8**: UDP glycosyltransferase 8; **UGTs**: glucuronosyltransferase family; **XDH**: Xanthine dehydrogenase; **XKR4**: XK, Kell blood group complex subunit-related family, member 4; **WARS:** tryptophanyl-tRNA synthetase; **WARS2:** tryptophanyl tRNA synthetase 2, mitochondrial.

**Table 3:** Pharmacological profile and pharmacogenetics of selected antipsychotics.

**Atypical Antipsychotics**		
**Drug**	**Properties**	**Pharmacogenetics**
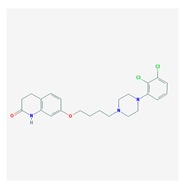	**Name: Aripiprazole**; 129722-12-9; Abilify; Abilitat; Abilify Discmelt; OPC-1459	**Pathogenic genes:***DRD2*, *DRD3*, *HTR1A*, *HTR2A*, *HTR2C* **Mechanistic genes:***ADRA1A*, *DRD2*, *DRD3*, *DRD4, HRHs*, *HTR1A*, *HTR2A*, *HTR2B, HTR2C, HTR7* **Metabolic genes:** ** Substrate:***CYP2D6* (major), *CYP3A4* (major), *CYP3A5* **Transporter genes:** *ABCB1*
	**IUPAC Name:** 7-{4-[4-(2,3-dichlorophenyl) piperazin-1-yl]butoxy}-1,2,3,4-tetrahydroquinolin-2-one	
	**Molecular Formula:** 448.38538 g/mol	
	**Molecular Weight:** C_23_H_27_Cl_2_N_3_O_2_	
	**Mechanism:** Partial agonist at the D_2_ and 5-HT_1A_ receptors, and as an antagonist at the 5-HT_2A_ receptor	
	**Effect:** Antipsychotic agent; H1-receptor antagonist; Serotonergic agonist	
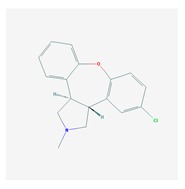	**Name: Asenapine maleate**; Saphris; Org5222 maleate; 85650-56-2; Org 5222 maleate; Org-5222 maleate	**Pathogenic genes:***ADRA2A*, *DRD1*, *DRD2*, *DRD3*, *DRD4*, *HTR1A*, *HTR2A*, *HTR2C*, *HTR7* **Mechanistic genes:***ADRA1A*, *ADRA2A*, *ADRA2B*, *ADRA2C*, *DRD1*, *DRD2*, *DRD3*, *DRD4*, *HRH1*, *HRH2*, *HTR1A*, *HTR1B*, *HTR2A*, *HTR2B*, *HTR2C*, *HTR5A*, *HTR6*, *HTR7* **Metabolic genes:** ** Substrate:***CYP1A1*, *CYP1A2* (major), *CYP2D6* (minor), *CYP3A4* (minor), *UGT1A4* ** Inhibitor:** *CYP2D6* (weak)
	**IUPAC Name:** (2Z)-but-2-enedioic acid; 17-chloro-4-methyl-13-oxa-4-azatetracyclo[12.4.0.0^2^,^6^.0^7^,^12^]octadeca-1(14),7,9,11,15,17-hexaene	
	**Molecular Formula:** C_21_H_20_ClNO_5_	
	**Molecular Weight:** 401.8402 g/mol	
	**Mechanism:** Its main activity is associated to combination of antagonistic actions at D_2_ and 5-HT_2A_ receptors	
	**Effect:** Antipsychotic agent; Dopaminergic antagonist; Serotonergic antagonist; Alpha-adrenergic antagonist; Beta-adrenergic antagonist	
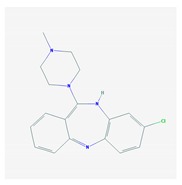	**Name: Clozapine**; Leponex; Fazaclo; Iprox; CLOZARIL; Clozapin	**Pathogenic genes:***ADRA2A*, *DRD1*, *DRD2*, *DRD3*, *DRD4*, *DTNBP1*, *HTR2A*, *LPL*, *NRXN1*, *TNF* **Mechanistic genes:***ADRAs*, *CHRMs*, *DRD1*, *DRD2*, *DRD3*, *DRD4*, *HRH1*, *HTR1F*, *HTR2A*, *HTR2C*, *HTR3A*, *HTR6*, *NRXN1* **Metabolic genes:** ** Substrate:***CYP1A2* (major), *CYP2A6* (minor), *CYP2C8* (minor), *CYP2C9* (minor), *CYP2C19* (minor), *CYP2D6* (minor), *CYP3A4/5* (major), *FMO3*, *UGT1A1, UGT1A3*, *UGT1A4* ** Inhibitor:** *CYP1A2* (weak), *CYP2C9* (moderate), *CYP2C19* (moderate), *CYP2D6* (moderate), *CYP2E1* (weak), *CYP3A4* (weak) **Transporter genes:** *ABCB1* **Pleiotropic genes:** *APOA5*, *APOC3*, *APOD*, *CNR1*, *FABP1*, *GNB3*, *GSK3B*, *LPL*, *RGS2*, *TNF*
	**IUPAC Name:** 6-chloro-10-(4-methylpiperazin-1-yl)-2,9-diazatricyclo[9.4.0.0^3^,^8^]pentadeca-1(15),3,5,7,9,11,13-heptaene	
	**Molecular Formula:** C_18_H_19_ClN_4_	
	**Molecular Weight:** 326.82326 g/mol	
	**Mechanism:** It shows serotonergic, adrenergic, and cholinergic neurotransmitter systems in addition to more selective, regionally specific effects on the mesolimbic dopaminergic system. It also displays antagonistic activity at H1-receptors	
	**Effect:** Dopaminergic antagonist; Serotonergic antagonist; Histamine antagonist; Muscarinic antagonist; GABA antagonist; GABA modulator; Antipsychotic agent	
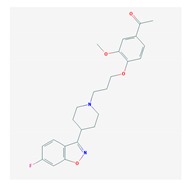	**Name: Iloperidone**; Zomaril; 133454-47-4; Fanapt; Fanapta; HP 873	**Pathogenic genes:***ADRA2A*, *CNTF*, *DRD1*, *DRD2*, *DRD3*, *DRD4*, *HTR2A*, *HTR7*, *NRG3* **Mechanistic genes:***ADRA1A*, *ADRA2A*, *ADRA2B*, *ADRA2C*, *ADRB1*, *ADRB2*, *DRD1*, *DRD2*, *DRD3*, *DRD4*, *DRD5*, *GFRA2*, *GRIA4*, *HRH1*, *HTR1A*, *HTR2A*, *HTR2C*, *HTR6*, *HTR7*, *NPAS3*, *NUDT9P1*, *TNR*, *XKR4* **Metabolic genes:** ** Substrate:***CYP1A2*, *CYP2E1*, *CYP2D6* (major), *CYP3A4* (major) **Transporter genes:** *SLC6A2*, *SLCO3A1* **Pleiotropic genes:** *ADRB2*, *CELF4*, *CERKL*, *DRD5*, *HTR1F*, *NPAS3*, *NRG3*, *NUBPL*, *PALLD*
	**IUPAC Name:** 1-(4-{3-[4-(6-fluoro-1,2-benzoxazol-3-yl)piperidin-1-yl]propoxy}-3-methoxyphenyl)ethan-1-one	
	**Molecular Formula:** C_24_H_27_FN_2_O_4_	
	**Molecular Weight:** 426.480583 g/mol	
	**Mechanism:** It has mixed D_2_/5-HT_2_ antagonist activity. It exhibits high affinity for 5-HT_2A_, D_2_, and D_3_ receptors, low to moderate affinity for D_1_, D_4_, H_1_, 5-HT_1A_, 5-HT_6_, 5HT_7_, and ADR_α__1/__α__2C_ receptors, and no affinity for muscarinic receptors. It has low affinity for histamine H_1_ receptors	
	**Effect:** Antipsychotic agent; Dopaminergic antagonist; Serotonergic antagonist; Antidepressant effects; Anxiolytic activity; Reduction of risk for weight gain; Cognitive Function Improved	
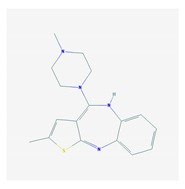	**Name: Olanzapine**; Zyprexa; 132539-06-1; Zyprexa Zydis; Olansek; Symbyax	**Pathogenic genes:***COMT*, *DRD1*, *DRD2*, *DRD3*, *DRD4*, *GRM3*, *HTR2A*, *HTR2C*, *LPL* **Mechanistic genes:***ABCB1, ADRA1A*, *ADRB3*, *AHR, BDNF, CHRM1, CHRM2, CHRM3, CHRM4, CHRM5*, *COMT*, *DRD1*, *DRD2*, *DRD3, DRD4*, *GABRs*, *GRIN2B, HRH1*, *HTR2A*, *HTR2C*, *HTR3A*, *HTR6, LEP*, *RGS2*, *RGS7*, *SLC6A4, STAT3*, *TMEM163* **Metabolic genes:** ** Substrate:***COMT*, *CYP1A2* (major), *CYP2C9, CYP2D6* (major), *CYP3A43, CYP3A5, FMO1*, *FMO3, GSTM3, TPMT, UGT1A1, UGT1A4, UGT2B10* ** Inhibitor:** *ABCB1*, *CYP1A2* (weak), *CYP2C9* (weak), *CYP2C19* (weak), *CYP2D6* (weak), *CYP3A4* (weak) ** Inducer:** *GSTM1, MAOB, SLCO3A1* **Transporter genes:** *KCNH2*, *SLC6A2, SLC6A4, SLCO3A1* **Pleiotropic genes:** *APOA5*, *APOC3*, *GNB3*, *LEP*, *LEPR*, *LPL*
	**IUPAC Name:** 5-methyl-8-(4-methylpiperazin-1-yl)-4-thia-2,9-diazatricyclo[8.4.0.0³,⁷]tetradeca-1(14),3(7),5,8,10,12-hexaene	
	**Molecular Formula:** C_17_H_20_N_4_S	
	**Molecular Weight:** 312.4325 g/mol	
	**Mechanism:** It displays potent antagonism of serotonin 5-HT_2A_ and 5-HT_2C_, dopamine D_1-4_, histamine H_1_ and α_1_-adrenergic receptors. It shows moderate antagonism of 5-HT_3_ and muscarinic M_1-5_ receptors, and weak binding to GABA-A, BZD, and β-adrenergic receptors.	
	**Effect:** Antipsychotic agent; GABA modulator; Muscarinic antagonist; Serotonin uptake inhibitor; Dopaminergic antagonist; Serotonergic antagonist; Histamine antagonist; Antiemetic activity	
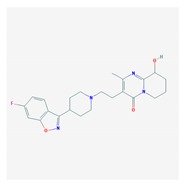	**Name: Paliperidone**; Paliperidone; 9-Hydroxyrisperidone; Invega; 144598-75-4; 9-OH-risperidone; Invega Sustenna	**Pathogenic genes:***ADRA2A*, *DRD2*, *HTR2A* **Mechanistic genes:***ADRA1A*, *ADRA1B*, *ADRA1D*, *ADRA2s*, *BDNF, DRD2*, *HRH1*, *HTR1A*, *HTR2A*, *HTR2C* **Metabolic genes:** ** Substrate:***ADH*, *CYP2D6*(minor), *CYP3A4/5* (major), *UGTs* ** Inhibitor:** *ABCB1*, *CYP2D6* (moderate), *CYP3A4/5* (moderate) **Transporter genes:** *ABCB1*
	**IUPAC Name:** 3-{2-[4-(6-fluoro-1,2-benzoxazol-3-yl)piperidin-1-yl]ethyl}-9-hydroxy-2-methyl-4H,6H,7H,8H,9H-pyrido[1,2-a]pyrimidin-4-one	
	**Molecular Formula:** C_23_H_27_FN_4_O_3_	
	**Molecular Weight:** 426.483883 g/mol	
	**Mechanism:** Mixed central serotonergic and dopaminergic antagonism. Demonstrates high affinity to α_1_, D_2_, H_1_, and 5-HT_2C_ receptors, and low affinity for muscarinic and 5-HT_1A_ receptors	
	**Effect:** Antipsychotic agent; Neuroprotective agent; H1-receptor antagonist; Alpha-adrenergic antagonist; Serotonergic antagonist; Dopaminergic antagonist	
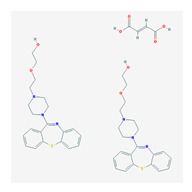	**Name: Quetiapine Fumarate**; Seroquel; Quetiapine hemifumarate; 111974-72-2; Seroquel XR; UNII-2S3PL1B6UJ	**Pathogenic genes:***ADRA2A*, *DRD1*, *DRD2*, *DRD4*, *HTR1A*, *HTR2A*, *RGS4* **Mechanistic genes:***ADRA1s*, *ADRA2s*, *BDNF, CHRM1, CHRM3, CHRM5, DRD1*, *DRD2*, *DRD4*, *HRH1*, *HTR1A*, *HTR1E, HTR2A*, *HTR2B, HTR7* **Metabolic genes:** ** Substrate:***CYP2D6* (minor), *CYP3A4/5* (major), *CYP3A7*, *CYP2C19* ** Inhibitor:** *ABCB1, SLC6A2* **Transporter genes:** *ABCB1*, *KCNE1*, *KCNE2*, *KCNH2*, *KCNQ1*, *SCN5A, SLC6A2*
	**IUPAC Name:** 2-[2-(4-{2-thia-9-azatricyclo[9.4.0.0³,⁸]pentadeca-1(15),3,5,7,9,11,13-heptaen-10-yl}piperazin-1-yl)ethoxy]ethan-1-ol	
	**Molecular Formula:** C_46_H_54_N_6_O_8_S_2_	
	**Molecular Weight:** 883.08636 g/mol	
	**Mechanism:** Antagonist at multiple neurotransmitter receptors: serotonin 5-HT_1A_ and 5-HT_2_, dopamine D_1_ and D_2_, histamine H_1_, and adrenergic α_1_- and α_2_-receptors	
	**Effect:** Antipsychotic agent; Adrenergic antagonist; Histamine antagonist; Serotonergic antagonist; Dopaminergic antagonist; Sedative activity; Orthostatic hypotension	
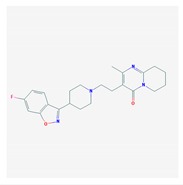	**Name: Risperidone**; Risperdal; Risperidal; 106266-06-2; Risperdal Consta; Rispolept	**Pathogenic genes:***ADRA2A*, *BDNF*, *COMT*, *DRD1*, *DRD2*, *DRD3*, *DRD4*, *GRM3*, *HTR2A*, *HTR2C*, *HTR7, PON1*, *RGS4* **Mechanistic genes:***ADRA1A*, *ADRA1B*, *ADRA2s*, *DRD1*, *DRD2*, *DRD3*, *DRD4*, *FOS*, *HTR2A*, *HTR2C*, *HTR3A*, *HTR3C*, *HTR6*, *HTR7, NR1I2, STAT3* **Metabolic genes:** ** Substrate:***COMT, CYP2D6* (major), *CYP3A4/5* (minor) ** Inhibitor:** *ABCB1*, *CYP2D6* (weak), *CYP3A4* (weak) Inducer: *MAOB* **Transporter genes:** *ABCB1, KCNH2*, *SLC6A4* **Pleiotropic genes:** *APOA5, BDNF, RGS2*
	**IUPAC Name:** 3-{2-[4-(6-fluoro-1,2-benzoxazol-3-yl)piperidin-1-yl]ethyl}-2-methyl-4H,6H,7H,8H,9H-pyrido[1,2-a]pyrimidin-4-one	
	**Molecular Formula:** C_23_H_27_FN_4_O_2_	
	**Molecular Weight:** 410.484483 g/mol	
	**Mechanism:** Antagonist at multiple neurotransmitter receptors: serotonin 5-HT_1A_ and 5-HT_2_, dopamine D_1_ and D_2_, histamine H_1_, and adrenergic α_1_- and α_2_-receptors	
	**Effect:** Antipsychotic agent; H_1_-receptor antagonist; Dopaminergic antagonist; Alpha-adrenergic antagonist; Serotonergic antagonist; Somnolence; Orthostatic hypotension	
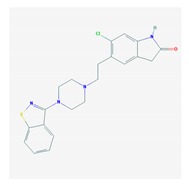	**Name: Ziprasidone**; Geodon; 146939-27-7; Zeldox; Ziprazidone; Ziprasidone hydrochloride	**Pathogenic genes:***DRD2*, *DRD3*, *DRD4*, *HTR1A*, *HTR2A*, *HTR2C*, *RGS4* **Mechanistic genes:***ADRA1A*, *DRD2*, *DRD3*, *HRH1*, *HTR1A*, *HTR1B, HTR1D*, *HTR2A*, *HTR2C* **Metabolic genes:** ** Substrate:***AOX1* (major), *CYP1A2* (minor), *CYP3A4* (major) ** Inhibitor:** *CYP2D6* (moderate), *CYP3A4* (moderate), *SLC6A2, SLC6A4* **Transporter genes:** *KCNH2, SLC6A2, SLC6A4* **Pleiotropic genes:** *CHRM1*, *RRAS2*
	**IUPAC Name:** 5-{2-[4-(1,2-benzothiazol-3-yl)piperazin-1-yl]ethyl}-6-chloro-2,3-dihydro-1H-indol-2-one	
	**Molecular Formula:** C_21_H_21_ClN_4_OS	
	**Molecular Weight:** 412.93564 g/mol	
	**Mechanism:** It has high affinity for D_2_, D_3_, 5-HT_2A_, 5-HT_1A_, 5-HT_2C_, 5-HT_1D_, and α_1_-adrenergic, and moderate affinity for histamine H_1_ receptors. It functions as antagonist at D_2_, 5-HT_2A_, and 5-HT_1D_ receptors and as agonist at 5-HT_1A_ receptor. It moderately inhibits reuptake of serotonin and norepinephrine	
	**Effect:** Antipsychotic agent; Histamine antagonist; Dopaminergic antagonist; Serotonergic antagonist; Muscarinic antagonist; Serotonin–norepinephrine reuptake inhibitor	
**Typical Antipsychotics**		
**Butyrophenones**		
**Drug**	**Properties**	**Pharmacogenetics**
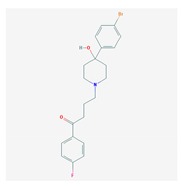	**Name: Bromperidol**; Impromen; Bromoperidol; Tesoprel; 10457-90-6; Azurene	**Pathogenic genes:***DRD2*, *HTR2A* **Mechanistic genes:***DRD2*, *HTR2A* **Metabolic genes:** ** Substrate:***CYP2D6* (minor), *CYP3A4* (major), *UGTs* ** Inhibitor:** *CYP2D6* (moderate) **Transporter genes:** *ABCB1*
	**IUPAC Name:** 4-[4-(4-bromophenyl)-4-hydroxypiperidin-1-yl]-1-(4-fluorophenyl)butan-1-one	
	**Molecular Formula:** C_21_H_23_BrFNO_2_	
	**Molecular Weight:** 420.315223 g/mol	
	**Mechanism:** Potent dopaminergic D_2_ antagonist. Has weak α_1_-adrenolitic activity. It is a moderate serotonin 5-HT_2_ antagonist. Has no antihistaminic or anticholinergic effects. It acts on the mesocortex, limbic system, and basal ganglia (nigrostriate pathway)	
	**Effect:** Antipsychotic agent; Dopamine antagonist; α_1_-adrenolitic activity	
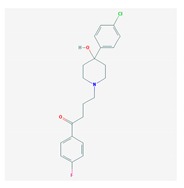	**Name: Haloperidol**; Haldol; Eukystol; Serenace; Aloperidin; Aloperidol	**Pathogenic genes:***ADRA2A, BDNF, DRD1, DRD2, DRD4, DTNBP1, GRIN2B, HTR2A* **Mechanistic genes:***ANKK1, BDNF, COMT, DRD1, DRD2, DTNBP1, GRIN2A, GRIN3B, GRIN2C, GRIN2B, SLC6A3* **Metabolic genes:** ** Substrate:***CBR1*, *CYP1A1* (minor), *CYP1A2* (minor), *CYP2A6*, *CYP2C8* (minor), *CYP2C9* (minor), *CYP2C19* (minor), *CYP2D6* (major), *CYP3A4*/*5* (major), *GSTP1*, *UGTs* ** Inhibitor:** *ABCB1, CYP2D6* (moderate), *CYP3A4* (moderate) **Transporter genes:** *ABCB1, ABCC1, KCNE1, KCNE2, KCNH2, KCNJ11, KCNQ1, SLC6A3* **Pleiotropic genes:** *CHRM2, FOS, GSK3B, HRH1, HTR2A, HTT, IL1RN*
	**IUPAC Name:** 4-[4-(4-chlorophenyl)-4-hydroxypiperidin-1-yl]-1-(4-fluorophenyl)butan-1-one	
	**Molecular Formula:** C_21_H_23_ClFNO_2_	
	**Molecular Weight:** 375.864223 g/mol	
	**Mechanism:** Haloperidol is a butyrophenone antipsychotic which blocks postsynaptic mesolimbic dopaminergic D_1_ and D_2_ receptors in brain. Depresses release of hypothalamic and hypophyseal hormones. Believed to depress reticular activating system	
	**Effect:** Antipsychotic agent; Serotonergic antagonist; Dopaminergic antagonist; Antiemetic; Antidyskinesia agent; Sedative effects; Hypotension	
**Phenothiazines**		
**Drug**	**Properties**	**Pharmacogenetics**
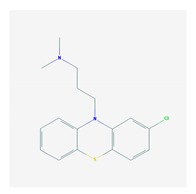	**Name: Chlorpromazine**; Largactil; Thorazine; Contomin; Chloropromazine; Aminazine	**Pathogenic genes:***BDNF*, *DRD1*, *DRD2*, *DRD3*, *DRD4*, *HTR2A* **Mechanistic genes:***ADRA1A, ADRA1B, CHRM1, CHRM2, DRD1, DRD2, DRD3, DRD4, HRH1, HTR1A, HTR2A, HTR2C* **Metabolic genes:** ** Substrate:***CYP1A2* (minor), *CYP2A6, CYP2C9, CYP2C19, CYP2D6* (major), *CYP3A4* (minor), *FMO1*, *UGT1A3*, *UGT1A4* ** Inhibitor:** *CYP1A2, CYP2D6* (strong), *CYP2C19, CYP2E1* (weak), *CYP3A4, DAO* **Transporter genes:** *ABCB1*, *CFTR* **Pleiotropic genes:** *ACACA, BDNF, FABP1, LEP, NPY*
	**IUPAC Name:** [3-(2-chloro-10H-phenothiazin-10-yl)propyl]dimethylamine	
	**Molecular Formula:** C_17_H_19_ClN_2_S	
	**Molecular Weight:** 318.86416 g/mol	
	**Mechanism:** Blocks postsynaptic mesolimbic dopaminergic receptors in the brain. Has actions at all levels of CNS, particularly at subcortical levels; also acts on multiple organ systems. Also exhibits weak ganglionic blocking, has a strong α-adrenergic blocking effect and depresses the release of hypothalamic and hypophyseal hormones. Depresses the reticular activating system	
	**Effect:** Antipsychotic agent; Dopaminergic antagonist; Antiemetic; Anticholinergic effects; Sedative effects; Antihistaminic effects; Anti-serotonergic activity; Hypotension	
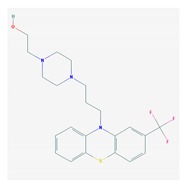	**Name: Fluphenazine**; Triflumethazine; Fluorophenazine; Fluorfenazine; Fluorphenazine; Siqualine	**Pathogenic genes:***DRD1, DRD2* **Mechanistic genes:***CALM1, DRD1, DRD2* **Metabolic genes:** ** Substrate:***CYP2B6, CYP2D6* (major) ** Inhibitor:** *ABCB1, CYP1A2* (weak), *CYP2A6, CYP2C9* (weak), *CYP2C19, CYP2D6* (strong), *CYP2E1* (weak), *CYP3A4* **Transporter genes:** *ABCB1*
	**IUPAC Name:** 2-(4-{3-[2-(trifluoromethyl)-10H-phenothiazin-10-yl]propyl}piperazin-1-yl)ethan-1-ol	
	**Molecular Formula:** C_22_H_26_F_3_N_3_OS	
	**Molecular Weight:** 437.52155 g/mol	
	**Mechanism:** Blocks postsynaptic mesolimbic dopaminergic D_1_ and D_2_ receptors in the brain. Depresses release of hypothalamic and hypophyseal hormones. Believed to depress reticular activating system, thus affecting basal metabolism, body temperature, wakefulness, vasomotor tone, and emesis	
	**Effect:** Antipsychotic agent; Dopaminergic antagonist; Antiemesis; Anticholinergic effects; Sedative effects	
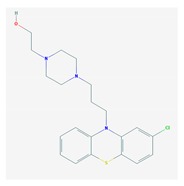	**Name: Perphenazine**; Trilafon; Perphenazin; Etaperazine; Perfenazine; Fentazin	**Pathogenic genes:***ADRA2A*, *DRD1*, *DRD2*, *HTR2A, HTR2C, RGS4* **Mechanistic genes:***ADRA1A*, *ADRA2A*, *DRD1*, *DRD2*, *HTR2A, HTR2C, RGS4* **Metabolic genes:** ** Substrate:***CYP1A2* (major), *CYP2C8* (major), *CYP2C9* (major), *CYP2C18* (major), *CYP2C19* (major), *CYP2D6* (minor), *CYP3A4/5* (major) ** Inhibitor:** *CYP1A2* (weak), *CYP2D6* (strong) **Transporter genes:** *ABCB1*
	**IUPAC Name:** 2-[4-[3-(2-chlorophenothiazin-10-yl)propyl]piperazin-1-yl]ethanol	
	**Molecular Formula:** C_21_H_26_ClN_3_OS	
	**Molecular Weight:** 403.96864 g/mol	
	**Mechanism:** Blocks postsynaptic mesolimbic dopaminergic receptors in brain. Exhibits α-adrenergic-blocking effect and depresses release of hypothalamic and hypophyseal hormones. Binds to the dopamine D_1_ and D_2_ receptors and inhibits their activity	
	**Effect:** Antipsychotic agent; Antiemetic; Dopaminergic antagonist; Antiemesis	
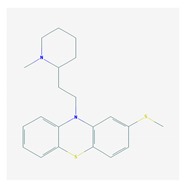	**Name: Thioridazine**; Mellaril; Melleril; Thioridazin; Meleril; Sonapax	**Pathogenic genes:***ADRA2A*, *DRD1*, *DRD2* **Mechanistic genes:***ADRA1s*, *ADRA2s*, *DRD1*, *DRD2* **Metabolic genes:** ** Substrate:***CYP1A2* (major), *CYP2C19* (minor), *CYP2A6*, *CYP2D6* (major), *CYP2J2* (major), *CYP3A4* (major) ** Inhibitor:** *ABCB1, CYP1A2* (weak), *CYP2A6, CYP2C9* (weak), *CYP2C19* (weak)*, CYP2D6* (moderate), *CYP2E1* (weak), *CYP3A4* (weak), *KCNH* **Transporter genes:** *ABCB1*, *KCNE1*, *KCNE2*, *KCNH2*, *KCNJ11*, *KCNQ1* **Pleiotropic genes:** *ADRBs*, *CHRM2*, *FABP1*, *HRH1*
	**IUPAC Name:** 10-[2-(1-methylpiperidin-2-yl)ethyl]-2-methylsulfanylphenothiazine	
	**Molecular Formula:** C_21_H_26_N_2_S_2_	
	**Molecular Weight:** 370.57454 g/mol	
	**Mechanism:** Blocks postsynaptic mesolimbic dopaminergic receptors in brain. Exhibits strong α-adrenergic-blocking effect and depresses release of hypothalamic and hypophyseal hormones	
	**Effect:** Antipsychotic agent; Dopaminergic antagonist; Alpha-adrenergic antagonist; H_1_-receptor antagonist; Serotonergic antagonist	
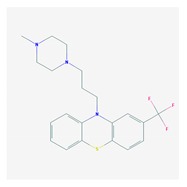	**Name: Trifluoperazine**; Trifluperazine; Trifluoroperazine; Trifluoperazina; Triflurin; Triperazine	**Pathogenic genes:***DRD2*, *IL12B* **Mechanistic genes:***ABCB1, ADRA1A*, *ADRA1B, CALMs, DRD2, HRH1* **Metabolic genes:** ** Substrate:***CYP1A2* (major), *UGT1A4* ** Inhibitor:** *CALMs, POR* **Transporter genes:** *ABCB1* **Pleiotropic genes:** *IL12B*
	**IUPAC Name:** 10-[3-(4-methylpiperazin-1-yl)propyl]-2-(trifluoromethyl)-10H-phenothiazine	
	**Molecular Formula:** C_21_H_24_F_3_N_3_S	
	**Molecular Weight:** 407.49557 g/mol	
	**Mechanism:** Blocks postsynaptic mesolimbic dopaminergic receptors in brain. Exhibits α-adrenergic-blocking effect and depresses release of hypothalamic and hypophyseal hormones. This agent also functions as a calmodulin inhibitor	
	**Effect:** Antipsychotic agent; Dopaminergic antagonist; Antiemesis; Cytosolic calcium elevation	
**Thioxanthenes**		
**Drug**	**Properties**	**Pharmacogenetics**
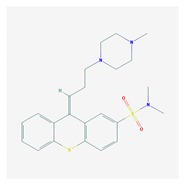	**Name: Thiothixene**, Tiotixene; Cis-Thiothixene; Navane; Thiothixine; Navan	**Pathogenic genes:***ADRA2A*, *DRD1*, *DRD2*, *DRD3*, *DRD4*, *HTR2A* **Mechanistic genes:***ADRA1s*, *ADRA2s*, *CHRM1*, *CHRM2*, *DRD1*, *DRD2*, *DRD3*, *DRD4*, *HRH1, HRH2*, *HTR1s*, *HTR2s* **Metabolic genes:** ** Substrate:***CYP1A2* (major) ** Inhibitor:** *CYP2D6* (moderate) **Transporter genes:** *KCNE1*, *KCNE2*, *KCNH6*, *KCNQ1*, *SCN5A*
	**IUPAC Name:** (9Z)-N,N-dimethyl-9-[3-(4-methylpiperazin-1-yl)propylidene]thioxanthene-2-sulfonamide	
	**Molecular Formula:** C_23_H_29_N_3_O_2_S_2_	
	**Molecular Weight:** 443.62526 g/mol	
	**Mechanism:** Elicits antipsychotic activity by postsynaptic blockade of CNS dopamine receptors resulting in inhibition of dopamine-mediated effects. Also has α-adrenergic blocking activity. Antagonistic effect on dopaminergic (D_1_, D_2_, D_3_, D_4_), histaminergic (H_1_), sertoninergic (5-HT_1_, 5-HT_2_), adrenergic (α_1_- and α_2_) and muscarinic (M_1_ and M_2_) receptors	
	**Effect:** Antipsychotic agent; Dopamine antagonist; Antisympathomimetic properties; Anticholinergic effects; Antidepressant effects, Antiaggressive properties; H_1_-receptor antagonist; Serotonergic agonist	
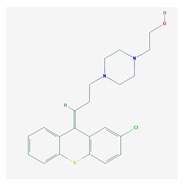	**Name: Zuclopenthixol**; Zuclopentixol; Clopixol; Zuclopenthixolum; Acuphase; Cisordinol	**Pathogenic genes:***ADRA2A*, *DRD1*, *DRD2* **Mechanistic genes:***ADRA1s*, *ADRA2s*, *CHRMs*, *DRD1*, *DRD2*, *HRH1*, *HTR2s* **Metabolic genes:** ** Substrate:*CYP2D6* (major), *CYP3A4* (major)**
	**IUPAC Name:** 2-[4-[(3Z)-3-(2-chlorothioxanthen-9-ylidene)propyl]piperazin-1-yl]ethanol	
	**Molecular Formula:** C_22_H_25_ClN_2_OS	
	**Molecular Weight:** 400.9647 g/mol	
	**Mechanism:** It mainly acts by antagonism of D_1_ and D_2_ dopamine receptors. It also has high affinity for alpha1-adrenergic and 5-HT_2_ receptors. It has weaker histamine H_1_ receptor blocking activity, and even lower affinity for muscarinic cholinergic and alpha2-adrenergic receptors	
	**Effect:** Antipsychotic agent; Dopaminergic antagonist; H_1_-receptor antagonist; Serotonergic antagonist; Alpha-adrenergic antagonist.	
**Typical Antipsychotics, Miscellaneous**		
**Drug**	**Properties**	**Pharmacogenetics**
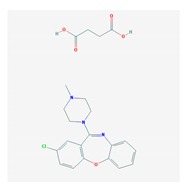	**Name: Loxapine Succinate**; 27833-64-3; Loxapac; Loxapine succinate salt; Cloxazepin; Daxolin	**Pathogenic genes:***DRD1*, *DRD2*, *HTR2A* **Mechanistic genes:***DRD1*, *DRD2*, *HTR2A* **Metabolic genes:** ** Substrate:***CYP1A2* (major), *CYP2D6* (major), *CYP3A4* (major), *FMOs*, *UGT1A4* **Pleiotropic genes:** *HTR2A*
	**IUPAC Name:** butanedioic acid;8-chloro-6-(4-methylpiperazin-1-yl)benzo[b][1,4]benzoxazepine	
	**Molecular Formula:** C_22_H_24_ClN_3_O_5_	
	**Molecular Weight:** 445.89606 g/mol	
	**Mechanism:** Blocks postsynaptic mesolimbic D_1_ and D_2_ receptors in brain; also possesses serotonin 5-HT_2_-blocking activity	
	**Effect:** Antipsychotic agent; Dopamine antagonist; Serotonergic antagonist	
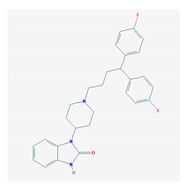	**Name: Pimozide**; Orap; Opiran; Neoperidole; Pimozidum; 2062-78-4	**Mechanistic genes:***ADRA1A*, *CACNs*, *DRD1*, *DRD2*, *KCNH2* **Metabolic genes:** ** Substrate:***CYP1A2* (minor), *CYP2D6* (minor), *CYP3A4* (major) ** Inhibitor:** *ABCB1*, *CYP2C19* (weak), *CYP2D6* (strong), *CYP2E1* (weak), *CYP3A4* (moderate), *KCNH2* **Transporter genes:** *KCNE1*, *KCNE2*, *KCNH2*, *KCNJ11*, *KCNKs, KCNQ1*, *SCN5A* **Pleiotropic genes:** *CHRM2*
	**IUPAC Name:** 3-[1-[4,4-bis(4-fluorophenyl)butyl]piperidin-4-yl]-1H-benzimidazol-2-one	
	**Molecular Formula:** C_28_H_29_F_2_N_3_O	
	**Molecular Weight:** 461.546166 g/mol	
	**Mechanism:** It is a potent centrally-acting dopamine-receptor antagonist resulting in its characteristic neuroleptic effects. Binds and inhibits the dopamine D2 receptor in the CNS. It is antagonist of ADRA_1A_	
	**Effect:** Antipsychotic agent; H1-receptor antagonist; Dopaminergic antagonist; Antidyskinesia agent; Serotonergic antagonist	
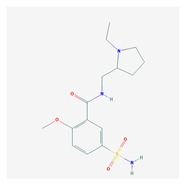	**Name: Sulpiride**; Sulpyrid; Aiglonyl; Dogmatil; Dolmatil; Sulpirid	**Pathogenic genes:***DRD2*, *DRD* **Mechanistic genes:***DRD2*, *DRD3*, *PRLH* **Metabolic genes:** ** Substrate:***CYP1A2*, *CYP2B1*, *CYP3As* ** Inhibitor:***CYP1A2, CYP2B1, CYP3As*
	**IUPAC Name:** N-[(1-ethylpyrrolidin-2-yl)methyl]-2-methoxy-5-sulfamoylbenzamide	
	**Molecular Formula:** C_15_H_23_N_3_O_4_S	
	**Molecular Weight:** 341.42582 g/mol	
	**Mechanism:** It is a selective antagonist at postsynaptic D_2_ and D_3_-receptors. It appears to lack effects on norepinephrine, acetylcholine, serotonin, histamine, or GABA receptors. It also stimulates secretion of prolactin	
	**Effect:** Antipsychotic agent; Dopaminergic antagonist; Antidepressant effect; Antiemesis. Sedation (>600 mg/day); Dopamine reuptake inhibition (<200 mg/day); Antiemesis, Antimigraine effects; Antivertiginous activity; Prolactin-releasing stimulation	

**Table 4:** Pharmacological profile and pharmacogenetics of selected antidepressants.

**Monoamine Oxidase Inhibitors (MAOIs)**		
**Drug**	**Properties**	**Pharmacogenetics**
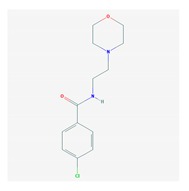	**Name: Moclobemide**; Aurorix; Moclamine; Manerix; Moclobemidum; Moclobemid	**Pathogenic genes:***MAOA* **Mechanistic genes:***MAOA* **Metabolic genes:** **Substrate:***CYP2C19* (major), *CYP2D6* (major), CYP2E1 **Inhibitor:** *CYP1A2* (weak)*, CYP2C19* (weak), *CYP2D6* (weak), *MAOA* (strong), *MAOB* (moderate)
	**IUPAC Name**: 4-chloro-N-[2-(morpholin-4-yl)ethyl]benzamide	
	**Molecular Formula:** C_13_H_17_ClN_2_O_2_	
	**Molecular Weight:** 268.73928 g/mol	
	**Category:** Monoamine oxidase A inhibitors	
	**Mechanism:** Involves the selective, reversible inhibition of MAO-A. This inhibition leads to a decrease in the metabolism and destruction of monoamines in the neurotransmitters. This results in an increase in the monoamines.	
	**Effect:** Antidepressant agent; Monoamine Oxidase inhibition.	
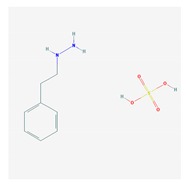	**Name: Phenelzine sulfate**; Estinerval; Nardelzine; Kalgan; Phenethylhydrazin; Phenelzine sulfate salt	**Pathogenic genes:***MAOA* **Mechanistic genes:***MAOA, MAOB* **Metabolic genes:** **Substrate:***COMT, MAOA, MAOB* ** Inhibitor:***CYP2C8* (moderate), *CYP2D6, CYP3A4* (moderate), *MAOA, MAOB*
	**IUPAC Name**: (2-phenylethyl)hydrazine	
	**Molecular Formula:** C_8_H_14_N_2_O_4_S	
	**Molecular Weight:** 234.27276 g/mol	
	**Category:** Monoamine oxidase inhibitors, non-selective	
	**Mechanism:** Irreversible, non-selective inhibition of MAO. It causes an increase in the levels of serotonin, norepinephrine, and dopamine in the neuron	
	**Effect:** Antidepressant activity; Monoamine Oxidase inhibition	
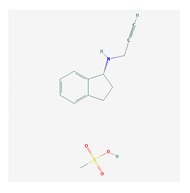	**Name: Rasagiline mesylate**; 161735-79-1; Azilect; Rasagiline mesilate; TVP-1012; Agilect	**Pathogenic genes:***PARK2* **Mechanistic genes:***BCL2, BCL2L1, MAOB* **Metabolic genes:** ** Substrate:***CYP1A2* (major), *CYP2D6*, *UGT1A1*, *UGT1A3*, *UGT1A4*, *UGT1A6*, *UGT1A7*, *UGT1A9*, *UGT1A10*, *UGT2B7*, *UGT2B15* ** Inhibitor:** *MAOB*
	**IUPAC Name**: (1R)-N-(prop-2-yn-1-yl)-2,3-dihydro-1H-inden-1-amine	
	**Molecular Formula:** C_13_H_17_NO_3_S	
	**Molecular Weight:** 267.34398 g/mol	
	**Category:** Monoamine oxidase B inhibitors	
	**Mechanism:** Potent, irreversible, selective inhibitor of brain monoamine oxidase (MAO) type B, which plays a major role in catabolism of dopamine.	
	**Effect:** Antidepressant activity; Monoamine Oxidase inhibition; Neuroprotective agent; Antiparkinsonian agent.	
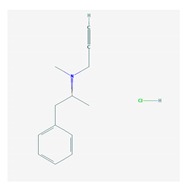	**Name: Selegiline**; Selegiline hydrochloride; L-Deprenyl hydrochloride; 14611-52-0; Eldepryl; Selegiline Hcl; Zelapar	**Pathogenic genes:***MAOA, PARK2* **Mechanistic genes:***MAOA, MAOB* **Metabolic genes:** ** Substrate:***CYP1A2* (minor), *CYP2A6* (minor), *CYP2B6* (major), *CYP2C8* (minor), *CYP2C19* (major), *CYP2D6* (minor), *CYP2E1* (minor), *CYP3A4* (minor), *MAOA* ** Inhibitor:** *CYP1A2* (weak), *CYP2A6* (weak), *CYP2C9* (weak), *CYP2C19* (weak), *CYP2D6* (weak), *CYP2E1* (weak), *CYP3A4* (weak), *MAOB* (strong), *MAOA* **Transporter genes:** *SCNA*
	**IUPAC Name**: methyl(1-phenylpropan-2-yl)(prop-2-yn-1-yl)amine	
	**Molecular Formula:** C_13_H_18_ClN	
	**Molecular Weight:** 223.74172 g/mol	
	**Category:** Monoamine oxidase B inhibitors	
	**Mechanism:** Selective, irreversible inhibition of MAO-B. It binds to MAO-B within the nigrostriatal pathways in the central nervous system, thus blocking microsomal metabolism of dopamine and enhancing the dopaminergic activity in the substantia nigra. It may also increase dopaminergic activity through mechanisms other than inhibition of MAO-B. At higher doses, it can also inhibit MAO-A.	
	**Effect:** Antidepressant activity; Monoamine Oxidase inhibition Neuroprotective agent; Antiparkinsonian agent.	
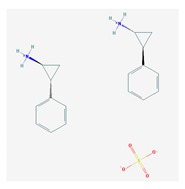	**Name: Tranylcypromine sulphate**; Tylciprine; Phenylcyclopromine sulfate; Dl-Tranylcypromine sulfate; EINECS 236-807-1; 1-Amino-2-phenylcyclopropane sulfate	**Pathogenic genes:***MAOA, NTRK2, SLC6A4* **Mechanistic genes:***MAOA, MAOB* **Metabolic genes:** ** Substrate:***CYP2A6* (major) ** Inhibitor:** *CYP1A2* (moderate), *CYP2A6* (strong), *CYP2C8* (weak), *CYP2C9* (weak), *CYP2C19* (moderate), *CYP2D6* (moderate), *CYP2E1* (weak), *CYP3A4* (weak), *MAOA, MAOB* **Transporter genes:** *SLC6A4* **Pleiotropic genes:** *FOS, NTRK2*
	**IUPAC Name**: (1R)-2-phenylcyclopropan-1-amine	
	**Molecular Formula:** C_18_H_24_N_2_O_4_S	
	**Molecular Weight:** 364.45916 g/mol	
	**Category:** Monoamine oxidase inhibitors, non-selective	
	**Mechanism:** It increases endogenous concentrations of epinephrine, norepinephrine, dopamine, serotonin through inhibition of MAO responsible for breakdown of these neurotransmitters	
	**Effect:** Monoamine Oxidase inhibition; Antidepressant activity; Anti-anxiety activity	
**Tricyclics (TCA) and other Norepinephrinereuptake inhibitors**		
**Drug**	**Properties**	**Pharmacogenetics**
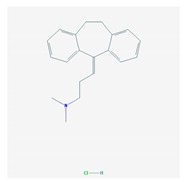	**Name: Amitriptyline Hydrochloride**; Annoyltin; Amitriptyline HCl; 549-18-8; Tryptizol; Domical	**Pathogenic genes:***ABCB1*, *GNB3*, *HTRs*, *NTRK2*, *SLC6A4*, *TNF* **Mechanistic genes:***ADRA1A*, *HTRs*, *NTRK1*, *NTRK2* **Metabolic genes:** ** Substrate:***ABCB1*, *CYP1A2* (minor), *CYP2B6* (minor), *CYP2C9* (minor), *CYP2C19* (minor), *CYP2D6* (major), *CYP3A4/5* (major), *GSTP1, UGT1A3*, *UGT1A4*, *UGT2B10* ** Inhibitor:** *ABCB1*, *ABCC2*, *ABCG2*, *CYP1A2* (moderate), *CYP2C9* (moderate), *CYP2C19* (moderate), *CYP2D6* (moderate), *CYP2E1* (weak) **Transporter genes:** *ABCB1*, *ABCC2*, *ABCG2*, *KCNE2*, *KCNH2*, *KCNQ1*, *SCN5A*, *SLC6A4* **Pleiotropic genes:** *FABP1*, *GNAS*, *GNB3*, *NTRK1*, *TNF*
	**IUPAC Name**: dimethyl(3-{tricyclo[9.4.0.0³,⁸]pentadeca-1(15),3,5,7,11,13-hexaen-2-ylidene}propyl)amine	
	**Molecular Formula:** C_20_H_24_ClN	
	**Molecular Weight:** 313.86426 g/mol	
	**Category:** Tricyclics	
	**Mechanism:** Increases synaptic concentration of serotonin and/or norepinephrine in the central nervous system by inhibiting their reuptake in the presynaptic neuronal membrane	
	**Effect:** Adrenergic Uptake inhibition; Antimigraine activity; Analgesic (nonnarcotic) activity; Antidepressant action	
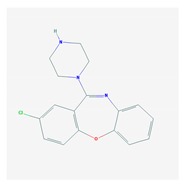	**Name: Amoxapine**; Asendin; Demolox; 14028-44-5; Asendis; Moxadi	**Pathogenic genes:***GNB3*, *SLC6A4* **Mechanistic genes:***ADRA1A*, *ADRA2A*, *CHRMs*, *DRD1*, *DRD2*, *GABRs*, *GABBRs*, *HTRs* **Metabolic genes:** ** Substrate:***CYP2D6* (major) **Transporter genes:** *SLC6A2*, *SLC6A4* **Pleiotropic genes:** *DRD2*, *GNAS*, *GNB3*
	**IUPAC Name**: 13-chloro-10-(piperazin-1-yl)-2-oxa-9-azatricyclo[9.4.0.0³,⁸]pentadeca-1(11),3,5,7,9,12,14-heptaene	
	**Molecular Formula:** C_17_H_16_ClN_3_O	
	**Molecular Weight:** 313.78144 g/mol	
	**Category:** Tricyclics	
	**Mechanism:** Reduces reuptake of serotonin and norepinephrine. The metabolite, 7-OH-amoxapine, has significant dopamine receptor-blocking activity	
	**Effect:** Serotonin uptake inhibition; Adrenergic uptake inhibition; Dopamine antagonism; Neurotransmitter uptake inhibition; Antidepressant action; Anti-anxiety activity	
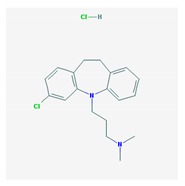	**Name: Clomipramine Hydrochloride**; Anafranil; Clomipramine HCL; 17321-77-6; Anaphranil; 3-(3-chloro-10,11-dihydro-5H-dibenzo[b,f]azepin-5-yl)-N,N-dimethylpropan-1-amine hydrochloride	**Pathogenic genes:***HTR2A*, *SLC6A4* **Mechanistic genes:***ADRA1s*, *CHRMs*, *CHRNs*, *HRH1*, *HTR2s, HTR3* **Metabolic genes:** ** Substrate:***CYP1A2* (major), *CYP2A6*, *CYP2B6*, *CYP2C19* (major), *CYP2D6* (minor), *CYP3A4* (major), *CYP3A5* (major), *UGT1A4* ** Inhibitor:** *CYP2C9* (moderate), *CYP2C19* (strong), *CYP2D6* (moderate), *GSTP1*, *SLC6A4* **Transporter genes:** *SLC6A4* **Pleiotropic genes:** *FABP1, PTGS2*
	**IUPAC Name**: (3-{14-chloro-2-azatricyclo[9.4.0.0³,⁸]pentadeca-1(11),3,5,7,12,14-hexaen-2-yl}propyl)dimethylamine	
	**Molecular Formula:** C_19_H_24_Cl_2_N_2_	
	**Molecular Weight:** 351.31326 g/mol	
	**Category:** Tricyclics	
	**Mechanism:** It is a strong, but not completely selective serotonin reuptake inhibitor; as its active main metabolite desmethyclomipramine acts preferably as an inhibitor of noradrenaline reuptake. α1-receptor blockage and β-down-regulation have been noted and most likely play a role in its short term effects. A blockade of sodium-channels and NDMA-receptors	
	**Effect:** Serotonin uptake inhibition; Antidepressant action; Anti-anxiety activity; Antiobsessional effects; Analgesic effects	
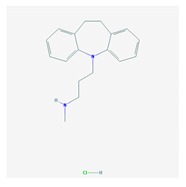	**Name: Desipramine Hydrochloride**; Norpramin; Desipramine Hcl; DMI hydrochloride; Pertofrane; Pertofran	**Pathogenic genes:***ABCB1*, *CRHR1*, *CRHR2*, *FKBP5*, *HTR1A*, *IL1B*, *NR3C1*, *NTRK2*, *PDE5A*, *SLC6A4*, *TBX21* **Mechanistic genes:***ADCY1*, *ADRA1A*, *ADRBs*, *CHRMs*, *HTR1A*, *IFNA1*, *PDE1C*, *PSMD9*, *PRKCSH*, *STAT3* **Metabolic genes:** ** Substrate:***CYP1A2* (minor), *CYP2C9*, *CYP2D6* (major) ** Inhibitor:** *ABCB1, CYP2A6* (moderate), *CYP2B6* (moderate), *CYP2C19* (moderate), *CYP2D6* (moderate), *CYP2E1* (weak), *CYP3A4* (moderate), *SLC6A2*, *SLC22A3* **Transporter genes:** *ABCB1*, *SLC6A2*, *SLC6A3*, *SLC6A4*, *SLC22A3* **Pleiotropic genes:** *NTRK2*, *FOS*
	**IUPAC Name**: (3-{2-azatricyclo[9.4.0.0³,^8^]pentadeca-1(15),3,5,7,11,13-hexaen-2-yl}propyl)(methyl)amine	
	**Molecular Formula:** C_18_H_23_ClN_2_	
	**Molecular Weight:** 302.84162 g/mol	
	**Category:** Tricyclics	
	**Mechanism:** Increases the synaptic concentration of norepinephrine in the CNS by inhibition of its reuptake by the presynaptic neuronal membrane. Additional receptor effects including desensitization of adenyl cyclase, down-regulation of β-adrenergic receptors, and down-regulation of serotonin receptors	
	**Effect:** Enzyme inhibition; Adrenergic uptake inhibition; Antidepressant action; Analgesic activity	
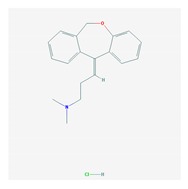	**Name: Doxepin Hydrochloride**; Silenor; Adapin; Novoxapin; Toruan; Curatin	**Pathogenic genes:***ABCB1*, *SLC6A4* **Mechanistic genes:***ADRBs*, *CHRMs*, *HRH1*, *HRH2*, *HTRs* **Metabolic genes:** ** Substrate:***CYP1A1* (minor), *CYP1A2* (minor), *CYP2C9* (minor), *CYP2C19* (major), *CYP2D6* (major), *CYP3A4/5* (minor), *GSTP1*, *UGT1A3*, *UGT1A4* ** Inhibitor:** *CYP2C19* (strong), *CYP2D6* (moderate) **Transporter genes:** *ABCB1*, *KCNH2*, *SLC6A2*, *SLC6A4*
	**IUPAC Name**: dimethyl(3-{9-oxatricyclo[9.4.0.0³,⁸]pentadeca-1(15),3,5,7,11,13-hexaen-2-ylidene}propyl)amine	
	**Molecular Formula:** C_19_H_22_ClNO	
	**Molecular Weight:** 315.83708 g/mol	
	**Category:** Tricyclics	
	**Mechanism:** It increases the synaptic concentration of serotonin and norepinephrine in the CNS by inhibition of their reuptake by the presynaptic neuronal membrane	
	**Effect:** Adrenergic uptake inhibition; Histamine Antagonism; Antidepressant action; Analgesic effects; Pruritus reduction	
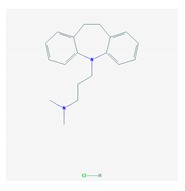	**Name: Imipramine Hydrochloride;** Tofranil; Imipramine Hcl; 113-52-0; Chimoreptin; Feinalmin	**Pathogenic genes:***ABCB1*, *BDNF*, *HTR2A*, *SLC6A4* **Mechanistic genes:***ADRB2*, *DRD2*, *CHRMs*, *HTR2A*, *SCNs* **Metabolic genes:** ** Substrate:***CYP1A2* (minor), *CYP2B6* (minor), *CYP2C19* (major), *CYP2D6* (major), *CYP3A4* (minor), *CYP3A7*, *GSTP1*, *UGT1A3*, *UGT1A4*, *UGT2B10* ** Inhibitor:** *CYP1A2* (weak), *CYP2C9* (moderate), *CYP2C19* (weak), *CYP2D6* (moderate), *CYP2E1* (weak), *CYP3A4* (moderate), *FMO1*, *SLC22A2*, *SLC22A3* **Transporter genes:** *ABCB1*, *SLC6A2*, *SLC6A4*, *SLC22A2*, *SLC22A3* **Pleiotropic genes:** *ADRB2*, *BDNF*, *FABP1*, *FOS*, *ORM1*
	**IUPAC Name**: (3-{2-azatricyclo[9.4.0.0³,⁸]pentadeca-1(15),3,5,7,11,13-hexaen-2-yl}propyl)dimethylamine	
	**Molecular Formula:** C_19_H_25_ClN_2_	
	**Molecular Weight:** 316.8682 g/mol	
	**Category:** Tricyclics	
	**Mechanism:** It binds the sodium-dependent serotonin transporter and sodium-dependent norepinephrine transporter preventing or reducing the reuptake of norepinephrine and serotonin by nerve cells. It causes down-regulation of cerebral cortical beta-adrenergic receptors	
	**Effect:** Adrenergic uptake inhibition; Antidepressant action; Antienuretic effects; Analgesic activity; attention enhancer	
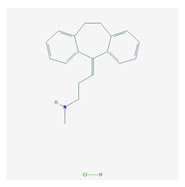	**Name: Nortriptyline Hydrochloride;** Pamelor; Allegron; Altilev; Nortrilen; 894-71-3	**Pathogenic genes:***ABCB1*, *GNB3*, *HTR1B*, *NR3C1*, *SLC6A4* **Mechanistic genes:***ADCY1*, *ADRA2s*, *ADRBs*, *GNB3*, *HRH1*, *HTRs* **Metabolic genes:** ** Substrate:***CYP1A2* (minor), *CYP2C19* (minor), *CYP2D6* (major), *CYP3A4* (minor), *UGTs* ** Inhibitor:** *CYP2C8* (moderate), *CYP2C9* (moderate), *CYP2C19* (moderate), *CYP2D6* (weak), *CYP2E1* (weak), *CYP3A4* (moderate) **Transporter genes:** *ABCB1*, *SLC6A2*, *SLC6A4* **Pleiotropic genes:** *HTR1B*
	**IUPAC Name**: methyl(3-{tricyclo[9.4.0.0³,⁸]pentadeca-1(15),3,5,7,11,13-hexaen-2-ylidene}propyl)amine	
	**Molecular Formula:** C_19_H_22_ClN	
	**Molecular Weight:** 299.83768 g/mol	
	**Category:** Tricyclics	
	**Mechanism:** Inhibits the reuptake of the neurotransmitter serotonin at the neuronal membrane or acts at beta-adrenergic receptors. It has additional receptor effects including desensitization of adenyl cyclase, down-regulation of β-adrenergic receptors, and down-regulation of serotonin receptors	
	**Effect:** Adrenergic uptake inhibitor; Antidepressant agent; Analgesic activity; Hypno-sedative activity	
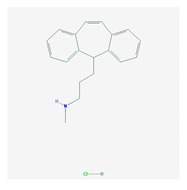	**Name: Protriptyline Hydrochloride**	**Mechanistic genes:***SLC6A2*, *SLC6A4* **Metabolic genes:** ** Substrate:***CYP1A2* (minor), *CYP2C19* (minor), *CYP2D6* (major), *CYP3A4* (minor) ** Inhibitor:** *CYP1A2* (moderate), *CYP2C9* (moderate), *CYP2C19* (moderate), *CYP2D6* (moderate), *CYP3A4* (moderate) **Transporter genes:** *SLC6A2*, *SLC6A4* **Pleiotropic genes:** *ADRA1A*, *GNAS*, *ITGB3*
	**IUPAC Name**: methyl(3-{tricyclo[9.4.0.0³,⁸]pentadeca-1(15),3,5,7,9,11,13-heptaen-2-yl}propyl)amine	
	**Molecular Formula:** C_19_H_22_ClN	
	**Molecular Weight:** 299.83768 g/mol	
	**Category:** Tricyclics	
	**Mechanism:** Increases synaptic concentration of serotonin and/or norepinephrine in CNS by inhibition of their reuptake by presynaptic neuronal membrane	
	**Effect:** Adrenergic uptake inhibitor; Antidepressant agent; Analgesic activity; Anti-migraine effect	
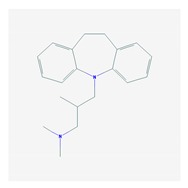	**Name: Trimipramine;** Sapilent; Surmontil; Beta-Methylimipramine; Trimeprimina	**Pathogenic genes:***ABCB1*, *SLC6A4* **Mechanistic genes:***SLC6A2*, *SLC6A4*, *SLC22A1*, *SLC22A2* **Metabolic genes:** ** Substrate:***CYP2C19* (major), *CYP2D6* (major), *CYP3A4/5* (major) ** Inhibitor:** *ABCB1* **Transporter genes:** *SLC6A2*, *SLC6A4*, *SLC22A1*, *SLC22A2*
	**IUPAC Name**: (3-{2-azatricyclo[9.4.0.0³,^8^]pentadeca-1(15),3,5,7,11,13-hexaen-2-yl}-2-methylpropyl)dimethylamine	
	**Molecular Formula:** C_20_H_26_N_2_	
	**Molecular Weight:** 294.43384 g/mol	
	**Category:** Tricyclics	
	**Mechanism:** Increases synaptic concentration of serotonin and/or norepinephrine in CNS by inhibition of their reuptake by presynaptic neuronal membrane	
	**Effect:** Adrenergic uptake inhibition; Antidepressant action; Antihistaminic activity; Sedative effect	
**Selective Serotonin and Norepinephrine Reuptake Inhibitors (SSNRI)**		
**Drug**	**Properties**	**Pharmacogenetics**
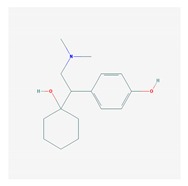	**Name: Desvenlafaxine;** O-Desmethylvenlafaxine; 93413-62-8; 4-(2-(Dimethylamino)-1-(1-hydroxycyclohexyl)ethyl)phenol; 4-[2-(Dimethylamino)-1-(1-hydroxycyclohexyl)ethyl]phenol; Desvenlafaxine (INN)	**Pathogenic genes:***ABCB1*, *SLC6A4* **Mechanistic genes:***HTR1A*, *SLC6A2*, *SLC6A3*, *SLC6A4* **Metabolic genes:** ** Substrate:***CYP3A4* (minor), *UGTs* ** Inhibitor:** *CYP2D6* (weak), *SLC6A2*, *SLC6A4* **Transporter genes:** *ABCB1*, *SLC6A2*, *SLC6A4*
	**IUPAC Name**: 4-[2-(dimethylamino)-1-(1-hydroxycyclohexyl)ethyl]phenol	
	**Molecular Formula:** C_16_H_25_NO_2_	
	**Molecular Weight:** 263.3752 g/mol	
	**Mechanism:** It is a potent and selective serotonin and norepinephrine reuptake inhibitor	
	**Effect:** Serotonin uptake inhibition; Norepinephrine uptake inhibition; Antidepressant activity	
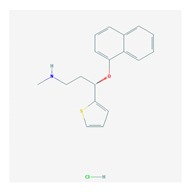	**Name: Duloxetine Hydrochloride**; 136434-34-9; Duloxetine HCl; Cymbalta; (S)-N-Methyl-3-(naphthalen-1-yloxy)-3-(thiophen-2-yl)propan-1-amine hydrochloride; (S)-Duloxetine HCl	**Pathogenic genes:***ABCB1*, *SLC6A4* **Mechanistic genes:***COMT*, *HTR1A*, *SLC6A2*, *SLC6A4* **Metabolic genes:** ** Substrate:***CYP1A2* (major), *CYP2D6* (major) ** Inhibitor:** *ABCB1, CYP1A2* (moderate), *CYP2B6* (moderate), *CYP2C19* (moderate), *CYP2D6* (moderate), *CYP3A4/5* (moderate), *SLC6A2*, *SLC6A4* **Transporter genes:** *ABCB1*, *SLC6A2*, *SLC6A4*
	**IUPAC Name**: methyl[(3S)-3-(naphthalen-1-yloxy)-3-(thiophen-2-yl)propyl]amine	
	**Molecular Formula:** C_18_H_20_ClNOS	
	**Molecular Weight:** 333.8755 g/mol	
	**Mechanism:** It is a selective serotonin- and norepinephrine-reuptake inhibitor and a weak inhibitor of dopamine reuptake	
	**Effect:** Antidepressant activity; Anti-anxiety activity; Serotonin uptake inhibition; Norepinephrine uptake inhibition; Anti-Fibromyalgia agent; Analgesic activity; Urinary continence improvement	
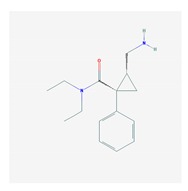	**Name: Levomilnacipran**; UNII-UGM0326TXX; UGM0326TXX; Fetzima; (1S,2R)-2-(aminomethyl)-N,N-diethyl-1-phenylcyclopropane-1-carboxamide; F2695	**Pathogenic genes:***SLC6A4* **Mechanistic genes:***HCRTR1, HCRTR2, HDC, HRH1, SLC6A2, SLC6A4* **Metabolic genes:** ** Substrate:***ABCB1* (minor), *CYP2C19* (minor), *CYP2C8* (minor), *CYP2D6* (minor), *CYP2J2* (minor), *CYP3A4* (major) **Transporter genes:** *SLC6A2*, *SLC6A4*
	**IUPAC Name**: (1S,2R)-2-(aminomethyl)-N,N-diethyl-1-phenylcyclopropane-1-carboxamide	
	**Molecular Formula:** C_15_H_22_N_2_O	
	**Molecular Weight:** 246.34798 g/mol	
	**Mechanism:** Potentiation of serotonin and norephinephrine in the central nervous system through inhibition of reuptake at serotonin and norepinephrine transporters	
	**Effect:** Serotonin uptake inhibition; Norepinephrine uptake inhibition; Antidepressant activity	
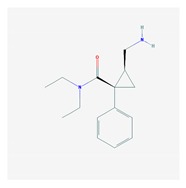	**Name: Milnacipran Hydrochloride**; Toledomin; Midalcipran; Ixel; Savella; Milnacipranum	**Pathogenic genes:***BDNF* **Mechanistic genes:***ADRA2A*, *BDNF*, *SLC6A2*, *SLC6A4* **Metabolic genes:** ** Substrate:***COMT*, *CYP1A2* (minor), *CYP2A6* (minor), *CYP2B6* (minor), *CYP2C8*, *CYP2C9* (minor), *CYP2C19* (minor), *CYP2D6* (minor), *CYP2E1* (minor), *CYP3A4/5* (minor), *UGTs* ** Inhibitor:** *CYP3A4/5* (moderate) ** Inducer:** *CYP1A2*, *CYP2B6*, *CYP2C8*, *CYP2C9*, *CYP2C19*, *CYP3A4/5* **Transporter genes:** *SLC6A2*, *SLC6A4*
	**IUPAC Name**: (1R,2S)-2-(aminomethyl)-N,N-diethyl-1-phenylcyclopropane-1-carboxamide	
	**Molecular Formula:** C_15_H_22_N_2_O	
	**Molecular Weight:** 246.34798 g/mol	
	**Mechanism:** It is a potent inhibitor of neuronal norepinephrine and serotonin reuptake. It inhibits norepinephrine uptake with approximately 3-fold higher potency in vitro than serotonin without directly affecting the uptake of dopamine or other neurotransmitters	
	**Effect:** Analgesic action; Anti-fibromyalgia action; Serotonin uptake inhibition; Adrenergic uptake inhibition; Antidepressant activity	
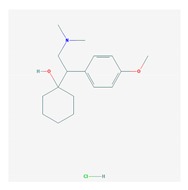	**Name: Venlafaxine Hydrochloride**; 99300-78-4; VENLAFAXINE HCl; Effexor XR; Dobupal; Trevilor	**Pathogenic genes:***ABCB1, BDNF, CREB1, FKBP5, HTR1A, HTR2A, NR3C1, SLC6A3, SLC6A4, TPH2* **Mechanistic genes:***BDNF, FKBP5* **Metabolic genes:** ** Substrate**: *ABCB1, CYP2C9* (minor)*, CYP2C19* (minor)*, CYP2D6* (major)*, CYP3A4* (major) ** Inhibitor**: *ABCB1, CYP1A2* (weak)*, CYP2B6* (weak)*, CYP2D6* (weak)*, CYP3A4* (weak)*, SLC6A2, SLC6A3, SLC6A4* **Transporter genes**: *ABCB1, ABCC1, ABCG2, SLC6A2, SLC6A3, SLC6A4* **Pleiotropic genes**: *DRD2, HTR2A, TPH2*
	**IUPAC Name**: 1-[2-(dimethylamino)-1-(4-methoxyphenyl)ethyl]cyclohexan-1-ol	
	**Molecular Formula:** C_17_H_28_ClNO_2_	
	**Molecular Weight:** 313.86272 g/mol	
	**Mechanism:** Active metabolite, O-desmethylvenlafaxine (ODV), are potent inhibitors of neuronal serotonin and norepinephrine reuptake and weak inhibitors of dopamine reuptake	
	**Effect:** Serotonin uptake inhibition; Norepinephrine uptake inhibition; Antidepressant activity; Anti-anxiety activity, Analgesic effects	
**Selective Serotonin Reuptake Inhibitors (SSRI)**		
**Drug**	**Properties**	**Pharmacogenetics**
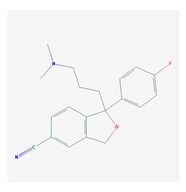	**Name: Citalopram Hydrobromide**; Nitalapram; Cipram; Celexa; Celapram; Ciprapine	**Pathogenic genes:***ABCB1*, *BDNF*, *CREB1*, *CRHR1*, *CRHR2*, *FKBP5*, *GRIA3*, *GRIK2*, *GRIK4*, *GSK3B*, *HTR1A*, *HTR1B*, *HTR2A*, *MAOA*, *SLC6A4*, *TPH1*, *TPH2* **Mechanistic genes:***ADRs*, *CHRMs*, *DRDs*, *FKBP5*, *GABRs*, *GRIK4*, *HRHs*, *HTR1A*, *HTR1B*, *HTR1D*, *HTR2A*, *SLC6A4*, *TPH1* **Metabolic genes:** ** Substrate:***ABCC1*, *COMT*, *CYP2C19* (major), *CYP2D6* (minor), *CYP3A4* (major), *CYP3A5* ** Inhibitor:** *ABCB1*, *CYP1A2* (weak), *CYP2B6* (weak), *CYP2C19* (weak), *CYP2D6* (weak), *MAOA*, *MAOB* **Transporter genes:** *ABCB1*, *SLC6A4* **Pleiotropic genes:** *BDNF*
	**IUPAC Name**: 1-[3-(dimethylamino)propyl]-1-(4-fluorophenyl)-1,3-dihydro-2-benzofuran-5-carbonitrile	
	**Molecular Formula:** C_20_H_21_FN_2_O	
	**Molecular Weight:** 324.391943 g/mol	
	**Mechanism:** Selectively inhibits serotonin reuptake in the presynaptic neurons and has minimal effects on norepinephrine or dopamine	
	**Effect:** Serotonin uptake inhibition; Serotonergic neurotransmission enhancer; Antidepressive activity, Agitation reduction, Anti-Anxiety activity	
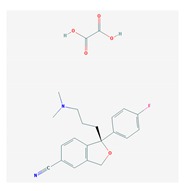	**Name: Escitalopram oxalate**; Lexapro; Cipralex; 219861-08-2; UNII-5U85DBW7LO; Esertia	**Pathogenic genes:***ABCB1*, *CREB1*, *FKBP5*, *GRIA3*, *GRIK2*, *GRIK4*, *NR3C1*, *SLC6A4* **Mechanistic genes:***ADRAs*, *ADRBs*, *DDC*, *DRDs*, *CHRMs*, *GABRs*, *HRHs*, *HTRs*, *IL6* **Metabolic genes:** **Substrate:***ABCB1*, *CYP2C9* (minor), *CYP2C19* (major), *CYP2D6* (major), *CYP3A4* (major) **Inhibitor:** *ABCB1*, *CYP1A2* (weak), *CYP2C9* (weak), *CYP2C19* (weak), *CYP2D6* (moderate), *CYP2E1* (weak), *CYP3A4* (weak), *SLC6A4* **Transporter genes:** *ABCB1*, *SLC6A4* **Pleiotropic genes:** *IL6*
	**IUPAC Name**: (1S)-1-[3-(dimethylamino)propyl]-1-(4-fluorophenyl)-1,3-dihydro-2-benzofuran-5-carbonitrile	
	**Molecular Formula:** C_22_H_23_FN_2_O_5_	
	**Molecular Weight:** 414.426823 g/mol	
	**Mechanism:** Inhibits the reuptake of serotonin with little to no effect on norepinephrine or dopamine reuptake. It has very low affinity for 5-HT_1-7_, α - and β-adrenergic, D_1-5_, H_1-3_, M_1-5_, and benzodiazepine receptors	
	**Effect:** Serotonin uptake inhibition; Serotonergic neurotransmission enhancer; Antidepressive activity; Anti-anxiety activity	
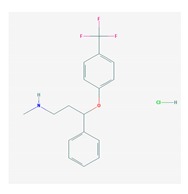	**Name: Fluoxetine Hydrochloride**; Prozac; Fluoxetine Hcl; 59333-67-4; Sarafem; Fluctin	**Pathogenic genes:***ABCB1*, *BDNF*, *CREB1*, *FKBP5*, *GSK3B*, *HTR1A*, *HTR2A*, *MAOA*, *NR3C1*, *NTRK2*, *SLC6A4*, *TBX21*, *TPH1*, *TPH2* **Mechanistic genes:***BDNF*, *CHRMs*, *CREB1*, *DRD3*, *GSK3B*, *HTRs*, *MAOA*, *SLC6A4*, *TPH2* **Metabolic genes:** ** Substrate:***CYP1A2* (major), *CYP2B6* (major), *CYP2C8* (major), *CYP2C9* (major), *CYP2C19* (major), *CYP2D6* (major), *CYP2E1* (minor), *CYP3A4/5* (major), *POR* ** Inhibitor:** *ABCB1*, *CYP1A2* (moderate), *CYP2B6* (weak), *CYP2C8* (moderate), *CYP2C9* (weak), *CYP2C19* (moderate), *CYP2D6* (strong), *CYP3A4* (moderate), *MAOA*, *SLC6A4* **Transporter genes:** *ABCB1*, *KCNH2, SLC6A4* **Pleiotropic genes:** *DRD3*, *FABP1*, *HTR2A*, *IFNA1*, *NTRK2*, *PDE5A*, *TPH1*
	**IUPAC Name**: methyl({3-phenyl-3-[4-(trifluoromethyl)phenoxy]propyl})amine	
	**Molecular Formula:** C_17_H_19_ClF_3_NO	
	**Molecular Weight:** 345.78707 g/mol	
	**Mechanism:** Potentiates serotonergic activity in CNS resulting from its inhibition of CNS neuronal reuptake of serotonin	
	**Effect:** Serotonin uptake inhibition; Serotonin agent; Antidepressive activity; Anti-obsessive activity; Anti-anxiety activity; Anorexigenic effects	
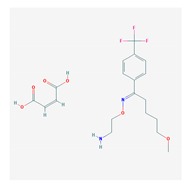	**Name: Fluvoxamine Maleate**; Luvox; 61718-82-9; Fevarin; Faverin; Floxyfral	**Pathogenic genes:***BDNF*, *HTR2A*, *SIGMAR1*, *TPH1* **Mechanistic genes:***BDNF*, *HTRs*, *SLC6A4*, *SIGMAR1* **Metabolic genes:** ** Substrate:***CYP1A2* (major), *CYP2C19* (major), *CYP2D6* (major), *CYP3A4* (major) ** Inhibitor:** *ABCB1*, *CYP1A2* (strong), *CYP2B6* (weak), *CYP2C9* (moderate), *CYP2C19* (moderate), *CYP2D6* (moderate), *CYP3A4* (weak), *MAOA*, *SLC6A4* **Transporter genes:** *ABCB1*, *KCNH2*, *SCL6A4* **Pleiotropic genes:** *CREB1*, *TPH1*
	**IUPAC Name**: (2-aminoethoxy)({5-methoxy-1-[4-(trifluoromethyl)phenyl]pentylidene})amine	
	**Molecular Formula:** C_19_H_25_F_3_N_2_O_6_	
	**Molecular Weight:** 434.40681 g/mol	
	**Mechanism:** Inhibits CNS neuron serotonin uptake	
	**Effect:** Antidepressive activity; Anti-anxiety activity; Serotonin uptake inhibition	
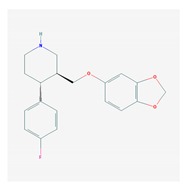	**Name: Paroxetine**; Paxil; Aropax; Paxil CR; Seroxat; Pexeva	**Pathogenic genes:***ABCB1*, *CREB1*, *HTR1B*, *HTR2A*, *HTR3B*, *MAOA*, *SLC6A3*, *SLC6A4*, *TNF*, *TPH1*, *TPH2* **Mechanistic genes:***CREB1*, *HTR2A*, *HTR3A*, *SLC6A4, STAT3*, *TNF* **Metabolic genes:** ** Substrate:***ABCB1*, *COMT*, *CYP1A2* (minor), *CYP2C19* (minor), *CYP2D6* (major), *CYP3A4* (major), *MAOA*, *MAOB* ** Inhibitor:** *ABCB1*, *CYP1A2* (weak), *CYP2B6* (moderate), *CYP2C9* (weak), *CYP2C19* (weak), *CYP2D6* (strong), *CYP3A4* (weak), *SLC6A3*, *SLC6A4* **Transporter genes:** *ABCB1*, *SLC6A3*, *SLC6A4* **Pleiotropic genes:** *HTR1D*, *HTR3C*, *HTR6*, *HTT*, *TPH1*, *TPH2*
	**IUPAC Name**: (3S,4R)-3-[(2H-1,3-benzodioxol-5-yloxy)methyl]-4-(4-fluorophenyl)piperidine	
	**Molecular Formula:** C_19_H_20_FNO_3_	
	**Molecular Weight:** 329.365403 g/mol	
	**Mechanism:** It is an SSRI. Presumably acts by inhibiting serotonin reuptake from brain synapse stimulating its activity in the brain	
	**Effect:** Serotonin uptake inhibition; Serotonergic neurotransmission enhancer; Antidepressant activity; Anti-anxiety activity; Anti-obsessive activity.	
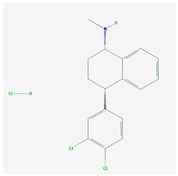	**Name: Sertraline Hydrochloride;** 79559-97-0; Sertraline HCl; Zoloft; Lustral; Gladem	**Pathogenic genes:***ABCB1*, *CREB1*, *GNB3*, *HTR1B*, *MAOA*, *SIGMAR1*, *SLC6A4*, *TNF*, *TPH1*, *TPH2* **Mechanistic genes:***HTR1B*, *HTR1D*, *SIGMAR1*, *SLC6A2*, *SLC6A3*, *SLC6A4*, *TNF* **Metabolic genes:** ** Substrate:***CYP2A6*, *CYP2B6* (minor), *CYP2C9* (minor), *CYP2C19* (major), *CYP2D6* (minor), *CYP3A4* (minor), *MAOA*, *MAOB*, *UGT1A1*, *UGT2B7* ** Inhibitor:** *ABCB1*, *ACHE*, *CYP1A1*, *CYP1A2* (weak), *CYP2B6* (moderate), *CYP2C8* (weak), *CYP2C9* (weak), *CYP2C19* (moderate), *CYP2D6* (moderate), *CYP3A4* (moderate), *SLC6A4* **Transporter genes:** *ABCB1*, *SLC6A2*, *SLC6A3*, *SLC6A4* **Pleiotropic genes:** *FABP1*, *FOS*, *GNB3*, *TPH1*, *TPH2*
	**IUPAC Name**: (1S,4S)-4-(3,4-dichlorophenyl)-N-methyl-1,2,3,4-tetrahydronaphthalen-1-amine	
	**Molecular Formula:** C_17_H_18_Cl_3_N	
	**Molecular Weight:** 342.69052 g/mol	
	**Mechanism:** It has selective inhibitory effects on presynaptic serotonin reuptake and only very weak effects on norepinephrine and dopamine neuronal uptake	
	**Effect:** Serotonin uptake inhibition; Serotonergic neurotransmission enhancer; Antidepressant activity; Anti-anxiety activity; Anti-obsessive activity	
**Serotonin Modulators**		
**Drug**	**Properties**	**Pharmacogenetics**
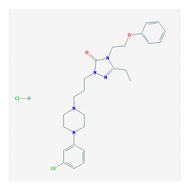	**Name: Nefazodone Hydrochloride**; Serzone; Nefazodone Hcl; Dutonin; 82752-99-6; Menfazon	**Pathogenic genes:***ABCB1*, *HTR1A*, *HTR2A* **Mechanistic genes:***ADRA1A*, *HTR1s*, *HTR2s* **Metabolic genes:** ** Substrate:***CYP2D6* (major), *CYP3A4* (major), *CYP3A5* (major) ** Inhibitor:** *ABCB1*, *ABCC2*, *CYP1A2* (weak), *CYP2B6* (weak), *CYP2C8* (weak), *CYP2D6* (weak), *CYP3A4* (strong), *SLC6A2* **Transporter genes:** *ABCB1*, *ABCC2*, *ABCB11, SLC6A2*
	**IUPAC Name**: 1-{3-[4-(3-chlorophenyl)piperazin-1-yl]propyl}-3-ethyl-4-(2-phenoxyethyl)-4,5-dihydro-1H-1,2,4-triazol-5-one	
	**Molecular Formula:** C_25_H_33_Cl_2_N_5_O_2_	
	**Molecular Weight:** 506.46782 g/mol	
	**Mechanism:** Blocks potently and selectively postsynaptic 5-HT_2A_ receptors and moderately inhibits serotonin and noradrenaline reuptake. Also blocks α1 receptors. Antagonist of adrenoceptors alpha 1 and 5-hydroxytryptamine receptors 2	
	**Effect:** Serotonin uptake inhibition; Noradrenaline uptake inhibition; Alpha-adrenergic antagonism; Antidepressant activity; Muscle relaxation, Sedation	
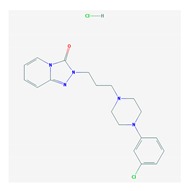	**Name: Trazodone Hydrochloride**; Desyrel; 25332-39-2; Trazodone Hcl; Molipaxin; Trittico	**Pathogenic genes:***ABCB1*, *GNB3*, *HTR1A*, *HTR2A*, *SLC6A4* **Mechanistic genes:***ADRA1s*, *ADRA2s*, *HRH1*, *HTR2A*, *HTR2C* **Metabolic genes:** ** Substrate:***CYP1A2* (minor), *CYP2D6* (minor), *CYP3A4* (major), *GSTs*, *SOD2* ** Inhibitor:** *CYP2D6* (moderate), *CYP3A4* (weak), *SLC6A4* ** Inducer:** *ABCB1* **Transporter genes:** *ABCB1*, *SLC6A4* **Pleiotropic genes:** *GNAS*, *GNB3*, *HTR2A*
	**IUPAC Name**: 2-{3-[4-(3-chlorophenyl)piperazin-1-yl]propyl}-2H,3H-[1,2,4]triazolo[4,3-a]pyridin-3-one	
	**Molecular Formula:** C_19_H_23_Cl_2_N_5_O	
	**Molecular Weight:** 408.32482 g/mol	
	**Mechanism:** Inhibits reuptake of serotonin, causes adrenoreceptor subsensitivity, and induces significant changes in 5-HT presynaptic receptor adrenoreceptors. Also significantly blocks histamine (H_1_) and α_1_-adrenergic receptors	
	**Effect:** Serotonin uptake inhibitor; Anti-anxiety activity; Antidepressant agent; Hypnotic effects	
**Miscellaneous Antidepressants**		
**Drug**	**Properties**	**Pharmacogenetics**
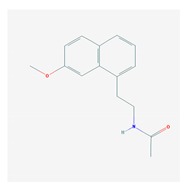	**Name: Agomelatine;** 138112-76-2; Valdoxan; Thymanax; Melitor; N-(2-(7-Methoxynaphthalen-1-yl)ethyl)acetamide	**Mechanistic genes:***HTR2C, MTNR1A, MTNR1B* **Metabolic genes:** ** Substrate:***CYP1A1* (major), *CYP1A2* (major), *CYP2C9* (minor), *CYP2C19* (minor)
	**IUPAC Name**: N-[2-(7-methoxynaphthalen-1-yl)ethyl]acetamide	
	**Molecular Formula:** C_15_H_17_NO_2_	
	**Molecular Weight:** 243.30098 g/mol	
	**Category:** Melatonergic agonist and 5-HT2C antagonists	
	**Mechanism:** It behaves as an agonist at melatonin receptors and as an antagonist at serotonin (5-HT)(2C) receptors	
	**Effect:** Norepinephrine-dopamine disinhibitor; Antidepressant activity; Anti-anxiety activity; Sleep induction; Circadian rhythms resynchronization; Psychological excitement reduction	
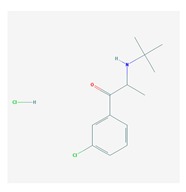	**Name: Bupropion Hydrochloride;** 31677-93-7; Wellbutrin; Zyban; Bupropion Hcl	**Pathogenic genes:***SLC6A3*, *SLC6A4* **Mechanistic genes:***ADRA1A*, *CHRNB2*, *DRD2* **Metabolic genes:** ** Substrate:***COMT*, *CYP1A2* (minor), *CYP2A6* (minor), *CYP2B6* (major) *CYP2C9* (minor), *CYP2C19* (minor), *CYP2D6* (minor), *CYP2E1* (minor), *CYP3A4* (minor) ** Inhibitor:** *CYP2D6* (strong) **Transporter genes:** *SLC6A2*, *SLC6A3*, *SLC6A4* **Pleiotropic genes:** *DRD2*
	**IUPAC Name**: 2-(tert-butylamino)-1-(3-chlorophenyl)propan-1-one	
	**Molecular Formula:** C_13_H_19_Cl_2_NO	
	**Molecular Weight:** 276.20206 g/mol	
	**Category:** Dopamine-Reuptake Inhibitor	
	**Mechanism:** It is a relatively weak inhibitor of the neuronal uptake of norepinephrine and dopamine	
	**Effect:** Dopamine uptake inhibition; Anti-addiction/Substance abuse treatment agent; Smoking cessation enhancer; Antidepressant activity	
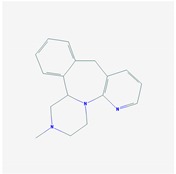	**Name: Mirtazapine;** Zispin; Remergil; Remeron; 6-Azamianserin; Remergon	**Pathogenic genes:***ABCB1*, *FKBP5*, *HTR1A*, *HTR2A*, *MAOA*, *SLC6A3*, *SLC6A4*, *TPH2* **Mechanistic genes:***ADRA1s*, *ADRA2A*, *CHRMs, FKBP5*, *HRH1*, *HTR2s, HTR3s* **Metabolic genes:** ** Substrate:***CYP1A2* (major), *CYP2C9* (minor), *CYP2D6* (major), *CYP3A4* (major), *UGT1A1*, *UGT1A3*, *UGT1A4*, *UGT1A6*, *UGT1A7*, *UGT1A9*, *UGT1A10*, *UGT2B7*, *UGT2B15* ** Inhibitor:** *CYP1A2* (weak), *CYP3A4* (weak), *CYP2D6* (weak), *MAOA*, *MAOB* ** Inducer:** *CYP1A2, CYP2D6, CYP3A4* **Transporter genes:** *ABCB1*, *SLC6A3*, *SLC6A4* **Pleiotropic genes:** *TPH2*
	**IUPAC Name**: 5-methyl-2,5,19-triazatetracyclo[13.4.0.0^2^, .0^8^,^13^]nonadeca-1(15),8,10,12,16,18-hexaene	
	**Molecular Formula:** C_17_H_19_N_3_	
	**Molecular Weight:** 265.35286 g/mol	
	**Category:** α2-Adrenergic Antagonist	
	**Mechanism:** It has central presynaptic α_2_-adrenergic antagonist effects, which result in increased release of norepinephrine and serotonin. Also a potent antagonist of 5-HT_2_ and 5-HT_3_ serotonin receptors, H_1_ histamine receptors and a moderate peripheral α_1_-adrenergic and muscarinic antagonist	
	**Effect:** Histamine H1 antagonism; Adrenergic alpha-antagonism; Antidepressant activity; Anxiolytic effects	
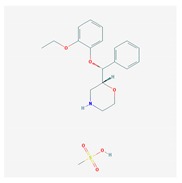	**Name: Reboxetine Mesylate;** Vestra mesylate; Davedax mesylate; Reboxetine mesilate; Edronax; 98769-84-7	**Pathogenic genes:***ABCB1*, *SLC6A3*, *SLC6A4* **Mechanistic genes:***ADRs*, *CHRMs*, *CHRNs*, *HRHs*, *DRDs*, *GH1*, *HTRs*, *POMC*, *PRL* **Metabolic genes:** ** Substrate:***CYP3A4* (major) ** Inhibitor:** *ABCB1*, *CYP2D6* (weak), *CYP3A4* (weak) **Transporter genes:** *ABCB1*, *SLC6A2*, *SLC6A3*, *SLC6A4* **Pleiotropic genes:** *ADRB2*, *POMC*
	**IUPAC Name**: (2S)-2-[(S)-2-ethoxyphenoxy(phenyl)methyl]morpholine	
	**Molecular Formula:** C_20_H_27_NO_6_S	
	**Molecular Weight:** 409.49648 g/mol	
	**Category:** Norepinephrine inhibitor	
	**Mechanism:** It is a highly selective and potent inhibitor of noradrenaline reuptake. Only has weak effect on 5-HT reuptake	
	**Effect:** Adrenergic uptake inhibitor; Antidepressant activity; Anti-anxiety activity; Attention enhancer	

**Table 5:** Pharmacological profile and pharmacogenetics of selected anxiolytics, sedatives, and hypnotics.

**Barbiturates**		
**Drug**	**Properties**	**Pharmacogenetics**
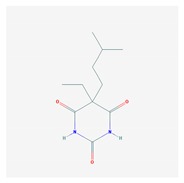	**Name: Amobarbital**; Amylobarbitone; Barbamyl; Pentymal; Amytal; Barbamil	**Pathogenic genes:** *GABRs* **Mechanistic genes:** *CHRNAs, CLCNs, GABRAs, GABRBs, GRIA2, NDUFs* **Metabolic genes:** ** Substrate:** *UGT2B7, UGT2B15* ** Inducer:** *CYP2A6* **Transporter genes:** *CLCNs*
	**IUPAC Name**: 5-ethyl-5-(3-methylbutyl)-1,3-diazinane-2,4,6-trione	
	**Molecular Formula:** C_11_H_18_N_2_O_3_	
	**Molecular Weight:** 226.27222 g/mol	
	**Mechanism:** Interferes with transmission of impulses resulting in an imbalance in central inhibitory and facilitatory mechanisms. Binds to alpha or beta subunits of GABA-A receptor. Decreases input resistance, depresses burst and tonic firing, especially in ventrobasal and intralaminar neurons. Increases burst duration and mean conductance at individual chloride channels and the amplitude and decay time of inhibitory postsynaptic currents. Blocks the AMPA receptor and appears to bind neuronal nicotinic acetylcholine receptors.	
	**Effect:** Hypnotic activity; Sedation; GABA modulator	
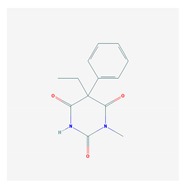	**Name: Mephobarbital; **Mebaral; Mephobarbitone; Enphenemal; Prominal; Methylphenobarbitone	**Pathogenic genes:***GABRA1, GABRB3, GABRG2, GABRD* **Mechanistic genes:***GABRAs, GABRBs, GABRD, GABRE, GABRGs, GABRP, GABRQ, GABRRs* **Metabolic genes:** ** Substrate:***CYP2B6* (minor), *CYP2C9* (minor), *CYP2C19* (major), *CYP2D6* ** Inhibitor:** *CYP2C19 (*weak*)* ** Inducer:** *CYP2A6*
	**IUPAC Name**: 5-ethyl-1-methyl-5-phenyl-1,3-diazinane-2,4,6-trione	
	**Molecular Formula:** C_13_H_14_N_2_O_3_	
	**Molecular Weight:** 246.26186 g/mol	
	**Mechanism:** Increases seizure threshold in motor cortex. Depresses monosynaptic and polysynaptic transmission in CNS.	
	**Effect:** Hypnotic activity; Sedation; Anti-anxiety; GABA Modulator, Anticonvulsant	
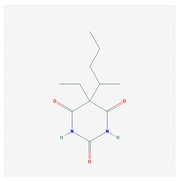	**Name: Pentobarbital**; Pentobarbitone; Nembutal; Mebubarbital; Mebumal; Ethamina	**Pathogenic genes:***BDNF, IL6, TNF* **Mechanistic genes:***GABRA6, GABRB3, GRIA1, GRIA2, NPY* **Metabolic genes:** ** Substrate:***CYP1A2, CYP2B6, CYP2D6, PTGS1, PTGS2* Inducer: *CYP2A6, CYP3A4* **Transporter genes:** *KCNE1, KCNH2, NR1I2, NR1I3,* **Pleiotropic genes:** *APP, BDNF, BLK, CNR1, CRHR1, FOS, ICAM1, IL1B, IL6, KRAS, NPPA, NPY, TNF, TNFRSF1A*
	**IUPAC Name**: 5-ethyl-5-pentan-2-yl-1,3-diazinane-2,4,6-trione	
	**Molecular Formula:** C_11_H_18_N_2_O_3_	
	**Molecular Weight:** 226.27222 g/mol	
	**Mechanism:** Prolongs the post-synaptic inhibitory effect of GABA in the thalamus. Inhibits the excitatory AMPA-type glutamate receptors, resulting in a profound suppression of glutamatergic neurotransmission	
	**Effect:** Hypnotic activity; Sedation; GABA Modulator; Anticonvulsant; Anesthesia (Adjuvant)	
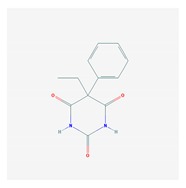	**Name: Phenobarbital**; Luminal; Phenobarbitone; Phenobarbitol; Gardenal; Phenemal	**Pathogenic genes:***CASR, GABRA6, LEP, PSEN1, PTGS2, TGFB1, TNF* **Mechanistic genes:***CACNs, CLCNs, GABRAs, GRIAs, GSTA1, NR1I3* **Metabolic genes:** ** Substrate:***ABCC2, ACSL4, CBR3, CES1, CES2, COMT, CYP1A2* (minor), *CYP2C9* (minor), *CYP2C19* (major), *CYP2E1* (minor), *CYP4B1, CYP7A1, EPHX1, GSTM1, GSTP1, GSTT1, NNMT, NQO1, TBXAS1, TPMT* ** Inhibitor:** *ABCB1, ABCC2, ABCC4, ABCG2, CYP2C19* (strong), *CYP2J2* (strong), *CYP27A1* (strong), *SLC10A1, SULT1A1* ** Inducer:** *ABCB1, ABCC1, ABCC2, ABCC3, ABCC4, CYP1A1, CYP1A2, CYP2A6, CYP2B6, CYP2C8, CYP2C9, CYP2C18, CYP2C19, CYP2D6, CYP2E1, CYP3A4, CYP4A11, CYP4F3, CYP7A1, CYP8B1, CYP24A1, SCN1A, SLC22A1, SLCO1B1, SULT1C2, SULT2A1, TPMT, UGT1A1, UGT1A4, UGT1A7, UGT1A9, UGT2B7* **Transporter genes:** *ABCB1, ABCB11, ABCC1, ABCC2, ABCC3, ABCC4, ABCC6, ABCG2, SCN1A, SLC22A1, SLCO1B1, SLCO1B3, SLCO2B1, SLC10A1* **Pleiotropic genes:** *ACHE, ADIPOQ, AHR, APOA1, APOE, CAT, CBS, CCND1, CDA, CXCR2, DDC, DPP4, FGB, FKBP5, GH1, GNAS, GRK5, HLA-B, HNF4A, IGF1, IL1B, IL6, LEP, LEPR, LIPC, MET, MTNR1A, NR1I2, NR3C1, PPARGC1A, PRKAB1, PSEN2, RB1, RXRA, TGFB1, TNF*
	**IUPAC Name**: 5-ethyl-5-phenyl-1,3-diazinane-2,4,6-trione	
	**Molecular Formula:** C_12_H_12_N_2_O_3_	
	**Molecular Weight:** 232.23528 g/mol	
	**Mechanism:** It is a barbituric acid derivative that acts as a nonselective central nervous system depressant. It potentiates action on GABA-A receptors, and modulates chloride currents through receptor channels. It also inhibits glutamate induced depolarizations	
	**Effect:** Hypnotic activity; Sedation; GABA Modulator; Anticonvulsant; Carcinogen; Central Nervous System Depressant; Excitatory Amino Acid Antagonist; Respiratory depression (dose-dependent)	
**Benzodiazepines**		
**Drug**	**Properties**	**Pharmacogenetics**
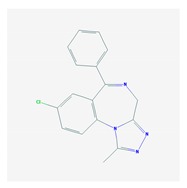	**Name: Alprazolam; **Xanax; Trankimazin; Tafil; Cassadan; Tranquinal	**Pathogenic genes**: *GABRs* **Mechanistic genes**: *CLCNs, GABRs* **Metabolic genes**: ** Substrate:** CYP1A1 (minor), CYP1A2 (minor), CYP2B6, CYP2C9, CYP2C19 (minor), CYP2D6 (minor), CYP3A4/5 (major) **Transporter genes:** CLCNs
	**IUPAC Name**: 8-chloro-1-methyl-6-phenyl-4H-[1,2,4]triazolo[4,3-a][1,4]benzodiazepine	
	**Molecular Formula:** C_17_H_13_ClN_4_	
	**Molecular Weight:** 308.76492 g/mol	
	**Mechanism:** Binds to the GABA benzodiazepine receptor complex, particularly in the limbic system and reticular formation. The inhibitory effect of GABA on neuronal excitability increases the neuronal membrane permeability to chloride ions resulting in hyperpolarization and stabilization	
	**Effect:** Anti-Anxiety Agent; Hypnotic activity; Sedation; GABA Modulator	
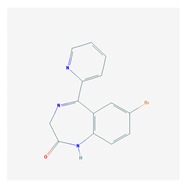	**Name: Bromazepam**; Compedium; Creosedin; Lectopam; Lexaurin; Lexilium	**Pathogenic genes:***GABRs* **Mechanistic genes:***CLCNs, GABRs* **Metabolic genes:** ** Substrate:***CYP1A2, CYP2C19, CYP3A4* (major), *CYP3A5* ** Inhibitor:** *CYP2E1* (weak) **Transporter genes:** *ABCB1, CLCNs*
	**IUPAC Name**: 7-bromo-5-pyridin-2-yl-1,3-dihydro-1,4-benzodiazepin-2-one	
	**Molecular Formula:** C_14_H_10_BrN_3_O	
	**Molecular Weight:** 316.1527 g/mol	
	**Mechanism:** Binds to stereospecific benzodiazepine receptors on the postsynaptic GABA neuron in the Central Nervous System (limbic system, reticular formation). Enhances the inhibitory GABA-effect on neuronal excitability by increasing cellular permeability to chloride ions, resulting in hyperpolarization (a less excitable state) and stabilization of cellular membrane	
	**Effect:** Anti-Anxiety Agent; GABA Modulator; Skeletal muscle relaxant	
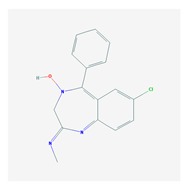	**Name: Chlordiazepoxide**; Chlozepid; Elenium; Chlorodiazepoxide; Methaminodiazepoxide; Chlordiazepoxid	**Pathogenic genes:***BDNF, CLCNs, GABRs* **Mechanistic genes:***CLCNs, GABRs* **Metabolic genes:** ** Substrate:***CYP2D6, CYP3A4* (major) **Transporter genes:** *ABCB1, CLCNs* **Pleiotropic genes:** *BDNF*
	**IUPAC Name**: 7-chloro-4-hydroxy-N-methyl-5-phenyl-3H-1,4-benzodiazepin-2-imine	
	**Molecular Formula:** C_16_H_14_ClN_3_O	
	**Molecular Weight:** 299.75486 g/mol	
	**Mechanism:** Binds to the GABA receptor type A and increases the inhibitory effect of GABA on neuronal excitability by enhancing neuronal membrane permeability to chloride ions, thus resulting in hyperpolarization and stabilization	
	**Effect:** Sedation; Anti-Anxiety Agent; GABA Modulator, Skeletal muscle relaxant, Anticonvulsant; Amnesic properties, Anesthesia (Adjuvant)	
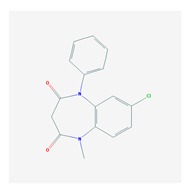	**Name: Clobazam**; Urbanyl; Chlorepin; Clorepin; Frisium; Clobazamum	**Pathogenic genes:***GABRA1*, *GABRB3*, *GABRG2*, *GABRD* **Mechanistic genes:***CLCNs*, *GABRs* **Metabolic genes:** ** Substrate:***CYP2B6* (minor), *CYP2C18* (minor), *CYP2C19* (major), *CYP3A4* (major), *CYP3A5* (major) **Transporter genes:** *CLCNs*
	**IUPAC Name**: 7-chloro-1-methyl-5-phenyl-1,5-benzodiazepine-2,4-dione	
	**Molecular Formula:** C_16_H_13_ClN_2_O_2_	
	**Molecular Weight:** 300.73962 g/mol	
	**Mechanism:** Binds to stereospecific receptors on the postsynaptic GABA neuron at several sites within the CNS (limbic system, reticular formation). Enhances the inhibitory effect of GABA on neuronal excitability by increasing neuronal membrane permeability to chloride ions, which results in hyperpolarization (a less excitable state) and stabilization	
	**Effect:** Anti-Anxiety Agent; GABA Modulator; Anticonvulsant	
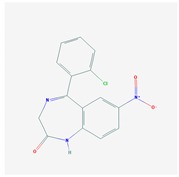	**Name: Clonazepam**; Rivotril; Antelepsin; Iktorivil; Chlonazepam; Cloazepam	**Pathogenic genes:***GABRA1* **Mechanistic genes:***GABRAs*Metabolic genes: **Substrate:***CYP3A4/5* (major), *NAT2*
	**IUPAC Name**: 5-(2-chlorophenyl)-7-nitro-1,3-dihydro-1,4-benzodiazepin-2-one	
	**Molecular Formula:** C_15_H_10_ClN_3_O_3_	
	**Molecular Weight:** 315.7112 g/mol	
	**Mechanism:** Enhance the activity of γ-aminobutyric acid (GABA). Suppresses the spike-and-wave discharge in absence seizures by depressing nerve transmission in the motor cortex. Depresses all levels of the CNS, including the limbic and reticular formation, by binding to the benzodiazepine site on the GABA receptor complex and modulating GABA	
	**Effect:** Anticonvulsant; GABA Modulator; Antipanic effect	
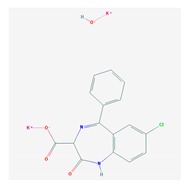	**Name: Clorazepate Dipotassium**; Tranxilium; Tranxene; Abbott-35616; 4306 CB; Dipotassium clorazepate	**Pathogenic genes:***GABRs* **Mechanistic genes:***CLCNs, GABRs* **Metabolic genes:** ** Substrate:***CYP3A4* (major), *CYP3A5* (major) **Transporter genes:** *CLCNs*
	**IUPAC Name**: dipotassium;7-chloro-2-oxo-5-phenyl-1,3-dihydro-1,4-benzodiazepine-3-carboxylate;hydroxide	
	**Molecular Formula:** C_16_H_11_ClK_2_N_2_O_4_	
	**Molecular Weight:** 408.91914 g/mol	
	**Mechanism:** Depresses all levels of the CNS, including the limbic and reticular formation, by binding to the benzodiazepine site on the γ-aminobutyric acid (GABA) receptor complex and modulating GABA, resulting in an increased neuronal membrane permeability to chloride ions which produces a hyperpolarization and stabilization	
	**Effect:** Skeletal muscle relaxant. Anti-Anxiety Agent; GABA Modulator; Anticonvulsant	
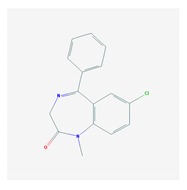	**Name: Diazepam**; Valium; Ansiolisina; Diazemuls; Apaurin; Faustan	**Pathogenic genes:***BDNF, CNR1, GABRD* **Mechanistic genes:***ACHE, BCHE, BDNF, CACNA1C, CHRMs, GABRs, TSPO* **Metabolic genes:** ** Substrate:***CYP1A2* (minor), *CYP2B6* (minor), *CYP2C9* (minor), *CYP2C19* (minor), *CYP3A4/5* (major), *UGTs* ** Inhibitor:** *CYP2C19* (weak), *CYP3A4* (weak), *UGT2B7* **Transporter genes:** *ABCB1* **Pleiotropic genes:** *FOS, IL6, SPG7*
	**IUPAC Name**: 7-chloro-1-methyl-5-phenyl-3H-1,4-benzodiazepin-2-one	
	**Molecular Formula:** C_16_H_13_ClN_2_O	
	**Molecular Weight:** 284.74022 g/mol	
	**Mechanism:** Binds to stereospecific benzodiazepine receptors on the postsynaptic GABA neuron at several sites within the CNS. Enhancement of the inhibitory effect of GABA on neuronal excitability results by increased neuronal membrane permeability to chloride ions, thus resulting in hyperpolarization and stabilization. It antagonizes with translocator protein	
	**Effect:** Sedation; Anti-Anxiety Agent; GABA Modulator, Skeletal muscle relaxant, Anticonvulsant; Amnesic properties, Anesthesia; Antiemetics	
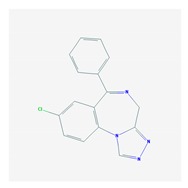	**Name: Estazolam**; Eurodin; Nuctalon; ProSom; Esilgan; Julodin	**Pathogenic genes:***GABRB3* **Mechanistic genes:***CLCNs, GABRAs, GABRBs, GABRD, GABRE, GABRGs, GABRP, GABRQ, GABRRs, TSPO* **Metabolic genes:** **Substrate:***CYP1A2, CYP2A6, CYP2C9, CYP2C19, CYP2D6, CYP2E1, CYP3A4 (*major*), CYP3A5 (*major*)* **Transporter genes:***CLCNs*
	**IUPAC Name**: 8-chloro-6-phenyl-4H-[1,2,4]triazolo[4,3-a][1,4]benzodiazepine	
	**Molecular Formula:** C_16_H_11_ClN_4_	
	**Molecular Weight:** 294.73834 g/mol	
	**Mechanism:** Binds to stereospecific benzodiazepine receptors on the postsynaptic GABA neuron at several sites within the CNS, including the limbic system, reticular formation. Enhancement of the inhibitory effect of GABA on neuronal excitability results by increased neuronal membrane permeability to chloride ions. This shift in chloride ions results in hyperpolarization (a less excitable state) and stabilization	
	**Effect:** Anti-Anxiety Agent; GABA Modulator; Hypnotic activity; Sedation; Skeletal muscle relaxant; Anticonvulsant	
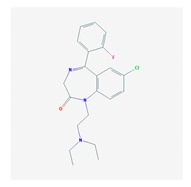	**Name: Flurazepam**; Dalmane; Dalmadorm; Flurazepamum; Felmane; Noctosom	**Pathogenic genes:***GABRB3* **Mechanistic genes:***CACNA1C, CLCNs, GABRs* **Metabolic genes:** ** Substrate:***CYP3A4* (major)*, CYP3A5* (major)*, MAO* ** Inhibitor:** *ABCB1, CYP2E1* (weak) **Transporter genes:** *ABCB1, CACNA1C, CLCNs*
	**IUPAC Name**: 7-chloro-1-[2-(diethylamino)ethyl]-5-(2-fluorophenyl)-3H-1,4-benzodiazepin-2-one	
	**Molecular Formula:** C_21_H_23_ClFN_3_O	
	**Molecular Weight:** 387.878223 g/mol	
	**Mechanism:** Binds to stereospecific benzodiazepine receptors on the postsynaptic GABA neuron at several sites within the CNS, including the limbic system, reticular formation. Enhancement of inhibitory effect of GABA on neuronal excitability results by increased neuronal membrane permeability to chloride ions	
	**Effect:** Anti-Anxiety Agent; GABA Modulator; Sedation	
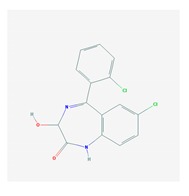	**Name: Lorazepam**; Ativan; Temesta; O-Chloroxazepam; O-Chlorooxazepam; Almazine	**Pathogenic genes:***GABRs* **Mechanistic genes:***CLCNs, GABRs, TSPO* **Metabolic genes:** ** Substrate:***CYP3A4* (minor)*, UGT1A1, UGT1A3, UGT1A4, UGT1A6, UGT1A7, UGT1A9, UGT1A10, UGT2B4, UGT2B7, UGT2B15* **Transporter genes:** *CLCNs*
	**IUPAC Name**: 7-chloro-5-(2-chlorophenyl)-3-hydroxy-1,3-dihydro-1,4-benzodiazepin-2-one	
	**Molecular Formula:** C_15_H_10_Cl_2_N_2_O_2_	
	**Molecular Weight:** 321.1581 g/mol	
	**Mechanism:** Binds to stereospecific benzodiazepine receptors on postsynaptic GABA neuron at several sites within CNS, including limbic system, reticular formation. Enhancement of inhibitory effect of GABA on neuronal excitability results by increased neuronal membrane permeability to chloride ions. This shift in chloride ions results in hyperpolarization (a less excitable state) and stabilization. Binds to GABAA receptors enhancing the effects of GABA by increasing GABA affinity for its receptor	
	**Effect:** Anti-Anxiety Agent; GABA Modulator; Sedation; Skeletal muscle relaxant; Anticonvulsant. Antiemetic; Hypnotic activity; Preanesthetic agent	
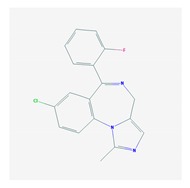	**Name: Midazolam**; Versed; Dormicum; Midazolamum; 59467-70-8; Midazolamum	**Mechanistic genes:***CLCNs, GABRs* **Metabolic genes:** ** Substrate:***CYP1A2, CYP2A6, CYP2B6* (minor)*, CYP2C19* (minor)*, CYP2D6, CYP2E1, CYP3A4* (major)*, CYP3A5* (major)*, CYP3A7, UGT1A4, UGT2B7, UGT2B10* ** Inhibitor:** *CYP2C8* (weak)*, CYP2C9* (weak), *CYP3A4* (strong) Inducer: *CYP3A4* **Transporter genes:** *ABCB1, CLCNs, NR1I2*
	**IUPAC Name**: 8-chloro-6-(2-fluorophenyl)-1-methyl-4H-imidazo[1,5-a][1,4]benzodiazepine	
	**Molecular Formula:** C_18_H_13_ClFN_3_	
	**Molecular Weight:** 325.767323 g/mol	
	**Mechanism:** Binds to stereospecific benzodiazepine receptors on postsynaptic GABA neuron. Enhancement of inhibitory effect of GABA on neuronal excitability results by increased neuronal membrane permeability to chloride ions. This shift in chloride ions results in hyperpolarization (a less excitable state) and stabilization	
	**Effect:** Sedation; Hypnotic activity; Anti-Anxiety Agent; GABA Modulator, Skeletal muscle relaxant; Amnestic properties; Anesthesia; Preanesthetia	
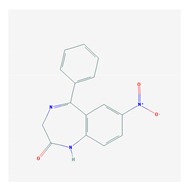	**Name: Nitrazepam**; Benzalin; Mogadon; Remnos; Nitrados; Imeson	**Pathogenic genes:***GABRA1, GABRB3, GABRG2, GABRD* **Mechanistic genes:***CLCNs, GABRs, GLRs* **Metabolic genes:** ** Substrate:***CYP3A4* (major) **Transporter genes:** *CLCNs*
	**IUPAC Name**: 7-nitro-5-phenyl-1,3-dihydro-1,4-benzodiazepin-2-one	
	**Molecular Formula:** C_15_H_11_N_3_O_3_	
	**Molecular Weight:** 281.26614 g/mol	
	**Mechanism:** Binds to stereospecific benzodiazepine receptors on postsynaptic GABA neuron at CNS (limbic system, reticular formation). Enhances inhibitory effect of GABA on neuronal excitability by increasing neuronal membrane permeability to chloride ions	
	**Effect:** Sedation; Hypnotic activity; Anticonvulsant; Anti-Anxiety Agent; GABA Modulator	
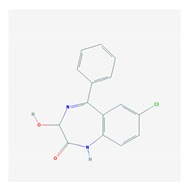	**Name: Oxazepam**; Adumbran; Tazepam; Serax; Anxiolit; Praxiten	**Pathogenic genes:***GABRs* **Mechanistic genes:***CLCNs, GABRs* **Metabolic genes:** ** Substrate:***CYP2D6, UGT2B7, UGT2B15* ** Inhibitor:***UGT2B7* Inducer: *CYP1A2* **Transporter genes:** *CLCNs*
	**IUPAC Name**: 7-chloro-3-hydroxy-5-phenyl-1,3-dihydro-1,4-benzodiazepin-2-one	
	**Molecular Formula:** C_15_H_11_ClN_2_O_2_	
	**Molecular Weight:** 286.71304 g/mol	
	**Mechanism:** Effects appear to be mediated through inhibitory neurotransmitter GABA; site and mechanism of action within the CNS appear to involve macromolecular complex (GABA_A_-receptor-chloride ionophore complex) which includes GABA_A_ receptors, high-affinity benzodiazepine receptors, and chloride channels. Enhancement of inhibitory effect of GABA on neuronal excitability results by increased neuronal membrane permeability to chloride ions. This shift in chloride ions results in hyperpolarization (less excitable state) and stabilization	
	**Effect:** Sedation; GABA Modulator; Anti-Anxiety Agent; Anti-Alcohol withdrawal agent	
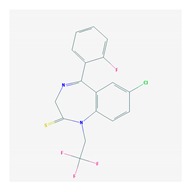	**Name: Quazepam**; Doral; Dormalin; Prosedar; Oniria; Quazium	**Pathogenic genes:***GABRB3* **Mechanistic genes:***CLCNs, GABRs* **Metabolic genes:** ** Substrate:***CYP2C9* (minor)*, CYP2C19* (minor)*, CYP3A4* (major)*, FMO1, FMO3* **Transporter genes:** *CLCNs*
	**IUPAC Name**: 7-chloro-5-(2-fluorophenyl)-1-(2,2,2-trifluoroethyl)-3H-1,4-benzodiazepine-2-thione	
	**Molecular Formula:** C_17_H_11_ClF_4_N_2_S	
	**Molecular Weight:** 386.794253 g/mol	
	**Mechanism:** Binds to stereospecific benzodiazepine receptors on postsynaptic GABA neuron at several sites within CNS (limbic system, reticular formation). Enhances inhibitory effect of GABA on neuronal excitability by increasing neuronal membrane permeability to chloride ions, resulting in hyperpolarization and stabilization	
	**Effect:** Hypnotic activity; Sedation; GABA Modulator; Anti-Anxiety Agent	
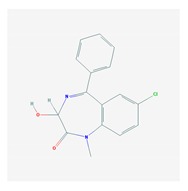	**Name: Temazepam**; Euhypnos; Restoril; Hydroxydiazepam; Methyloxazepam; Crisonar	**Pathogenic genes:***GABRB3* **Mechanistic genes:***CLCNs, GABRs, TSPO* **Metabolic genes:** ** Substrate:***CYP2B6* (major)*, CYP2C9* (major)*, CYP2C19* (major)*, CYP3A4* (major)*, UGT1A1, UGT1A3, UGT1A4, UGT1A6, UGT1A9, UGT1A10, UGT2B7, UGT2B15* **Inhibitor:** *UGT1A3, UGT2B7* **Transporter genes:** *CLCNs*
	**IUPAC Name**: 7-chloro-3-hydroxy-1-methyl-5-phenyl-3H-1,4-benzodiazepin-2-one	
	**Molecular Formula:** C_16_H_13_ClN_2_O_2_	
	**Molecular Weight:** 300.73962 g/mol	
	**Mechanism:** A short half-life benzodiazepine. Binds to stereospecific benzodiazepine receptors on postsynaptic GABA neuron at several sites within CNS, including limbic system. Enhances inhibitory effect of GABA on neuronal excitability results by increased neuronal membrane permeability to chloride ions. This shift in chloride ions results in hyperpolarization (a less excitable state) and stabilization. It antagonizes with translocator protein	
	**Effect:** Hypnotic activity; Sedation; GABA Modulator; Anti-Anxiety Agent; Antidepressant activity; Anticonvulsant	
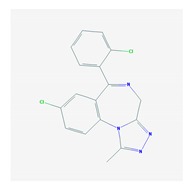	**Name: Triazolam**; Halcion; Songar; Clorazolam; Novidorm; Trilam	**Pathogenic genes:***GABRB3* **Mechanistic genes:***CLCNs, GABRs, TSPO* **Metabolic genes:** ** Substrate:***CYP3A4* (major)*, CYP3A5* (major) ** Inhibitor:** *CYP2C8* (weak)*, CYP2C9* (weak) **Transporter genes:** *CLCNs*
	**IUPAC Name**: 8-chloro-6-(2-chlorophenyl)-1-methyl-4H-[1,2,4]triazolo[4,3-a][1,4]benzodiazepine	
	**Molecular Formula:** C_17_H_12_Cl_2_N_4_	
	**Molecular Weight:** 343.20998 g/mol	
	**Mechanism:** Binds to stereospecific benzodiazepine receptors on postsynaptic GABA neuron at several sites within CNS, including limbic system, reticular formation. Enhancement of inhibitory effect of GABA on neuronal excitability results by increased neuronal membrane permeability to chloride ions. This shift in chloride ions results in hyperpolarization (less excitable state) and stabilization.	
	**Effect:** Sedation; GABA Modulator; Anesthesia (Adjuvant)	
**Miscellaneous**		
**Drug**	**Properties**	**Pharmacogenetics**
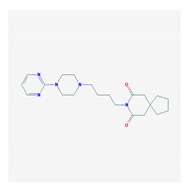	**Name: Buspirone**; Ansial; Buspirona; Buspironum; Bespar; Anxiron	**Mechanistic genes:***DRD2, HTR1A, HTR2A, HTR3s* **Metabolic genes:** ** Substrate:***CYP2D6* (minor)*, CYP3A4* (major), *CYP3A5* (minor) Inducer: *CYP3A4* **Pleiotropic genes:** *DRD2, HTR2A*
	**IUPAC Name**: 8-[4-(4-pyrimidin-2-ylpiperazin-1-yl)butyl]-8-azaspiro[4.5]decane-7,9-dione	
	**Molecular Formula:** C_21_H_31_N_5_O_2_	
	**Molecular Weight:** 385.50314 g/mol	
	**Mechanism:** Decreases the spontaneous firing of serotonin-containing neurons in the CNS by selectively binding to and acting as agonist at presynaptic CNS serotonin 5-HT_1A_ receptors. Possesses partial agonist activity (mixed agonist/antagonist) at postsynaptic 5-HT_2A_ receptors. Does not bind to benzodiazepine-GABA receptors. Binds to dopamine D_2_ receptors	
	**Effect:** Serotonin receptor agonist; Anti-Anxiety Agent	
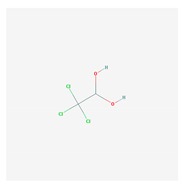	**Name: Chloral Hydrate**; Noctec; Tosyl; 302-17-0; 2,2,2-Trichloroethane-1,1-diol; Trichloroacetaldehyde hydrate	**Pathogenic genes:** *GABRAs* **Mechanistic genes:** *GABRAs; GLRs; HTR3s* **Pleiotropic genes:** *FOS, IL1B, IL6*
	**IUPAC Name**: 2,2,2-trichloroethane-1,1-diol	
	**Molecular Formula:** C_2_H_3_Cl_3_O_2_	
	**Molecular Weight:** 165.40302 g/mol	
	**Mechanism:** It is converted to the active compound trichloroethanol by hepatic alcohol dehydrogenase. The agent interacts with various neurotransmitter-operated ion channels, thereby enhancing gamma-aminobutyric acid (GABA)-A receptor mediated chloride currents and inhibiting amino acid receptor-activated ion currents. Enhances the agonistic effects of glycine receptors, inhibits AMPA-induced calcium influx in cortical neurons, and facilitates 5-HT 3 receptor-mediated currents in ganglionic neurons. Overall, this results in a depressive effect on the central nervous system	
	**Effect:** Hypnotic activity; Sedation; Anticonvulsant; Anesthesia; Analgesic activity; GABA Modulator	
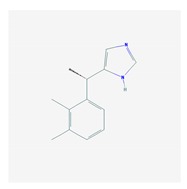	**Name: Dexmedetomidine Hydrochloride**; Dexmedetomidina; Dexmedetomidinum; MPV 1440; Precedex; CHEBI:4466	**Mechanistic genes:***ADRA2A, ADRA2B, ADRA2C* **Metabolic genes:** ** Substrate:***CYP2A6* (major), *UGT1A4, UGT2B10* ** Inhibitor:** *CYP1A2* (weak), *CYP2C9* (weak)*, CYP2D6* (strong)*, CYP3A4* (weak) ** Inducer:** *ABCC2*, *CYP7A1*, *CYP27A1* **Transporter genes:** *ABCC2*
	**IUPAC Name**: 5-[(1S)-1-(2,3-dimethylphenyl)ethyl]-1H-imidazole	
	**Molecular Formula:** C_13_H_16_N_2_	
	**Molecular Weight:** 200.27954 g/mol	
	**Mechanism:** Binds to the presynaptic alpha-2 adrenoceptors and inhibits the release of norepinephrine, therefore, terminate the propagation of pain signals. Activation of the postsynaptic alpha-2 adrenoceptors inhibits the sympathetic activity	
	**Effect:** α2-Adrenergic Agonist; Anesthesia; Sedation; Analgesia	
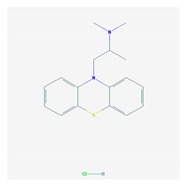	**Name: Promethazine Hydrochloride; **Phenergan; Promethazine Hcl; 58-33-3; Fenergan; Atosil	**Mechanistic genes:***ADRA1A*, *CHRM2*, *DRD2*, *HRH1* **Metabolic genes:** ** Substrate:***CYP2B6* (major), *CYP2D6* (major) ** Inhibitor:** *CYP2C9* (weak), *CYP2D6* (weak) **Transporter genes:** *ABCB1*
	**IUPAC Name**: N,N-dimethyl-1-phenothiazin-10-ylpropan-2-amine;hydrochloride	
	**Molecular Formula:** C_17_H_21_ClN_2_S	
	**Molecular Weight:** 320.88004 g/mol	
	**Mechanism:** Blocks postsynaptic mesolimbic dopaminergic receptors in brain. Exhibits strong α-adrenergic-blocking effect and depresses release of hypothalamic and hypophyseal hormones. Competes with histamine for H1-receptor. Reduces stimuli to brainstem reticular system. The relief of nausea is related to central anticholinergic actions	
	**Effect:** Sedation; Anti-Allergic Agent; Antiemetic; Antipruritic; Histamine H1 Antagonist	
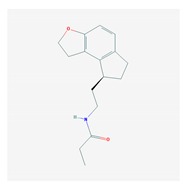	**Name: Ramelteon**; Rozerem; 196597-26-9; TAK-375; Rozerem	**Pathogenic genes:***MTNR1A, MTNR1B* **Mechanistic genes:***MTNR1A, MTNR1B* **Metabolic genes:** ** Substrate:***CYP1A2* (major)*, CYP2C19* (minor)*, CYP3A4* (minor)
	**IUPAC Name**: N-[2-[(8S)-2,6,7,8-tetrahydro-1H-cyclopenta[e][1]benzofuran-8-yl]ethyl]propanamide	
	**Molecular Formula:** C_16_H_21_NO_2_	
	**Molecular Weight:** 259.34344 g/mol	
	**Mechanism:** Potent, selective agonist of melatonin receptors MT_1_ and MT_2_ (with little affinity for MT_3_) within suprachiasmic nucleus of hypothalamus	
	**Effect:** Melatonin receptor agonist; Hyptonic activity; circadian rhythm regulation	
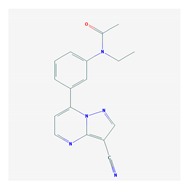	**Name: Zaleplon**; Sonata; 151319-34-5; CL-284846; Zerene; CL 284846	**Mechanistic genes:***GABRA1* **Metabolic genes:** ** Substrate:***AOX1, CYP3A4* (major), *CYP2D6, POR* **Pleiotropic genes:** *HRH1*
	**IUPAC Name**: N-[3-(3-cyanopyrazolo[1,5-a]pyrimidin-7-yl)phenyl]-N-ethylacetamide	
	**Molecular Formula:** C_17_H_15_N_5_O	
	**Molecular Weight:** 305.3339 g/mol	
	**Mechanism:** Interacts with benzodiazepine GABA receptor complex. Nonclinical studies have shown that it binds selectively to brain ω_1_ receptor situated on α-subunit of GABA-A receptor complex	
	**Effect:** Hypnotic activity; Sedation; Skeletal muscle relaxant; Anti-anxiety agent; Anticonvulsant; GABA Modulator	
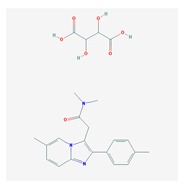	**Name: Zolpidem Tartrate**; 99294-93-6; schembl40721; MLS001401453; Bio-0153; chembl1723343	**Mechanistic genes:***CLCNs, GABRA1, TSPO* **Metabolic genes:** ** Substrate:***CYP1A2* (minor)*, CYP2C9* (minor)*, CYP2C19* (minor)*, CYP2D6* (minor)*, CYP3A4* (major) ** Inhibitor:** *CYP3A4* (strong) **Transporter genes:** *CLCNs, NR1I2*
	**IUPAC Name**: 2,3-dihydroxybutanedioic acid;N,N-dimethyl-2-[6-methyl-2-(4-methylphenyl)imidazo[1,2-a]pyridin-3-yl]acetamide	
	**Molecular Formula:** C_23_H_27_N_3_O_7_	
	**Molecular Weight:** 457.47638 g/mol	
	**Mechanism:** Enhances activity of inhibitory neurotransmitter, GABA, via selective agonism at benzodiazepine-1 (BZ1) receptor. Result is increased chloride conductance, neuronal hyperpolarization, inhibition of action potential, and decrease in neuronal excitability	
	**Effect:** Central nervous system depression; GABA-A receptor agonist; Hypnotic activity; Sedation	
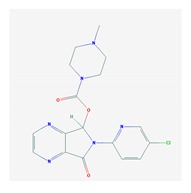	**Name: Zopiclone**; Imovane; Zimovane; Amoban; (+-)-Zopiclone; 43200-80-2	**Mechanistic genes:***CLCNs, GABRAs* **Metabolic genes:** ** Substrate:***CYP2C8* (major), *CYP2C9* (major), *CYP2C19* (minor), *CYP2D6*, *CYP3A4* (major) **Transporter genes:** *CLCNs*
	**IUPAC Name**: [6-(5-chloropyridin-2-yl)-5-oxo-7H-pyrrolo[3,4-b]pyrazin-7-yl] 4-methylpiperazine-1-carboxylate	
	**Molecular Formula:** C_17_H_17_ClN_6_O_3_	
	**Molecular Weight:** 388.80828 g/mol	
	**Mechanism:** Reduces sleep latency, increases duration of sleep, and decreases number of nocturnal awakenings. Binds to the benzodiazepine receptor complex and modulates the GABABZ receptor chloride channel macromolecular complex. Acts on α1, α2, α3 and α5 GABAA containing receptors as a full agonist causing an enhancement of the inhibitory actions of GABA.	
	**Effect:** Hypnotic activity; Sedation	

**Table 6:** Pharmacological properties and pharmacogenetics of selected antiepileptic drugs.

Antiepileptics		
Drug	Properties	Pharmacogenetics
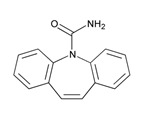	**Name: Carbamazepine**	**Mechanistic genes:***EPHX1,HSPA1L, MTHFR, SCN1A* **Metabolic genes:** ** Substrate:***CYP1A2, CYP2A6, CYP2B6, CYP2C8, CYP2C19, CYP2E1, CYP3A4, CYP3A5, CYP3A7, GSTM1, GSTT1, UGT2B7* ** Inhibitor:***CYP1A2* Inducer: *ABCB1, ABCB4, ABCC2, ABCG2, CYP1A1, CYP1A2, CYP2A6, CYP2B6; CYP2C8; CYP2C9; CYP2C19; CYP2D6, CYP3A4, GSTA1, SULT1A1, UGT1A4* **Transporter genes:** *ABCB1, ABCB4, ABCC2* **Pleiotropic genes:** *HLA-A, HLA-B, IL6*
	**IUPAC Name**: 5H-Dibenz[b,f]azepine-5-carboxamide	
	**Molecular Formula:** C_15_H_12_N_2_O	
	**Molecular Weight:** 236.27 g/mol	
	**Mechanism:** The anticonvulsant activity of carbamazepine, like phenytoin, principally involves limitation of seizure propagation by reduction of post-tetanic potentiation of synaptic transmission. Carbamazepine has only slight analgesic properties. Carbamazepine appears to provide relief of pain in trigeminal neuralgia by reducing synaptic transmission within the trigeminal nucleus. The drug has also demonstrated sedative, anticholinergic, antidepressant, muscle relaxant, antiarrhythmic, antidiuretic, and neuromuscular transmission-inhibitory actions. May depress activity in the nucleus ventralis of the thalamus or decrease synaptic transmission or decrease summation of temporal stimulation leading to neural discharge by limiting influx of sodium ions across cell membrane or other unknown mechanism. May decrease the turnover of γ- aminobutyric acid (GABA). Stimulates the release of ADH and potentiates its action in promoting reabsorption of water. Chemically related to tricyclic antidepressants.	
	**Effect:** Anticonvulsants, Miscellaneous. Antimanic Agents	
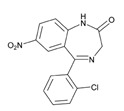	**Name: Clonazepam**	**Mechanistic genes:** *ALB, GABA-A* **Metabolic genes:** ** Substrate:** *CYP2E1, CYP3A4, NAT2*
	**IUPAC Name**: 2H-1,4-Benzodiazepin-2-one, 5-(2-chlorophenyl)-1,3-dihydro-7-nitro-; 5-(o-Chlorophenyl)-1,3-dihydro-7-nitro-2H-1,4-benzodiazepin-2-one	
	**Molecular Formula:** C_15_H_10_ClN_3_O_3_	
	**Molecular Weight:** 315.71 g/mol	
	**Mechanism:** Exact mechanism of anticonvulsant, sedative, and antipanic effects is unknown; however, mechanism appears to be related to the drug’s ability to enhance the activity of γ-aminobutyric acid (GABA). Suppresses the spike-and-wave discharge in absence seizures by depressing nerve transmission in the motor cortex. Depresses all levels of the CNS, including the limbic and reticular formation, by binding to the benzodiazepine site on the GABA receptor complex and modulating GABA	
	**Effect:** Anxiolytics, Sedatives, and Hypnotics; Benzodiazepines. Anticonvulsants; Benzodiazepines	
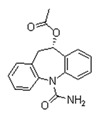	**Name: Eslicarbazepine**	**Metabolic genes:** ** Substrate:** *ABCB1, UGT1A1, UGT1A4, UGT1A9, UGT2B7* ** Inhibitor:** *CYP2C19* ** Inducer:** *CYP3A4, UGT1A4, UGT1A9, UGT2B4, UGT2B7, UGT2B17* **Pleiotropic genes:** *HLA-B*
	**IUPAC Name**: [S-(-)-10-acetoxy-10,11-dihydro-5H-dibenz[b,f]azepine-5-carboxamide]; BIA 2-093	
	**Molecular Formula:** C_17_H_16_N_2_O_3_	
	**Molecular Weight:** 296.32 g/mol	
	**Mechanism:** The precise mechanisms of action of eslicarbazepine acetate are unknown. However, in vitro electrophysiological studies indicate that both eslicarbazepine acetate and its metabolites stabilize the inactivated state of voltage-gated sodium channels, preventing their return to the activated state and thereby sustaining repetitive neuronal firing	
	**Effect:** Anticonvulsants, Miscellaneous. Antiepileptics, Carboxamide Derivatives	
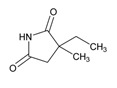	**Name: Ethosuximide**	**Mechanistic genes:** *CACNA1G* **Metabolic genes:** ** Substrate:** *CYP2E1, CYP3A4, CYP3A5*
	**IUPAC Name**: 2,5-Pyrrolidinedione, 3-ethyl-3-methyl-, (±)-; (±)-2-Ethyl-2-methylsuccinimide	
	**Molecular Formula:** C_7_H_11_NO_2_	
	**Molecular Weight:** 141.17 g/mol	
	**Mechanism:** A succinimide-derivative anticonvulsant. Exact mechanism of anticonvulsant action unknown. Increases seizure threshold in cortex and basal ganglia and reduces synaptic response to low-frequency repetitive stimulation. Suppresses paroxysmal spike and wave activity of the EEG associated with lapses of consciousness common in absence seizures	
	**Effect:** Anticonvulsants; Succinimides	
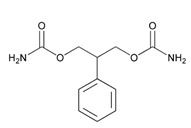	**Name: Felbamate**	**Mechanistic genes:** *GABR, GRIN1, GRIN2B* **Metabolic genes:** ** Substrate:** *CYP2E1, CYP3A4, CYP3A5* ** Inhibitor:** *CYP2C19* ** Inducer:** *CYP3A4* **Transporter genes:** *ABCB1*
	**IUPAC Name**: 2-Phenyl-1,3-propanediol dicarbamate	
	**Molecular Formula:** C_11_H_14_N_2_O_4_	
	**Molecular Weight:** 238.24 g/mol	
	**Mechanism:** Felbamate, a dicarbamate, is an anticonvulsant agent. Exact mechanism of action unknown, but it is suggested that it increases seizure threshold and reduces seizure spread. *In vitro* studies indicate that felbamate has weak inhibitory effects on binding at GABA receptors and benzodiazepine receptors. The monocarbamate, p-hydroxy, and 2-hydroxy metabolites of felbamate appear to contribute little, if any, to the anticonvulsant action of the drug	
	**Effect:** Anticonvulsants	
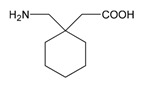	**Name: Gabapentin**	**Mechanistic genes:** *GABRR1, GABRR2, KCNH2, SCN2A* **Metabolic genes:** ** Inhibitor:** *CYP2A6* **Transporter genes:** *ABCB1, SLC22A4*
	**IUPAC Name**: 2-[1-(aminomethyl) cyclohexyl] acetic acid	
	**Molecular Formula:** C_9_H_17_NO_2_.	
	**Molecular Weight:** 171.24 g/mol	
	**Mechanism:** Gabapentin is an anticonvulsant agent structurally related to the inhibitory CNS neurotransmitter GABA	
	**Effect:** Anticonvulsants, Analgesics and Antipyretics	
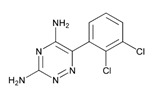	**Name: Lamotrigine**	**Mechanistic genes:** *ADORA1, ADORA2A, ADRA1A, ADRA2A, ADRB1, CACNA1E, CHRM1, CHRM2, CHRM3, CHRM4, CHRM5, DRD1, DRD2, GABRA1, GABRA2, GABRA3, GABRA5, GABRA6, GABRB1, GABRB2, GABRB3, GABRG1, GABRG2, GABRG3, GABRD, GABRE, GABRP, GABRR1, GABRR2, GABRR3, GABRQ, HRH1, HTR2A, HTR3A, OPRK1, SCN2A* **Metabolic genes:** ** Substrate:** *DHFR, UGT1A1, UGT1A3, UGT1A4, UGT2A7* **Transporter genes:** *ABCB1, SLC22A2* **Pleiotropic genes:** *HLA-B*
	**IUPAC Name**: 1,2,4-Triazine-3,5-diamine, 6-(2,3-dichlorophenyl)-; (2) 3,5-Diamino-6-(2,3-dichlorophenyl)-as-triazine	
	**Molecular Formula:** C_9_H_7_Cl_2_N_5_	
	**Molecular Weight:** 256.09 g/mol	
	**Mechanism:** Possibly involves inhibition of voltage-sensitive sodium channels, which stabilizes neuronal membranes and consequently modulates release of excitatory amino acid neurotransmitters (e.g. glutamate, aspartate) which play a role in generation and spread of epileptic seizures	
	**Effect:** Anticonvulsants, Miscellaneous	
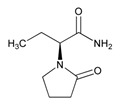	**Name: Levetiracetam**	**Metabolic genes:** ** Substrate:** *CYP2D6* **Transporter genes:** *ABCB1*
	**IUPAC Name**: 1-Pyrrolidineacetamide, α-ethyl-2-oxo-, (α S)-; (2)(-)-(S) - α-Ethyl-2-oxo-1-pyrrolidineacetamide	
	**Molecular Formula:** C_8_H_14_N_2_O_2_	
	**Molecular Weight:** 170.21 g/mol	
	**Mechanism:** The precise mechanism by which levetiracetam exerts its antiepileptic effect is unknown and does not appear to derive from any interaction with known mechanisms involved in inhibitory and excitatory neurotransmission	
	**Effect:** Anticonvulsants	
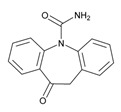	**Name: Oxcarbazepine**	**Mechanistic genes:** *SCN2A* **Metabolic genes:** ** Substrate:** *CYP3A4, UGT1A1, UGT1A3, UGT1A4, UGT1A6, UGT1A7, UGT1A9, UGT1A10, UGT2B7, UGT2B15* ** Inhibitor:** *CYP2C19* ** Inducer:** *ABCB1, CYP3A4, CYP3A5*
	**IUPAC Name**: 5H-Dibenz[b,f]azepine-5-carboxamide, 10,11-dihydro-10-oxo-; 10,11-Dihydro-10-oxo-5H-dibenz[b,f]azepine-5-carboxamide	
	**Molecular Formula:** C_15_H_12_N_2_O_2_	
	**Molecular Weight:** 252.27 g/mol	
	**Mechanism:** Pharmacological activity results from both oxcarbazepine and its monohydroxy metabolite (MHD). Oxcarbazepine and MHD block voltage-sensitive sodium channels, stabilizing hyperexcited neuronal membranes, inhibiting repetitive firing, and decreasing propagation of synaptic impulses. These actions are believed to prevent spread of seizures. Oxcarbazepine and MHD also increase potassium conductance and modulate activity of high-voltage activated calcium channels. Protects against electrically induced tonic extension seizures and, to a lesser degree, chemically-induced clonic seizures. May abolish or reduce frequency of chronically recurring focal seizures	
	**Effect:** Anticonvulsants	
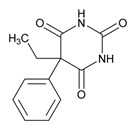	**Name: Phenobarbital**	**Mechanistic genes:** *COMT, GABRA1, CHRNA4, CHRNA7, GRIA2, GRIK2, GRIN1, GRIN2A, GRIN2B, GRIN2C, GRIN2D, GRIN3A, GRIN3B, NR1I2* **Metabolic genes:** ** Substrate:** *CYP1A1, CYP1A2, CYP1B1, CYP2A6, CYP2B6, CYP2C8, CYP2C9, CYP2C18, CYP2C19, CYP2E1, CYP3A4, CYP3A5, CYP3A7, CYP4A11, CYP4B1, EPHX1, UGT1A1* ** Inhibitor:** *CYP2C19, CYP2J2, CYP27A1, SLC10A1, SULT1A1* **Transporter genes:** *ABCB1, ABCB11, ABCC1, ABCC2, ABCC3, SLCO2A1*
	**IUPAC Name**: 2,4,6(1H,3H,5H)-Pyrimidinetrione, 5-ethyl-5-phenyl-; (2) 5-Ethyl-5-phenylbarbituric acid	
	**Molecular Formula:** C_12_H_12_N_2_O_3_	
	**Molecular Weight:** 232.24 g/mol	
	**Mechanism:** Long-acting barbiturate with sedative, hypnotic, and anticonvulsant properties. Barbiturates depress sensory cortex, decrease motor activity, alter cerebellar function, and produce drowsiness, sedation, and hypnosis. In high doses, barbiturates exhibit anticonvulsant activity. They also produce dose-dependent respiratory depression	
	**Effect:** Anticonvulsants; Barbiturates. Anxiolytics, Sedatives, and Hypnotics; Barbiturates	
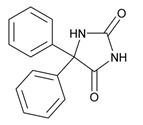	**Name: Phenytoin**	**Mechanistic genes:** *NR1I2, SCN1A, SCN1B, SCN3A, SCN5A* **Metabolic genes:** ** Substrate:** *CYP2B6, CYP2C8, CYP2C9, CYP2C18, CYP2C19, CYP2D6, CYP3A4, CYP3A5, CYP3A7, CYP11B1, EPHX1, UGT1A1, UGT1A6, UGT1A9* ** Inhibitor:** *SCN2A, SULT1A1* **Transporter genes:** *ALB, ABCB1, ABCC2, SLCO1B1, SLCO1C1* **Pleiotropic genes:** *HLA-B*
	**IUPAC Name**: 5,5-Diphenylhydantoin	
	**Molecular Formula:** C_15_H_12_N_2_O_2_	
	**Molecular Weight:** 252.27 g/mol	
	**Mechanism:** Stabilizes neuronal membranes and decreases seizure activity by increasing efflux or decreasing influx of sodium ions across cell membranes in motor cortex during generation of nerve impulses. Prolongs effective refractory period and suppresses ventricular pacemaker automaticity, shortens action potential in heart	
	**Effect:** Class Ib Antiarrhythmics. Anticonvulsants; Hydantoins	
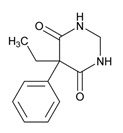	**Name: Primidone**	**Mechanistic genes:** *GABRAs* **Metabolic genes:** ** Inducer:** *CYP1A2, CYP2B6, CYP2C8, CYP2C9, CYP3A4*
	**IUPAC Name**: 4,6(1H,5H)-Pyrimidinedione, 5-ethyldihydro-5-phenyl-; 5-Ethyldihydro-5-phenyl-4,6(1H,5H)-pyrimidinedione	
	**Molecular Formula:** C_12_H_14_N_2_O_2_	
	**Molecular Weight:** 218.25 g/mol	
	**Mechanism:** Decreases neuron excitability, raises seizure threshold similar to phenobarbital. Primidone has two active metabolites, phenobarbital and phenylethylmalonamide	
	**Effect:** Anticonvulsants; Barbiturates	
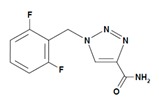	**Name: Rufinamide**	**Mechanistic genes:** *MAPK10, SCN1A* **Metabolic genes:** ** Substrate:** *CES* ** Inhibitor:** *CYP2E1* ** Inducer:** *CYP3A4*
	**IUPAC Name**: 1-[(2,6-difluorophenyl)methyl]-1H-1,2,3-triazole-4 carboxamide	
	**Molecular Formula:** C_10_H_8_F_2_N_4_O	
	**Molecular Weight:** 238.2 g/mol	
	**Mechanism:** The precise mechanism(s) by which rufinamide exerts its antiepileptic effect are unknown. The results of in vitro studies suggest that the principal mechanism of action of rufinamide is modulation of the activity of sodium channels and, in particular, prolongation of the inactive state of the channel. Rufinamide (≥ 1 μM) significantly slowed sodium channel recovery from inactivation after a prolonged prepulse in cultured cortical neurons, and limited sustained repetitive firing of sodium-dependent action potentials	
	**Effect:** Anticonvulsants; Triazole Derivative	
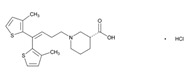	**Name: Tiagabine**	**Mechanistic genes:** *ABAT* **Metabolic genes:** **Substrate:** *CYP3A4* **Transporter genes:** *SLC6A1*
	**IUPAC Name**: 3-Piperidinecarboxylic acid, 1-[4,4-bis(3-methyl-2-thienyl)-3-butenyl]-, hydrochloride, (R)-; (-)-(R)-1-[4,4-Bis(3-methyl-2-thienyl)-3-butenyl]nipecotic acid, hydrochloride.	
	**Molecular Formula:** C_20_H_25_NO_2_S_2_ HCl	
	**Molecular Weight:** 412.01 g/mol	
	**Mechanism:** Exact mechanism not definitively known; however, in vitro experiments demonstrate that it enhances activity of gamma-aminobutyric acid (GABA). It is thought that binding to GABA uptake carrier inhibits uptake of GABA into presynaptic neurons, allowing availability of increased amount of GABA to postsynaptic neurons. Based on in vitro studies, tiagabine does not inhibit uptake of dopamine, norepinephrine, serotonin, glutamate, or choline	
	**Effect:** Anticonvulsants	
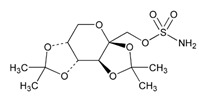	**Name: Topimarate**	**Mechanistic genes:** *ACSL4, AGXT, CA1-5, GABRA1, IGF1, KCNH2, NR1I2, SCN1A, SCN2A* **Metabolic genes:** **Substrate:** *CYP3A4* **Inhibitor:** *CYP2C19* **Inducer:** *ABCB1, CYP3A4* **Transporter genes:** *ABCB1*
	**IUPAC Name**: β-D-Fructopyranose, 2,3:4,5-bis-O-(1-methylethylidene)-, sulfamate; 2,3:4,5-di-O-isopropylidene-β-D-fructopyranose sulfamate	
	**Molecular Formula:** C_12_H_21_NO_8_S	
	**Molecular Weight:** 339.36 g/mol	
	**Mechanism:** Blocks neuronal voltage-dependent sodium channels, enhances GABA(A) activity, antagonizes AMPA/kainate glutamate receptors, and weakly inhibits carbonic anhydrase	
	**Effect:** Anticonvulsants	
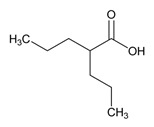	**Name: Valproic acid**	**Mechanistic genes:** *ABAT, ACADSB, ALDH5A1, HDAC2, HDAC9, OGDH, PPARA, PPARD, PPARG* **Metabolic genes:** **Substrate:** *CYP1A1, CYP1A2, CYP2A6, CYP2B6, CYP2C8, CYP2C9, CYP2C18, CYP2C19, CYP2E1, CYP3A4, CYP3A5, CYP4B1, CYP4F2, PTGS1, UGT1A1, UGT1A3, UGT1A4, UGT1A6, UGT1A8, UGT1A9, UGT1A10, UGT2B7, UGT2B15* **Inhibitor:** *CYP2A6, CYP2C9, CYP2C19, CYP2D6, CYP3A4, HDAC9, UGT1A9, UGT2B7, UGT2B15* **Transporter genes:** *ALB, SLC16A1, SLC22A5, SLC22A6, SLC22A7, SLC22A8, SLCO2B1*
	**IUPAC Name**: Pentanoic acid, 2-propyl-; (2) Propylvaleric acid	
	**Molecular Formula:** C_8_H_16_O_2_	
	**Molecular Weight:** 144.21 g/mol	
	**Mechanism:** Causes increased availability of gamma-aminobutyric acid (GABA), to brain neurons or may enhance action of GABA or mimic its action at postsynaptic receptor sites	
	**Effect:** Anticonvulsants, Miscellaneous. Antimanic Agents; Histone Deacetylase Inhibitor	
